# Effect of Elevated Air Humidity on the Structure and Proton Conductivity of Porphyrin-Based Zr(IV)-MOFs

**DOI:** 10.1021/acs.inorgchem.5c02165

**Published:** 2025-07-29

**Authors:** Jan Hynek, Matouš Kloda, Miroslava Litecká, Anna Vykydalová, Jakub Tolasz, Miroslav Pospíšil, Zuzana Morávková, Mandeep K. Chahal, Ludvík Beneš, Tomáš Plecháček, Klára Melánová

**Affiliations:** † Institute of Inorganic Chemistry of the Czech Academy of Sciences, Husinec-Řež 1001, 25068 Řež, Czech Republic; ‡ Polymer Institute, Slovak Academy of Sciences, Dúbravská Cesta 9, 84541 Bratislava, Slovakia; § Department of Chemical Physics and Optics, Faculty of Mathematics and Physics, 138735 Charles University, Ke Karlovu 3, 12116 Prague, Czech Republic; ∥ Institute of Macromolecular Chemistry of the Czech Academy of Sciences, Heyrovského nám. 2, 162 06 Prague, Czech Republic; ⊥ School of Chemistry and Forensic Science, 2240 University of Kent, CT2 7NH Canterbury, U.K.; # Center of Materials and Nanotechnologies, Faculty of Chemical Technology, 48252 University of Pardubice, Studentská 95, 53210 Pardubice, Czech Republic

## Abstract

Metal–organic frameworks (MOFs) based on Zr_6_(μ_3_-O)_8_ oxometallic clusters are attracting attention as potential proton conductors due to their high surface area, ease of further substitution, and exceptional chemical stability. We hereby present an examination of two Zr­(IV)-MOFs with a tetrakis­(4-carboxyphenyl)­porphyrin (TCPP^4–^) linker, PCN-222 and PCN-224, as proton conductors. It was found that, in spite of their excellent stability in aqueous suspensions, in the environment of elevated air humidity, serious changes in their bonding system occur, mainly involving breakage of the carboxylate coordination bonds and hydration of the Zr_6_(μ_3_-O)_8_ clusters, which leads to gradual amorphization and loss of porous character. The stability of the structures can be improved by postsynthetic modification with diphenylphosphinic acid (DPPA) to some extent. Inclusion of host imidazole molecules facilitates proton mobility in the pore system of the MOFs, further accelerating the structural degradation. Even though the original structures of the MOFs collapse under the conditions of proton conductivity measurement, the resulting amorphous solids still reveal a proton conductivity up to 6.7 × 10^–6^ S·cm^–1^ at ambient temperature and a 92% relative humidity, which is comparable to that of other Zr­(IV)-MOFs with well-preserved structures. The presented study demonstrates an important phenomenon that has to be considered with any investigation using MOFs as proton conductors.

## Graphical abstract

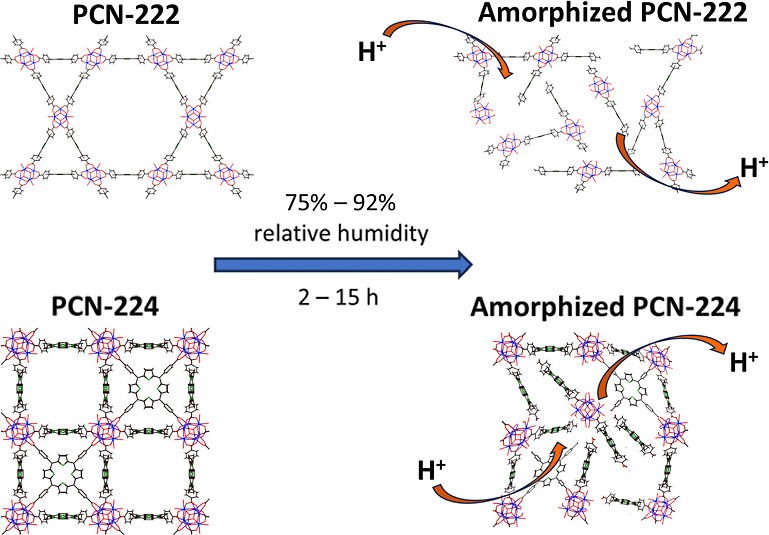

## Introduction

Proton conductive materials are a widely studied group of chemical entities, mainly for the construction of proton-exchange membranes, a key component of proton-exchange membrane fuel cells (PEMFCs).^1^ Among studied proton conductive materials, metal–organic frameworks (MOFs) attract significant attention due to their high level of order, porous structure, and possibility of tuning the pore size and chemical character of the pore surface.^2^ The proton conductivity of MOFs is usually too low to be suitable for practical applications; therefore, several techniques are used for enhancement of the properties. They involve mainly introduction of free noncoordinated polar functional groups that increase the proton concentration and mobility inside the structure, e.g., sulfonates,^3^ phosphonates,^4^ carboxylates, amines, hydroxyl groups,^5^ etc. Alternatively, proton conductive MOFs can be based on linker molecules with only one type of functional group, a part of which remains uncoordinated^6^ or where the donor groups still contain a dissociable proton.^7^

When MOFs are used as proton conductors, their pore system is usually filled with small, highly polar protic molecules that facilitate the proton mobility, such as imidazole,^8^ nonvolatile strong acids,^9^ or simply water.^10^ To achieve the highest possible proton conductivity, conditions involving elevated temperature, high relative humidity, and the presence of other polar molecules enhancing the proton mobility are used.^11^ For that reason, stability of MOFs under such conditions is a crucial factor that decides about their applicability as proton conductors.^12^

Zr­(IV)-MOFs represent a large group of materials composed of Zr_6_(μ_3_-O)_8_ secondary building units (SBUs) interconnected by various carboxylate ligands. Since 2008, when the first member of the family, UiO-66, was reported, they have been attracting growing attention.^13^ The group of Zr­(IV)-MOFs is highly structurally variable; ligands of different sizes, geometries, and number of coordinating groups (topicity) can be used, providing frameworks with various topologies and connectivity of the clusters.^14^ Apart from the carboxylate linkers, there are usually also water molecules coordinated on the Zr_6_(μ_3_-O)_8_ clusters that can facilitate proton mobility by themselves or can be replaced by other hydrophilic molecules, which makes Zr­(IV)-MOFs suitable candidates for proton conductive materials.^15^ Up to now, proton conductivity was studied on numerous Zr­(IV)-MOFs, mainly UiO-66,^16–19^ UiO-67,^20,21^ an isoreticular MOF containing larger pores, or MOF-808 containing tritopic benzene-1,3,5-tricarboxylate linkers.^22,23^ In general, the list of Zr­(IV)-MOFs that have been employed as proton conductive materials is still limited to structures containing relatively small linker molecules, and usage of bigger ligands is still quite rare. The only example of using a Zr­(IV)-MOF with bigger pores as a proton conductor was reported by Yang et al., who utilized the structure of PCN-222/MOF-545 (hereafter abbreviated as PCN-222) based on tetrakis­(4-carboxyphenyl)­porphyrin (TCPP^4–^) ligands and containing characteristic hexagonal pores with 3.7 nm diameter.^24^ In spite of the undoubtedly rich possibilities of encapsulation of various host molecules promoting proton conductivity into the MOFs with bigger pores, to the best of our knowledge, there are no other examples of any other Zr­(IV)-MOFs with pore size ≥2.0 nm being used as a proton conductor.

Zr­(IV)-MOFs are generally considered highly chemically stable, sustaining a water environment of a wide pH range.^25^ However, it was found that even though the structure and porous character of Zr­(IV)-MOFs are preserved upon treatment by an aqueous environment, numerous defects in the structure can be created.^26,27^ There are also significant differences in the stability of particular Zr­(IV)-MOFs in an aqueous environment. Recently, it was found that the main factor affecting the stability of MOFs is the ligand topicity; MIP-200 and PCN-222, which are based on tetratopic ligands, reveal much higher stability than UiO-66 and MOF-808, based on ditopic and tritopic ligands, respectively.^28^ Although the majority of Zr­(IV)-MOFs display good-enough stability in an aqueous environment, the structures of some of them (e.g., NU-1000, UiO-67, or PCN-222) do not survive direct drying from water suspensions.^29^ Since this slight change in the conditions of the treatment leads to a significantly different behavior of the materials, it is obvious that apart from the hydrolysis of coordination bonds, the structure can also be damaged by capillary forces of water released from the pores. The stability of porphyrin Zr­(IV)-MOFs in a humid environment can be enhanced by grafting more hydrophobic organic ligands (e.g., diphenylphosphinic acid (DPPA),^30^ perfluorocarboxylic acids,^31^ or octadecylphosphonic acid^32^) onto the Zr_6_(μ_3_-O)_8_ clusters. Anyway, the stability of Zr­(IV)-MOFs is a very complex issue and has to be examined for any individual case, considering the exact conditions that the material is supposed to be exposed to.

Because of the recently detected excellent stability of PCN-222 with a hexagonal structure (Figure 1) in aqueous environments,^33,34^ along with the exceptionally big pore size allowing the accommodation of guest molecules that facilitate proton mobility and rich synthetic possibilities for additional substitution of the TCPP moieties, we decided to examine the plausibility of using this material and the related cubic PCN-224 ^35^ as proton conductors. We tested their stability upon increased air humidity that corresponds to common operating conditions of proton conductors,^36,37^ described the ongoing changes to the materials, and measured the proton conductivity in time. Apart from the neat materials, we also tested the stability of their analogues with grafted diphenylphosphinic acid (DPPA), which is known for enhancing the hydrolytic stability of the material,^37^ and adsorbed imidazole (Im), which promotes the mobility of protons.

**Figure 1:**
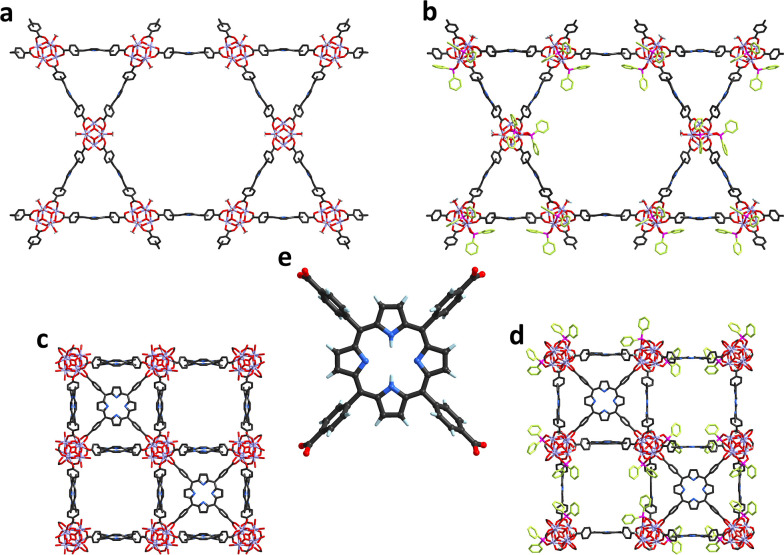
Structures of the MOFs selected for stability and proton conductivity examination: (a) PCN-222, (b) PCN-222+DPPA, (c) PCN-224, (d) PCN-224+DPPA, and (e) structure of the TCPP linker. The DPPA moieties are colored green.

## Experimental Section

### Materials and Instrumental Methods

A list of used materials and details of the instrumental methods are given in the Supporting Information.

### Preparation of PCN-222

PCN-222 was prepared according to a modified method published earlier.^30^ A Teflon-lined stainless-steel autoclave (Berghof DAB-3) was charged with 300 mg of ZrOCl_2_·8H_2_O (0.931 mmol), mixed with 80 mL of DMF, and sonicated for 30 min. After that, 232 mg of TCPP (0.293 mmol) and 40 mL of formic acid were added, and the mixture was sonicated for an additional 10 min. The mixture was heated to 130 °C under autogenous pressure for 72 h. After cooling to room temperature, the solid product was collected by centrifugation (Hettich Rottina 380R, 11,000 rpm, 10 min) and washed with DMF and acetone three times. Finally, the material was immersed in acetone for 24 h, collected by centrifugation, and vacuum-dried at room temperature.

### Preparation of PCN-224

PCN-224 was prepared according to a modified method published earlier.^35^ A 20 mL Wheaton vial was charged with 50 mg (0.063 mmol) of TCPP, 2.0 g (63.2 mmol) of benzoic acid, and 150 mg (0.644 mmol) of ZrCl_4_ and dissolved in 10 mL of DMF. The mixture was ultrasonicated for 30 min and heated to 120 °C for 24 h. After cooling down to room temperature, the solid product was collected by centrifugation (Hettich Rottina 380R, 11,000 rpm, 10 min) and washed with DMF and acetone three times. Finally, the material was immersed in acetone for 24 h, collected by centrifugation, and vacuum-dried at room temperature.

### Postsynthetic Modification of MOFs with DPPA

The postsynthetic modification of PCN-222 and PCN-224 with DPPA was done according to a modified procedure described earlier.^30^ 200 mg of a MOF (PCN-222 or PCN-224) was suspended in a 30 mL solution of 333 mg DPPA dissolved in a 2:1 DMF/water mixture (PCN-222) or neat DMF (PCN-224). The suspension was shaken for 4 h and centrifuged (Hettich Rottina 380R, 11,000 rpm, 10 min), and the solid product was washed with DMF, water, and acetone twice (PCN-222 + DPPA) or with DMF and acetone three times (PCN-224 + DPPA). The samples were vacuum-dried at room temperature.

### Adsorption of Imidazole

The inclusion of guest imidazole molecules was done by the commonly used method of adsorption of imidazole vapors.^20^ Prior to the deposition, the MOFs were activated by heating at 80 °C under a dynamic vacuum for 24 h. After that, 100 mg of the material kept in an open vial was inserted into a sealed reaction vessel charged with 500 mg of imidazole, which was then placed into an oven preheated at 80 °C and kept there for 72 h.

### Evaluation of the MOFs’ Stability

The degradation of the MOFs’ structure in time was evaluated by the XRD determination of the relative content of the crystalline phase to the initial state. The MOFs were placed onto a powder X-ray diffraction (XRD) sample holder and incubated in the environment with a defined relative humidity (75% and 92%) at room temperature, which was achieved by keeping the samples in closed cells over saturated solutions of NaCl and KNO_3_, respectively. At certain time intervals, the samples were taken out from the cells, and XRD patterns in the 2–15° 2θ range were recorded. The area under the diffraction peaks at 4.82° and 4.54° 2θ for PCN-222 and PCN-224 samples, respectively, was integrated, and the resulting integral intensity divided by the integral intensity at *t* = 0 min was plotted in the respective graphs depicting the amorphization of the MOFs at certain humidity conditions (Figure 3).

### Proton Conductivity Measurement

Prior to the preparation of the samples for proton conductivity measurements, the powdered materials were incubated in the environment of relative humidity of 75% and 92%, achieved by keeping them in closed vessels over the saturated solutions of NaCl and KNO_3_, respectively, for 6 or 48 h. After that, the powdered MOFs were pressed into a round pellet with a thickness *L* of approximately 1 mm and a diameter of 5 mm using a pressure of 46 MPa, to which Au-coated stainless-steel electrodes were mechanically pressed. The conductivity measurements were performed at room temperature with controlled air humidity, corresponding to that used for the incubation. The measurements were repeated in time. Conductivity of the samples was measured with a Metrohm Autolab PGStat12 instrument in a frequency range from 0.1 Hz to 1 MHz with a signal amplitude of 200 mV. The impedance data in a complex impedance plot were analyzed by an equivalent circuit approach using ZSimpWin software.^38^ The chosen equivalent electrical circuit used for fitting consisted of a parallel arrangement of the resistance *R* and a constant phase element (CPE), as defined by Barsoukov and Macdonald.^39^ The fit provides the value of the resistance *R*. For the calculation of the proton conductivity σ of the samples, the relationship σ = *L*/*RA*, where *A* is the area of the pellet and *L* stands for its thickness, was used.

### Molecular Modeling

Molecular mechanics and classical molecular dynamics simulations of the structure of PCN-224 + DPPA were performed using the Materials Studio modeling environment^40^ according to a previously reported procedure.^30^ The crystal structure of the framework was taken from the crystallographic database^41^ and geometrically optimized within the Universal force field.^42^ The cell parameters were *a* = *b* = *c* = 38.8372(12) Å, α = β = γ = 90°, the space group was *Im*3̅*m* (229), and the crystal symmetry was subsequently changed to P1 for calculation purposes. The DPPA molecule was constructed and geometrically optimized as an isolated molecule within the Compass force field^43^ with *E*_total_ = 47.507 kcal·mol^–1^. Since elemental analysis indicated that each SBU contained 2.6 DPPA molecules, calculated structural models containing 8 SBUs and 1, 2, and 3 DPPA molecules bonded to each SBU were made. Two binding modes of DPPA molecules were applied: (a) bridging coordination, where the oxygen atoms of DPPA were bonded to two adjacent Zr atoms, and (b) chelating coordination, where both oxygen atoms were bonded to the same Zr atom of the SBU. The charges were computed using the Qeq (charge equilibrium approach) method using zero initial charge.^44^ The structural models were geometrically optimized using the Universal force field. Atomic positions in PCN-224 were kept rigid, whereas those of DPPA molecules were kept variable. Electrostatic interactions were calculated using the Ewald method,^45^ and van der Waals energies were determined using a Lennard-Jones potential with a cutoff distance of 12.5 Å. Quench molecular dynamics was performed in an *NVT* statistical ensemble (constant number of particles, volume, and temperature) at 298 K, with a time step of 1 fs over 10^6^ steps. Total energies for all optimized structural models were calculated as a sum of the bonded interactions (bond stretching, bond angle bending, dihedral angle torsion, and inversion) between the DPPA molecules and the SBUs and nonbonded interactions (electrostatic and van der Waals terms) within the DPPA molecules, between neighboring DPPA molecules, and between the DPPA molecules and the framework. In order to decide about the validity of the structural models, total energies (*E*_total_) for each DPPA molecule on an SBU (Table S2) were calculated using the following formula

1 $$E_{total}=\frac{\left(E_{i}-E_{j}\right)}{\left(n_{i}-n_{j}\right)}$$

*E*_*i*_ stands for the total energy of the model containing *i* DPPA molecules on each SBU, *E*_*j*_ is the total energy of the model containing *j* DPPA molecules per SBU, and *n*_*i*_ and *n*_*j*_ are the total numbers of DPPA molecules in the corresponding structural models. Since all created models contained 8 SBUs and the calculations were made for (*i*,*j*) = (1,0), (2,1), and (3,2) couples, (*n*_*i*_ – *n*_*j*_) was always 8. Energy of the neat PCN-224 framework was not included in the calculations; *E*_0_ was considered to be 0.

## Results and Discussion

### Synthesis and Characterization

PCN-222 was prepared using a published procedure,^30^ and the recorded powder XRD pattern (Figure S1) confirmed the structure and phase purity of the obtained material.^24^ The cubic TCPP-based Zr­(IV)-MOF was prepared following the synthetic protocol, which is reported to provide PCN-224.^35^ There are three reported TCPP-based Zr­(IV)-MOFs with a cubic structure, PCN-221,^46^ PCN-224,^35^ and MOF-525,^34^ which are very similar to each other, and they are reported to be produced by similar synthetic protocols. However, Koschnick et al. published a detailed structural study of those materials, which shows that the resulting product has probably always had the same structure, and the only difference is the level of disorder. They concluded that the structural model of PCN-224 is closest to reality.^47^ For that reason, we denote our material as PCN-224. Therefore, the experimental powder XRD pattern (Figure S2) is in good agreement with the structural model, and the missing superstructure reflections at 3.2° and 5.6° 2θ in the pattern suggest that, in contrast to the model, there is no regular ordering of TCPP linker vacancies in the obtained material.

The postsynthetic modification of MOFs with DPPA was carried out to increase the robustness of the studied MOFs according to the procedure previously reported for PCN-222.^30^ In the case of PCN-224, the solvent was exchanged for neat DMF because water was found to have a negative effect on the crystallinity of PCN-224. The respective powder XRD patterns (Figures S3 and S4) reveal that the postsynthetic modification does not cause any changes to the framework structure. According to the elemental analyses (Table S1), the content of phosphorus is 1.8–2.2 wt % that corresponds to approximately 2 DPPA molecules attached to one Zr_6_(μ_3_-O)_8_ cluster in PCN-222 and 2.6 DPPA molecules per one cluster in the case of PCN-224. The presence of coordinated DPPA molecules was also confirmed by FTIR spectra (Figures S5 and S6), where characteristic signals of P–O–Zr vibrations^48^ at 1122 and 995 cm^–1^ appeared.

The inclusion of imidazole guests was carried out to increase the proton mobility in the studied materials. The presence of imidazole in the imidazole-loaded samples could not be evidenced by FTIR spectra due to the overlap with peaks belonging to porphyrin; however, it was confirmed by the thermal analyses under anaerobic conditions (Figures S7–S14) coupled with mass spectrometry, where the evolved gas contained imidazole (*m*/*z* = 68) at temperatures above 200 °C. The presence of imidazole also resulted in an increased weight percentage of nitrogen in comparison with their imidazole-free analogues. Based on the respective differences in the N content, the number of adsorbed imidazole molecules per one Zr_6_(μ_3_-O)_8_ cluster was calculated to be between 1.3 and 1.9.

### Molecular Modeling

To clarify the arrangement of DPPA molecules bonded to the SBUs in PCN-224, structural models considering 1, 2, and 3 DPPA molecules bonded to each SBU in either bridging (Figure S15) or chelating (Figure S16) coordination mode were built. The calculated values of the total energy per DPPA molecule (Table S2) suggest that in the case of the first and second DPPA molecules, the bridging binding mode is preferred. When a third DPPA molecule is bonded to an SBU, the total energy of the system rapidly increases (from 92 to 356 kcal·mol^–1^) due to steric hindrance among the bonded DPPA molecules and their higher electrostatic repulsions. Therefore, we expect that each SBU becomes substituted by 2 DPPA molecules and the average content of 2.6 DPPA molecules per one SBU suggested by elemental analysis can originate from the higher DPPA content on more accessible defective SBUs and from DPPA molecules covering the surface of crystallites. The suggested binding mode of DPPA molecules on Zr_6_(μ_3_-O)_8_ clusters of PCN-224, the total energy of the considered system, and the expectation of binding 2 DPPA molecules onto each cluster are in good agreement with a previous theoretical study performed on PCN-222.^30^

### Adsorption Properties

Adsorption characteristics of the MOFs were determined by measurement of the adsorption isotherms of Ar at 87 K (Figure 2). The specific surface area values for PCN-222 and PCN-224 are slightly lower than the data reported in the literature, where N_2_ was used as an adsorbate.^33–35^ We would like to note that this behavior is common among MOFs, where N_2_ adsorption isotherms are known to provide higher values than adsorption isotherms of Ar (87 K) in some cases.^49^ In the case of PCN-222, the specific shape of the isotherms confirms the presence of two types of pores, smaller micropores and larger mesopores, as can be seen from the respective crystal structure. In contrast, PCN-224 reveals a purely microporous character. Postsynthetic modification of the MOFs with DPPA led to a decrease in the specific surface area by 8–20% and in the case of PCN-224 even more pronounced decrease in the surface area attributed to micropores (Table 1). Since the materials were activated at 100 °C under high vacuum before the adsorption measurement, the imidazole-loaded samples were not examined due to the volatility of imidazole.

**Figure 2:**
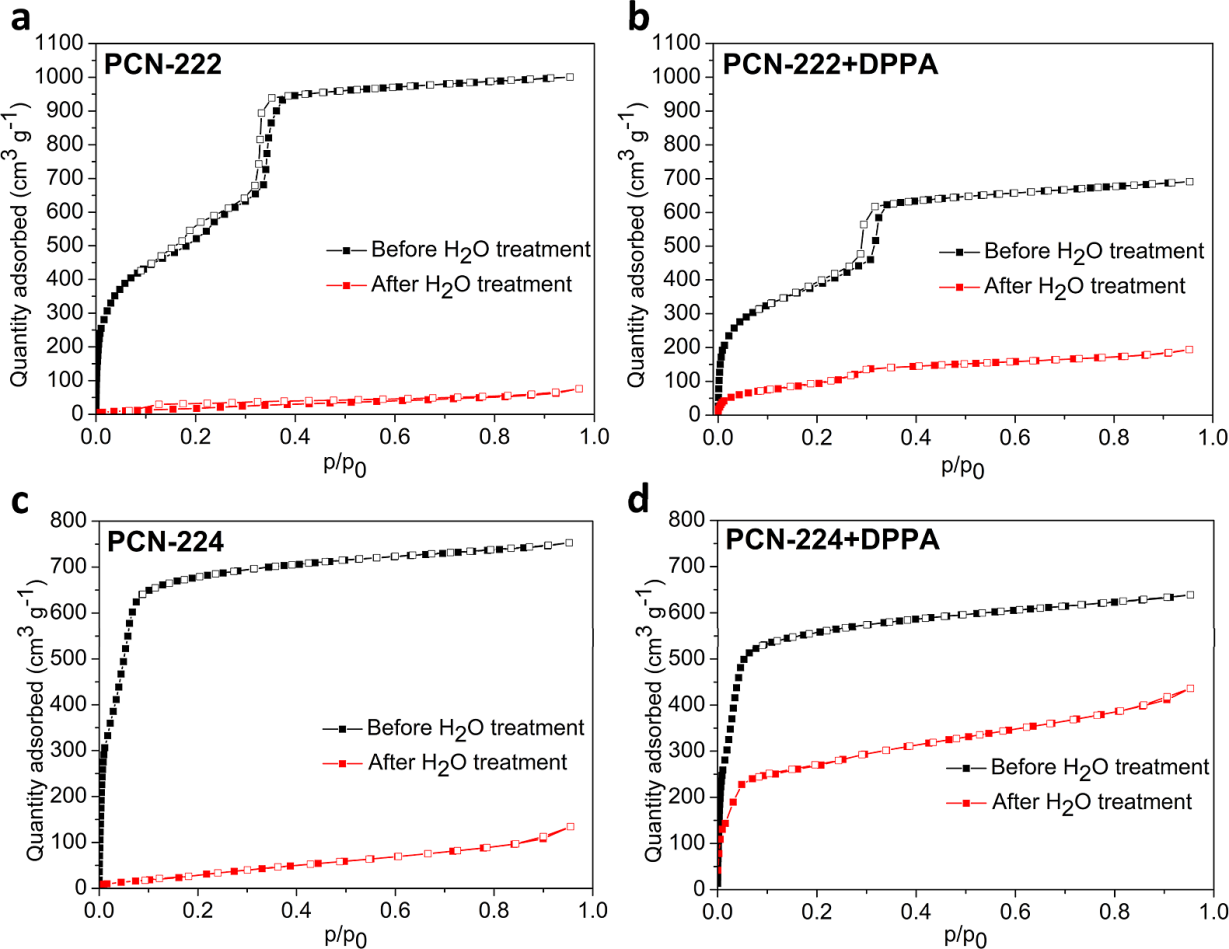
Adsorption isotherms of argon for (a) PCN-222, (b) PCN-222+DPPA, (c) PCN-224, and (d) PCN-224+DPPA before and after the measurement of water adsorption.

**Table 1:** Specific Surface Area, Pore Volume, and Adsorption Capacity for Water of the Studied MOFs

compound name	PCN-222	PCN-222+DPPA	PCN-224	PCN-224+DPPA
initial specific surface area [m^2^ g^–1^]^a^	1479	1171	2449	2105
initial external surface area [m^2^ g^–1^]^b^	88	119	212	444
initial pore volume [cm^3^ g^–1^]	1.166	0.880	0.958	0.813
adsorption capacity for H_2_O [cm^3^ g^–1^]^c^	628	765	357	637
number of adsorbed H_2_O molecules per one Zr_6_(μ_3_-O)_8_ cluster^d^	75	111	49	110
final specific surface area [m^2^ g^–1^]^a^	45	272	62	938
final external surface area [m^2^ g^–1^]^b^	45	138	62	508
final pore volume [cm^3^ g^–1^]	0.011	0.247	0.015	0.556

a BET surface area was calculated in the range from 0.005 to 0.1 *p*/*p*_0_.

b Calculated by a *t*-plot.

c Quantity adsorbed at *p*/*p*_0_ = 0.91.

d Calculated from the adsorption capacity and elemental analysis (Table S1).

Water adsorption isotherms (Figures S17 and S20) display that the materials accommodate a reasonable amount of water in the 0.6–0.9 *p*/*p*_0_ range; the introduction of more hydrophobic DPPA moieties leads to a slight shift of the adsorption region to higher relative pressures. The adsorption capacity for water is 350–770 cm^3^ g^–1^, which corresponds to 49–111 adsorbed H_2_O molecules per one Zr_6_(μ_3_-O)_8_ cluster. Interestingly, the results of water adsorption measurements pointed out unexpected behavior of the samples. The DPPA-modified MOFs demonstrated a higher amount of adsorbed water in spite of their lower specific surface area. To clarify this phenomenon, the DPPA-free samples after the measurement of H_2_O adsorption were reactivated, and the adsorption isotherms of Ar were measured again. After adsorption and desorption of water, the samples revealed only a very low adsorption of Ar (Figure 2), indicating significant changes to the material connected with almost a total loss of porosity. On the other hand, the DPPA-modified MOFs after the adsorption and desorption of water still revealed some measurable porosity; however, the amount of adsorbed Ar decreased by 30 and 75% for PCN-224+DPPA and PCN-222+DPPA, respectively (Table 1). The powder XRD patterns recorded after the measurement of H_2_O adsorption (Figures S21–S24) finally confirmed the significant damage to the frameworks caused by the treatment with water vapor. These results are in line with the gas adsorption, as PCN-224+DPPA which had the most preserved structure also retained the highest specific surface area. It is clear that the loss of porosity goes hand in hand with the loss of crystallinity.

### Stability of PCN MOFs

Although Zr­(IV)-MOFs are generally considered exceptionally chemically stable, which was recently confirmed by a detailed study of stability that showed there are only negligible changes in the structure and chemical composition of PCN-222 treated by acidic and basic solutions of pH up to 10,^28^ during the measurement of water vapor adsorption, we observed considerable changes to the materials, and therefore, we decided to study the influence of air moisture on the crystallinity in more detail. The air moisture stability of MOFs was tested at a relative humidity of 75% and 92%, which corresponds to conditions used for proton conductivity measurements and operation of PEMFCs.^50^ The materials were exposed to the appropriate relative humidity at room temperature, and powder XRD patterns were recorded at explicit time intervals. As the powder XRD patterns (Figures S25–S38) show, the intensity of diffraction peaks is gradually decreasing. From the dependence of the integral intensity of diffraction peaks on the contact time (Figure 3), it is evident that parent PCN-222 (Figure S25) is slightly more stable than PCN-224 (Figure S26); however, even in that case, 15 h of exposure to a 75% relative humidity leads to a complete amorphization of the material. At a 92% relative humidity, the effect is even more pronounced, leading to a complete loss of crystallinity of both MOFs within 4–5 h. When PCN-222 was postsynthetically modified by binding DPPA molecules onto Zr_6_(μ_3_-O)_8_ clusters, the stability of the resulting material significantly improved, even after 20 h at a 75% relative humidity; the crystallinity was almost unchanged (94% of the initial integral intensity of the diffraction peaks, Figure S27). A different behavior was observed for PCN-224+DPPA (Figure S28), where some decrease of the crystallinity took place within the first 20 h (to 66% of the initial integral intensity); however, after that, the amorphization was much less pronounced than in the initial phase. After 56 h of exposure to 75% relative humidity, the powder XRD diffractions still revealed 60% of the initial integral intensity (Figure S29).

**Figure 3:**
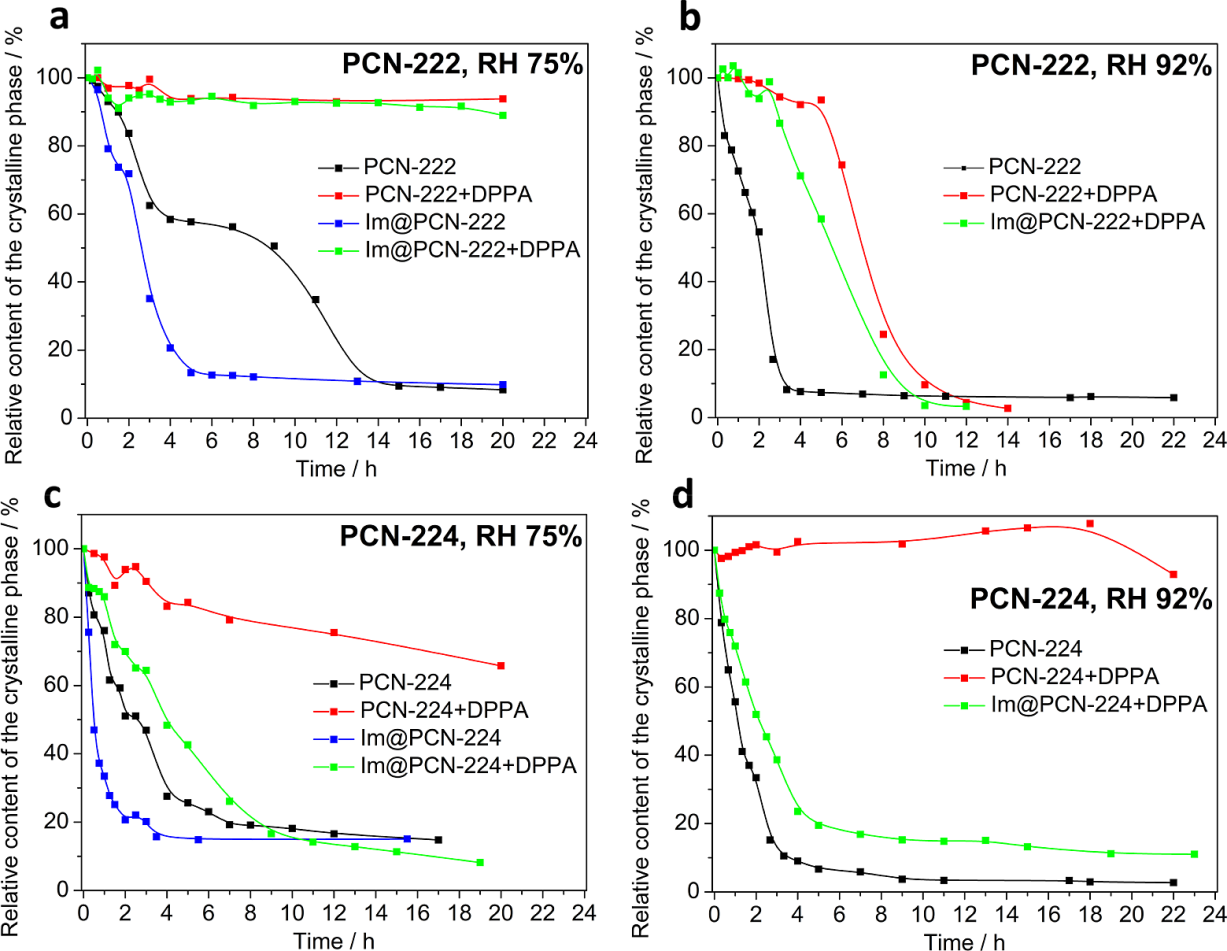
Time dependence of the relative content of the crystalline phase in PCN-222 samples at (a) 75% and (b) 92% relative humidity and PCN-224 samples at (c) 75% and (d) 92% relative humidities. The content of the crystalline phase is referenced to the initial value taken as 100%.

At the relative humidity of 92%, degradation of the studied MOFs is even faster than at 75% (Figures S30–S33). For example, complete degradation of PCN-222+DPPA at 92% relative humidity takes around 10 h (Figure S32), and at the beginning, the process seems to be slower, whereas between 6 and 10 h of contact time, there is a rapid decrease in the intensity of diffraction peaks. This might be due to the known fact that the MOFs’ structures can be preserved even when a significant amount of the linker is taken out of the framework.^28^ In this case, a continual hydrolysis of the coordination bonds probably takes place, and at a certain point, the framework is so defective that the structure suddenly collapses.

In the case of the imidazole-loaded samples, the stability is generally lower than that of their imidazole-free analogues. Im@PCN-222 becomes completely amorphous after only 5 h at a 75% relative humidity (Figure S34) in comparison with 15 h that is needed for the neat PCN-222. In the case of Im@PCN-224, 3 h of exposure to 75% humidity was enough for the transformation to a completely amorphous solid (Figure S35), whereas neat PCN-224 after the same contact time still revealed 47% of the initial diffraction intensity. A negative effect of the imidazole presence on the MOF crystallinity can also be demonstrated by the lower quality of the powder XRD patterns of the corresponding materials at the beginning of the stability tests. Due to the very fast degradation of imidazole-loaded materials at a 75% humidity, it was already unnecessary to evaluate the influence of 92% humidity. The stability of the samples containing both imidazole and DPPA lies between that of the neat MOFs and DPPA-modified MOFs. The degradation curves demonstrate generally lower stability of PCN-224 in comparison to PCN-222; while Im@PCN-222+DPPA can survive exposure to 75% humidity (Figure S36), Im@PCN-224+DPPA becomes almost amorphous (15% of the initial integral intensity remaining) within 11 h (Figure S37). At a 92% humidity, both samples are completely degraded in 6–7 h (Figures S38 and S39).

To sum up, the postsynthetic modification of MOFs with DPPA significantly improved their resistance to air moisture. On the other hand, inclusion of hydrophilic imidazole, which would promote the mobility of protons, has an opposite effect. However, the stability of PCN-222+DPPA and Im@PCN-222+DPPA at a 75% relative humidity seems to be high enough to enable their practical use. PCN-224+DPPA is the only sample of all tested that can withstand the conditions of 92% relative humidity. Even when the exposure to humid air was prolonged to 56 and 84 h, PCN-224+DPPA still contained more than 60% of the original amount of the crystalline phase (Figure S40), which is in sharp contrast with the majority of tested materials that reveal complete amorphization within 2–15 h.

### Structural Changes Caused by the Exposure to Increased Air Humidity

To understand the chemical changes caused to the materials by exposure to increased air humidity, FTIR and Raman spectra of neat PCN-222 and PCN-224 before and after the exposure to 92% relative humidity for 24 h were measured, compared, and analyzed in detail. FTIR spectra of the samples were measured by using two techniques. Measurement on ATR crystals was predominantly chosen because there is no risk of sample disturbance caused by the applied pressure and blending with KBr, influencing mainly the fine hydrogen bonding within the MOFs. However, since the Zr–O vibration was out of the transmission window of the diamond crystal, the samples were also pressed in KBr pellets and measured in transmission mode to obtain a full overview of the vibrations of Zr_6_(μ_3_-O)_8_ clusters.

The FTIR spectrum of PCN-222 measured in KBr pellets (Figure S41) shows that the main characteristic vibration bands of Zr_6_(μ_3_-O)_8_ clusters, which are at 720 cm^–1^ and around 658 cm^–1^ (dehydroxylated and hydroxylated μ_3_–O stretching, respectively), at 635 and 573 cm^–1^ (Zr–OC stretching), and at 477 cm^–1^ (μ_3_–OH stretching) (Table S3)^51,52^ are still present in the samples after degradation. This indicates that the oxometallic clusters sustain the exposure to increased air humidity. The observed minor shifts of the bands are probably caused by drying and distortion of the sample upon blending with KBr.

In the high-frequency region of the ATR-FTIR spectrum of PCN-222 (Figure S42), a dramatic increase in the intensity of the water OH stretching band around 3210 cm^–1^ in the degraded sample that disappears upon drying can be observed, which suggests that the degradation is accompanied by adsorption of a significant amount of water.^53,54^ The sharp peak at 3674 cm^–1^ corresponding to the O–H stretching vibration of hydroxyl groups within the cluster^52,55,56^ cannot be detected in degraded PCN-222, indicating strong hydrogen bonding of adsorbed water molecules with the cluster. Unlike in a similar experiment performed on UiO-66,^52^ the vibration cannot be recovered by drying. The N–H stretching band of the TCPP linker^51^ at 3314 cm^–1^ is shifted to 3306 cm^–1^ upon degradation and goes back after drying. The irreversible disappearance of a small peak at 2900 cm^–1^ belonging to the C–H stretching vibration of free formic acid^51^ indicates that formate ligands originally coordinated to Zr_6_(μ_3_-O)_8_ clusters are lost from the material upon exposure to increased humidity.

In the spectral region that belongs to stretching vibrations of carboxylic acid groups (Figure S43), significant changes can be observed. The carbonyl stretching band of protonated acid groups (−COOH) at 1720 cm^–1^ increased and shifted to 1695 cm^–1^ upon degradation; after drying, two maxima at 1710 and 1695 cm^–1^ are observed, which reveals that the degradation involves breaking of the coordination bonds between the clusters and carboxylate groups, which become protonated and solvated by the adsorbed water molecules afterward. Drying of the sample causes part of the carboxylate groups to be coordinated again, however, probably in a monodentate manner (Table S3). The structured band with a main maximum at 1598 cm^–1^ related to asymmetric stretching of carboxylate groups and aromatic ring stretching vibrations^53,57^ is shifted to 1579 cm^–1^, which is probably caused by the presence of free carboxylates. A new strong maximum that appears at 1536 cm^–1^ also demonstrates the presence of bidentally coordinated carboxylate groups in the degraded PCN-222. The band at 1408 cm^–1^ corresponding to the symmetrical stretching of bridging carboxylate groups with some contribution of the pyrrole ring stretching^53,57^ is shifted to 1367 cm^–1^ due to the carboxylate decoordination, and a shoulder that can be seen at the higher wavenumber side also suggests that a small amount of bidentate coordinated carboxylates is present in the sample. Broadening and intensity increase of the small peaks at 1305 and 1278 cm^–1^ belonging to the C–O stretching in protonated acid groups as well as pyrrole ring stretching are rather assigned to partial protonation of pyrrole nitrogen atoms.

In the 750–810 cm^–1^ region (Figure S44) related to bending vibrations of the carboxylate anion and protonated acid carbonyl,^53^ there are five maxima in the spectrum of fresh PCN-222. However, after degradation, only two broad bands at 795 and 767 cm^–1^ can be detected. Upon drying, the latter band shifts back to 770 cm^–1^. Although exact attribution of the individual peaks cannot be made, the observed changes indicate some distortion of the carboxylate coordination. The two bands at 710 and 721 cm^–1^, corresponding to the Zr–OC stretching, are broadened and shifted, which indicates that some carboxylates are still coordinated to the cluster; however, the coordination sphere is rather distorted. The band at 733 cm^–1^, connected with the Zr–(μ_3_-O) vibration of OH-free clusters, completely disappears.^52^

The FTIR spectra of PCN-224 before and after exposure to a 92% relative humidity reveal changes with similar features to PCN-222; however, particular differences can be found. The bands connected with the vibrations of Zr_6_(μ_3_-O)_8_ clusters (Figure S41) at 720 cm^–1^ and around 658 cm^–1^ (dehydroxylated and hydroxylated μ_3_–O stretching, respectively) are also preserved in the degraded PCN-224; however, their ratio differs from the degraded PCN-222, which points out a lower degree of cluster hydration. The lower intensity of the broad peak around 3200 cm^–1^ in the region of hydrogen bonding vibrations (Figure S42) demonstrates that the hydration of clusters is less pronounced than that in the case of degraded PCN-222. Even in the as-prepared PCN-224, the sharp peak of the OH stretching of hydroxyl groups within the cluster (μ_3_–OH) at 3674 cm^–1^ is missing, which points out that at the coordination sites of the cluster unoccupied by TCPP linkers, benzoate moieties are preferentially coordinated instead of water molecules. In the region of carboxylic acid group stretching vibrations (Figure S43), degraded PCN-224 displays a higher fraction of nondissociated carboxylate coordination bonds (higher intensity of 1713 cm^–1^ and lower intensity of 1603 and 1584 cm^–1^ bands) and lower content of residual DMF (1650 cm^–1^). On the other hand, the fraction of free carboxylate groups (bands at 1584 and 1367 cm^–1^) is significantly smaller than in degraded PCN-222, which goes hand in hand with the lower water content. In contrast to PCN-222, the changes in the region of Zr–O stretching vibrations at 700–750 cm^–1^ (Figure S44) cannot be properly evaluated in the case of PCN-224 as the bands are overlapped by the signals of the benzoate moieties (monosubstituted benzene ring CH deformation band). In general, the bonding system of PCN-224 seems to be less affected by the exposure to an increased air humidity than PCN-222.

In the Raman spectra of as-prepared and degraded PCN-222 and PCN-224 (Figures S45 and S46, respectively), only the vibrations of TCPP linkers can be detected.^53,57,58^ Signals of the Zr_6_(μ_3_-O)_8_ cluster and its surroundings^59^ are masked by the fluorescence of the porphyrin core. Several vibration bands of TCPP become shifted upon incorporation into the MOFs (Table S4). After degradation, they occur closer to the positions in neat TCPP, which demonstrates partial release of the restraints in their movement due to the partial breakage of the coordination bonds upon degradation.

In general, the vibrational spectra of the porphyrin-based Zr­(IV)-MOFs after the exposure to 92% relative humidity suggest that the observed degradation of the structures is caused mainly by decoordination and partial detachment of the carboxylate groups from the Zr_6_(μ_3_-O)_8_ clusters, accompanied by hydration of both the carboxylates and the clusters. These changes are more pronounced in the case of PCN-222; however, even in the case of PCN-224, the effect is strong enough to cause a collapse of the entire structure. The lower connectivity of Zr_6_(μ_3_-O)_8_ clusters in PCN-224 can also play an important role. The oxometallic clusters are still present in the resulting amorphous solids; however, the original structure cannot be restored by drying because although the carboxylate groups become recoordinated to the clusters after the removal of water, they adopt random coordination modes; apart from the original bridging type, monodentate or bidentate coordination to a single Zr atom of the cluster can also be detected.

Apart from the crystallinity and bonding system of the materials, exposure to an increased air humidity also affects their morphology. According to the scanning electron microscopy images, the neat PCN-222 as well as the DPPA-modified material contain hexagonal rod-like particles of approximately 1–1.5 μm length (Figure S47). Upon the 24 h exposure to a 92% relative humidity, the particles of the neat PCN-222 retain their size and shape; however, their edges become smoother. On the other hand, there are no detectable changes in the morphology of PCN-222+DPPA upon exposure to an increased air humidity, correlating with the higher stability of the DPPA-modified MOFs. In the case of PCN-224, the effect is even more pronounced. The as-prepared material is composed of well-developed cubic microcrystals of 300–500 nm size, whereas after exposure to a 92% humidity, the particles exhibit elongated morphology and show a collapse of the cubic morphology inward, the relatively smooth surface sides appear to be dented, and the edges of the cubes are uneven (Figure S48). As in the case of PCN-222, the particles of the DPPA-modified PCN-224 do not reveal any changes in size and morphology upon exposure to an increased air humidity.

### Relationship between the Composition, Structure, and Proton Conductivity

To evaluate the impact of the changes in the structure of MOFs on their proton conductivity, we performed impedance measurements of all prepared materials at both 75% and 92% relative humidity after incubation for 48 h in the environment of the target humidity to allow all expected structural changes to happen (Figures S49–S64). After that, the powdered samples were pressed into pellets, and their proton conductivity was measured at ambient temperature. The measurements were repeated after 2 and 24 h while keeping the same relative humidity. The repeated measurements show significant differences between the first and second measurements (Table 2 and Figure 4); however, differences between the second and third measurements were only negligible.

**Table 2:** Proton Conductivity of the Studied MOFs Measured after a Defined Time of Exposure to the Particular Air Humidity^a^

relative humidity	75%			92%		
contact time/h	48	50	72	48	50	72
PCN-222	4.5 × 10^–7^	7.3 × 10^–8^	4.2 × 10^–8^	7.4 × 10^–7^	6.4 × 10^–7^	9.4 × 10^–7^
PCN-222+DPPA	2.1 × 10^–11^	6.4 × 10^–11^	1.5 × 10^–10^	6.1 × 10^–11^	3.1 × 10^–10^	8.7 × 10^–10^
Im@PCN-222	5.0 × 10^–7^	2.8 × 10^–7^	1.1 × 10^–7^	2.2 × 10^–6^	3.3 × 10^–6^	6.7 × 10^–6^
Im@PCN-222+DPPA	2.7 × 10^–8^	5.1 × 10^–8^	4.4 × 10^–8^	6.3 × 10^–7^	1.0 × 10^–6^	1.5 × 10^–6^
PCN-224	1.4 × 10^–8^	4.3 × 10^–9^	2.3 × 10^–9^	4.0 × 10^–8^	1.9 × 10^–8^	1.7 × 10^–8^
PCN-224+DPPA	1.6 × 10^–8^	3.9 × 10^–9^	1.4 × 10^–9^	7.3 × 10^–8^	2.9 × 10^–8^	2.3 × 10^–8^
Im@PCN-224	4.5 × 10^–9^	4.2 × 10^–9^	3.9 × 10^–9^	1.6 × 10^–7^	1.9 × 10^–7^	1.7 × 10^–7^
Im@PCN-224+DPPA	2.2 × 10^–7^	7.9 × 10^–8^	4.1 × 10^–8^	2.6 × 10^–5^	2.2 × 10^–6^	1.5 × 10^–6^

a The values are given in S·cm^–1^.

**Figure 4:**
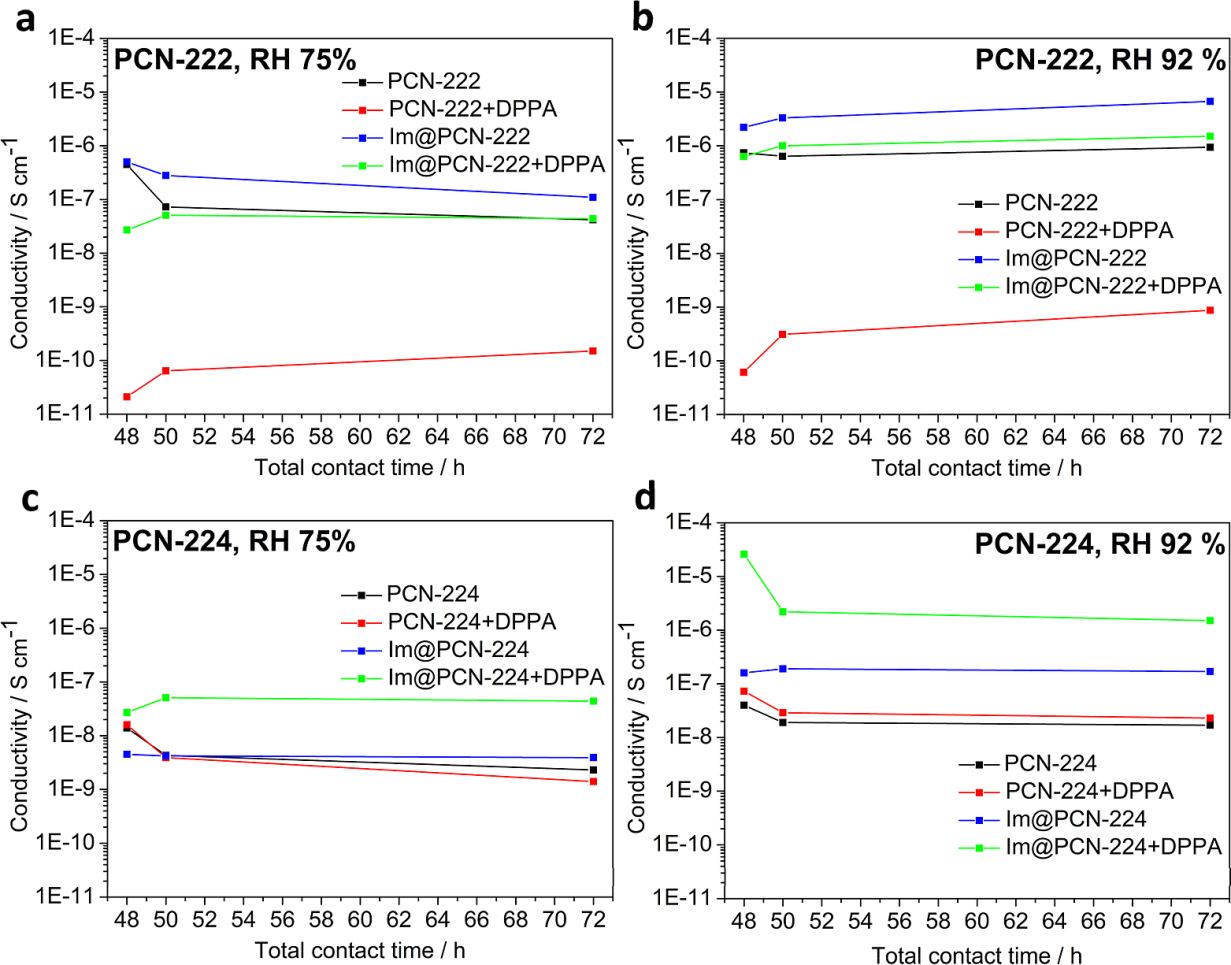
Time dependence of the proton conductivity of PCN-222 samples at (a) 75% and (b) 92% relative humidity and PCN-224 samples at (c) 75% and (d) 92% relative humidities. The total contact time represents the total time of exposure of the material to the target air humidity including the incubation before the preparation of pellets.

The neat MOFs revealed proton conductivity on the order of magnitude of 10^–9^–10^–7^ S·cm^–1^ at a 75% humidity and 10^–8^–10^–6^ S·cm^–1^ at a 92% humidity. These values are surprisingly comparable to other reported Zr­(IV)-MOFs measured at ambient temperature and similar humidity conditions,^17,60^ although the studied materials do not reveal any measurable porosity. On the other hand, during the structural degradation, a number of free carboxylic acid groups and Zr_6_(μ_3_-O)_8_ clusters with coordination sites occupied by coordinated water are released, which can lead to the enhancement of proton conductivity. Since the conductivity values obtained by repeated measurements are decreasing, the negative effect of porosity loss is probably slightly stronger.

When PCN-222 was postsynthetically modified with DPPA ligands as a protection against the negative impact of increased air humidity, its proton conductivity decreased by several orders of magnitude to approximately 10^–11^ S·cm^–1^. In the case of PCN-224, the effect of DPPA was less pronounced; however, in spite of the stability enhancement, grafting of hydrophobic moieties onto the SBUs is obviously not a suitable strategy in terms of proton conductivity. Inclusion of host imidazole molecules, which is known to increase the proton conductivity of Zr­(IV)-MOFs by up to 3 orders of magnitude, also enhanced the properties of the studied solids; however, the effect was less pronounced than in the case of the more stable Zr­(IV)-MOFs.^20^ In this case, the presence of a porous system enabling regular ordering of the guest molecules obviously plays an important role.

The powder XRD patterns of the MOFs recorded after the measurement of proton conductivity (Figures S65–S72) show that none of the samples survived the conditions of the measurement. In contrast with the results of previously performed stability tests, even PCN-222+DPPA and PCN-224+DPPA, which retained the structure even when they were exposed to a 75% relative humidity for 56 h, revealed a loss of crystallinity under the conditions of the measurement. Apparently, the ongoing amorphization is not only a matter of air humidity but also induced by the electric current. We also tried a modification of the measurement procedure to check whether gentler sample treatment can lead to better preservation of the structures. The incubation time before the preparation of pellets was changed to only 6 h, followed by a series of repeated proton conductivity measurements up to a total time of 220 h (Figures S73 and S74), until constant values of conductivity were achieved. According to the powder XRD patterns recorded after these experiments (Figures S75 and S76), it is obvious that the way of the sample preparation plays only a minor role in the actual structural changes. Since penetration of water into pellets is slower than that into the powdered sample, the process of structural degradation solely takes more time, which is demonstrated by the longer time that is necessary to achieve stabilized proton conductivity values (Table S5).

The amorphized porphyrin Zr­(IV)-MOFs display proton conductivity of up to 9.4 × 10^–7^ S·cm^–1^, which can be compared to the performance of neat UiO-66 (Table S6).^18^ Unlike PCN-222 and PCN-224, this material does not lose its porosity when exposed to increased air humidity, but on the other hand, its structure contains 12 interconnected Zr_6_(μ_3_-O)_8_ clusters and no free carboxylate groups; therefore, the structure does not offer many ways for proton transport. Another neat Zr­(IV)-MOF, MOF-808, shows a proton conductivity of about 3–4 orders of magnitude higher. The cluster connectivity in this material is 6, comparable to that of PCN-224, and moreover, it retains the porous character.^61^ If we consider these structure–property relationships, we believe that the resulting values for PCN-222 and PCN-224 are a compromise between the loss of porosity on the one hand and the release of free functional groups (e.g., carboxylate groups, hydrated Zr_6_(μ_3_-O)_8_ clusters) that increase proton mobility on the other. The relatively modest proton conductivity values of the neat Zr­(IV)-MOFs can be significantly increased by functionalization of the linkers or by introduction of small hydrophilic guest molecules (e.g., imidazole). The literature values obtained for MOF-808 suggest that inclusion of imidazole molecules into the pores leads to the enhancement of proton conductivity by more than 3 orders of magnitude.^22^ In the case of PCN-222 and PCN-224, the performance can also be improved by the inclusion of imidazole; however, the enhancement is not higher than 1 order of magnitude. This is probably connected with the loss of porosity since the host molecules are not regularly ordered in the resulting amorphous solids and cannot facilitate the proton transport so effectively.

## Conclusions

Porphyrin Zr­(IV)-MOFs, PCN-222 and PCN-224, which were previously found to be outstandingly stable in water suspensions,^30^ undergo significant structural changes under exposure to increased air humidity. The changes are probably induced by capillary condensation of water droplets in the relatively big pores of the materials^26^ and involve mainly breakage of the coordination bonds between the oxometallic nodes and the linker molecules and hydration of the released functional groups. These changes in the bonding system of the material lead to the gradual collapse of the crystal structure and loss of porosity, which is a similar behavior that was previously observed in the case of PCN-222 dried from a water suspension.^27^ At relative humidity of 75% and higher, the materials become completely amorphous within 3–15 h, which contradicts the existing beliefs of high chemical stability of these materials. The moisture stability of the materials can be significantly enhanced by the introduction of more hydrophobic diphenylphosphinate ligands onto the oxometallic nodes. On the other hand, inclusion of hydrophilic imidazole, which is a widely used agent for promotion of proton conductivity in MOFs, into the pores of the MOFs has an opposite effect. Although the resulting amorphized solids do not reveal porous character, their proton conductivity at ambient temperature is around 10^–8^ S·cm^–1^ at ambient temperature, which is comparable to some Zr­(IV)-MOFs that preserve their structure upon exposure to an increased air humidity.^18^ Probably, the loss of porous character is compensated for by the release of free hydrophilic functional groups that facilitate the proton conduction. However, in contrast with other Zr­(IV)-MOFs, inclusion of imidazole into PCN-222 or PCN-224 does not lead to a significant increase in the proton conductivity of the resulting amorphous solids, probably due to the lack of porosity.^17,49^ It is obvious that the stability of MOFs in different environments is a complex, to some extent, unpredictable issue affecting the performance of the materials in particular applications and has to be properly evaluated in any case. Particularly, the stability of Zr­(IV)-MOFs, which are generally considered “stable”, is not straightforward and needs further studies.

## Supplementary Material



## Notes

Description of used materials and instrumental methods, PXRD patterns, elemental analysis, FTIR and Raman spectra, TGA curves, molecular dynamic simulations, adsorption isotherms, SEM images, impedance curves for proton conductivity measurements, and additional proton conductivity results (PDF)

## References

[1] Liu S.-S., Liu Q.-Q., Huang S.-Z., Zhang C., Dong X.-Y., Zang S.-Q. (2022). Sulfonic and Phosphonic Porous Solids as Proton Conductors. Coord. Chem. Rev..

[2] Lim D.-W., Kitagawa H. (2021). Rational Strategies
for Proton-Conductive Metal–Organic
Frameworks. Chem. Soc. Rev..

[3] Ramaswamy P., Matsuda R., Kosaka W., Akiyama G., Jeon H. J., Kitagawa S. (2014). Highly Proton Conductive Nanoporous Coordination Polymers
with Sulfonic Acid Groups on the Pore Surface. Chem. Commun..

[4] Wei Y.-S., Hu X.-P., Han Z., Dong X. Y., Zang S. Q., Mak T. C. W. (2017). Unique Proton
Dynamics in an Efficient MOF-Based Proton
Conductor. J. Am. Chem. Soc..

[5] Shigematsu A., Yamada T., Kitagawa H. (2011). Wide Control of Proton Conductivity
in Porous Coordination Polymers. J. Am. Chem.
Soc..

[6] Kloda M., Plecháček T., Ondrušová S., Brázda P., Chalupský P., Rohlíček J., Demel J., Hynek J. (2022). Phosphinate MOFs Formed from Tetratopic
Ligands as Proton-Conductive Materials. Inorg.
Chem..

[7] Steinke F., Javed A., Wöhlbrandt S., Tiemann M., Stock N. (2021). New Isoreticular
Phosphonate MOFs Based on a Tetratopic Linker. Dalton Trans..

[8] Zhang F.-M., Dong L.-Z., Qin J.-S., Guan W., Liu J., Li S.-L., Lu M., Lan Y. Q., Su Z. M., Zhou H. C. (2017). Effect of Imidazole
Arrangements on Proton-Conductivity
in Metal–Organic Frameworks. J. Am. Chem.
Soc..

[9] Ponomareva V. G., Kovalenko K. A., Chupakhin A. P., Dybtsev D. N., Shutova E. S., Fedin V. P. (2012). Imparting
High Proton Conductivity to a Metal–Organic
Framework Material by Controlled Acid Impregnation. J. Am. Chem. Soc..

[10] Taylor J.
M., Mah R. K., Moudrakovski I. L., Ratcliffe C. I., Vaidhyanathan R., Shimizu G. K. H. (2010). Facile Proton Conduction via Ordered
Water Molecules in a Phosphonate Metal–Organic Framework. J. Am. Chem. Soc..

[11] Chen X., Wang S.-Z., Xiao S.-H., Li Z.-F., Li G. (2022). High Protonic
Conductivity of Three Highly Stable Nanoscale Hafnium­(IV) Metal–Organic
Frameworks and Their Imidazole-Loaded Products. Inorg. Chem..

[12] Xie X.-X., Yang Y.-C., Dou B.-H., Li Z.-F., Li G. (2020). Proton Conductive
Carboxylate-Based Metal–Organic Frameworks. Coord. Chem. Rev..

[13] Cavka J. H., Jakobsen S., Olsbye U., Guillou N., Lamberti C., Bordiga S., Lillerud K. P. (2008). A New Zirconium
Inorganic Building
Brick Forming Metal Organic Frameworks with Exceptional Stability. J. Am. Chem. Soc..

[14] Bai Y., Dou Y., Xie L.-H., Rutledge W., Li J.-R., Zhou H.-C. (2016). Zr-Based
Metal–Organic Frameworks: Design, Synthesis, Structure, and
Applications. Chem. Soc. Rev..

[15] Chen X., Li G. (2020). Proton Conductive Zr-Based MOFs. Inorg. Chem.
Front..

[16] Taylor J. M., Dekura S., Ikeda R., Kitagawa H. (2015). Defect Control To Enhance
Proton Conductivity in a Metal–Organic Framework. Chem. Mater..

[17] Phang W. J., Jo H., Lee W. R., Song J. H., Yoo K., Kim B., Hong C. S. (2015). Superprotonic
Conductivity of a UiO-66 Framework Functionalized
with Sulfonic Acid Groups by Facile Postsynthetic Oxidation. Angew. Chem., Int. Ed..

[18] Yang F., Huang H., Wang X., Li F., Gong Y., Zhong C., Li J.-R. (2015). Proton Conductivities in Functionalized
UiO-66: Tuned Properties, Thermogravimetry Mass, and Molecular Simulation
Analyses. Cryst. Growth Des..

[19] Donnadio A., Narducci R., Casciola M., Marmottini F., D’Amato R., Jazestani M., Chiniforoshan H., Costantino F. (2017). Mixed Membrane Matrices Based on Nafion/UiO-66/SO_3_H-UiO-66 Nano-MOFs: Revealing the Effect of Crystal Size,
Sulfonation, and Filler Loading on the Mechanical and Conductivity
Properties. ACS Appl. Mater. Interfaces.

[20] Liu S., Yue Z., Liu Y. (2015). Incorporation of Imidazole within
the Metal–Organic
Framework UiO-67 for Enhanced Anhydrous Proton Conductivity. Dalton Trans..

[21] Mikhailova D., Karakulina O. M., Batuk D., Hadermann J., Abakumov A. M., Herklotz M., Tsirlin A. A., Oswald S., Giebeler L., Schmidt M., Eckert J. (2016). Layered-to-Tunnel Structure
Transformation and Oxygen Redox Chemistry in LiRhO_2_ upon
Li Extraction and Insertion. Inorg. Chem..

[22] Luo H.-B., Ren Q., Wang P., Zhang J., Wang L., Ren X.-M. (2019). High Proton
Conductivity Achieved by Encapsulation of Imidazole Molecules into
Proton-Conducting MOF-808. ACS Appl. Mater.
Interfaces.

[23] Sharma A., Lim J., Jeong S., Won S., Seong J., Lee S., Kim Y. S., Baek S. B., Lah M. S. (2021). Superprotonic Conductivity
of MOF-808 Achieved by Controlling the Binding Mode of Grafted Sulfamate. Angew. Chem., Int. Ed..

[24] Yang F., Shi R., Huang H., Zhang Z., Guo X., Qiao Z., Zhong C. (2022). Nanochannel Engineering in Metal–Organic Frameworks by Grafting
Sulfonic Groups for Boosting Proton Conductivity. ACS Appl. Energy Mater..

[25] Howarth A. J., Liu Y., Li P., Li Z., Wang T. C., Hupp J. T., Farha O. K. (2016). Chemical, Thermal and Mechanical Stabilities of Metal–Organic
Frameworks. Nat. Rev. Mater..

[26] Bůžek D., Demel J., Lang K. (2018). Zirconium Metal–Organic Framework
UiO-66: Stability in an Aqueous Environment and Its Relevance for
Organophosphate Degradation. Inorg. Chem..

[27] Bůžek D., Adamec S., Lang K., Demel J. (2021). Metal–Organic
Frameworks vs. Buffers: Case Study of UiO-66 Stability. Inorg. Chem. Front..

[28] Bůžek D., Hynek J., Kloda M., Zlámalová V., Bezdička P., Adamec S., Lang K., Demel J. (2024). Zirconium-Based
Metal–Organic Frameworks: The Relation between Linker Connectivity,
Structure Stability, and Catalytic Activity towards Organophosphates. Inorg. Chem. Front..

[29] Mondloch J.
E., Katz M. J., Planas N., Semrouni D., Gagliardi L., Hupp J. T., Farha O. K. (2014). Are Zr_6_-Based MOFs Water
Stable? Linker Hydrolysis vs. Capillary-Force-Driven Channel Collapse. Chem. Commun..

[30] Hynek J., Ondrušová S., Bůžek D., Kovář P., Rathouský J., Demel J. (2017). Postsynthetic Modification
of a Zirconium Metal–Organic Framework at the Inorganic Secondary
Building Unit with Diphenylphosphinic Acid for Increased Photosensitizing
Properties and Stability. Chem. Commun..

[31] Yang X., Li Q.-X., Chi S.-Y., Li H.-F., Huang Y.-B., Cao R. (2022). Hydrophobic perfluoroalkane modified metal-organic frameworks for
the enhanced electrocatalytic reduction of CO_2_. SmartMat.

[32] Yang J., Liu S., Sun H., Chen D. (2025). One-Pot Synthesis of Hydrophobic
Porphyrin Zirconium-Based MOFs for the Photoreduction of CO_2_ to Formate. Inorg. Chem..

[33] Feng D., Gu Z.-Y., Li J.-R., Jiang H.-L., Wei Z., Zhou H.-C. (2012). Zirconium-Metalloporphyrin PCN-222: Mesoporous Metal–Organic
Frameworks with Ultrahigh Stability as Biomimetic Catalysts. Angew. Chem., Int. Ed..

[34] Morris W., Volosskiy B., Demir S., Gándara F., McGrier P. L., Furukawa H., Cascio D., Stoddart J. F., Yaghi O. M. (2012). Synthesis, Structure, and Metalation of Two New Highly
Porous Zirconium Metal–Organic Frameworks. Inorg. Chem..

[35] Feng D., Chung W.-C., Wei Z., Gu Z.-Y., Jiang H.-L., Chen Y.-P., Darensbourg D. J., Zhou H.-C. (2013). Construction of
Ultrastable Porphyrin Zr Metal–Organic Frameworks through Linker
Elimination. J. Am. Chem. Soc..

[36] Kim S., Hong I. (2008). Effects of Humidity and Temperature on a Proton Exchange Membrane
Fuel Cell (PEMFC) Stack. J. Ind. Eng. Chem..

[37] Kloda M., Ondrušová S., Lang K., Demel J. (2021). Phosphinic
Acids as Building Units in Materials Chemistry. Coord. Chem. Rev..

[38] Yeum, B. ZSimpWin; EChem. Software; Ann Arbor: MI, 1999–2013.

[39] Barsoukov, E. ; Macdonald, J. R. Impedance Spectroscopy, 2nd ed.; John Wiley & Sons Inc.: Hoboken, NJ, 2018.

[40] Accelrys Software Inc. Materials Studio Modeling Environment, Release 4.3 Documentation; Accelrys Software Inc.: San Diego, CA, 2003.

[41] Zee, D. Z. ; Harris, T. D. CCDC 1992909: Experimental Crystal Structure Determination; Cambridge Crystallographic Data Centre, 2020.

[42] Rappe A. K., Casewit C. J., Colwell K. S., Goddard W. A., Skiff W. M. (1992). UFF, a full periodic table force
field for molecular
mechanics and molecular dynamics simulations. J. Am. Chem. Soc..

[43] Sun H. (1998). COMPASS: An
ab Initio Force-Field Optimized for Condensed-Phase Applications—Overview
with Details on Alkane and Benzene Compounds. J. Phys. Chem. B.

[44] Rappé A. K., Goddard III W. A. (1991). Charge
Equilibration for Molecular Dynamics Simulations. J. Phys. Chem..

[45] Wells A., Chaffee A. L. (2015). Ewald Summation
for Molecular Simulations. J. Chem. Theory Comput..

[46] Feng D., Jiang H.-L., Chen Y.-P., Gu Z.-Y., Wei Z., Zhou H. C. (2013). Metal–Organic Frameworks Based on Previously
Unknown Zr_8_/Hf_8_ Cubic Clusters. Inorg. Chem..

[47] Koschnick C., Stäglich R., Scholz T., Terban M. W., von Mankowski A., Savasci G., Binder F., Schökel A., Etter M., Nuss J., Siegel R., Germann L. S., Ochsenfeld C., Dinnebier R. E., Senker J., Lotsch B. V. (2021). Understanding
Disorder and Linker Deficiency in Porphyrinic Zirconium-Based Metal–Organic
Frameworks by Resolving the Zr_8_O_6_ Cluster Conundrum
in PCN-221. Nat. Commun..

[48] Hnatejko Z., Lis S., Stryła Z. (2010). Preparation and Characterization of Uranyl Complexes
with Phosphonate Ligands. J. Therm. Anal. Calorim..

[49] Datar A., Yoon S., Lin L.-C., Chung Y. G. (2022). Brunauer–Emmett–Teller
Areas from Nitrogen and Argon Isotherms Are Similar. Langmuir.

[50] Smith K., Foglia F., Clancy A. J., Brett D. J. L., Miller T. S. (2023). Nafion
Matrix and Ionic Domain Tuning for High-Performance Composite Proton
Exchange Membranes. Adv. Funct. Mater..

[51] Romero-Muñiz I., Romero-Muñiz C., del Castillo-Velilla I., Marini C., Calero S., Zamora F., Platero-Prats A. E. (2022). Revisiting
Vibrational Spectroscopy to Tackle the Chemistry of Zr_6_O_8_ Metal–Organic Framework Nodes. ACS Appl. Mater. Interfaces.

[52] Valenzano L., Civalleri B., Chavan S., Bordiga S., Nilsen M. H., Jakobsen S., Lillerud K. P., Lamberti C. (2011). Disclosing the Complex
Structure of UiO-66 Metal Organic Framework: A Synergic Combination
of Experiment and Theory. Chem. Mater..

[53] Socrates, G. Infrared and Raman Characteristic Group Frequencies: Tables and Charts, 3rd ed.; John Wiley & Sons: Chichester, 2001.

[54] Chen X., Lyu Y., Wang Z., Qiao X., Gates B. C., Yang D. (2020). Tuning Zr_12_O_22_ Node Defects as Catalytic Sites in the Metal–Organic
Framework Hcp UiO-66. ACS Catal..

[55] Babucci M., Hoffman A. S., Bare S. R., Gates B. C. (2021). Characterization
of a Metal–Organic Framework Zr_6_O_8_ Node-Supported
Atomically Dispersed Iridium Catalyst for Ethylene Hydrogenation by
X-Ray Absorption Near-Edge Structure and Infrared Spectroscopies. J. Phys. Chem. C.

[56] Wei R., Gaggioli C. A., Li G., Islamoglu T., Zhang Z., Yu P., Farha O. K., Cramer C. J., Gagliardi L., Yang D., Gates B. C. (2019). Tuning
the Properties
of Zr_6_O_8_ Nodes in the Metal–Organic Framework
UiO-66 by Selection of Node-Bound Ligands and Linkers. Chem. Mater..

[57] Aydin M. (2014). Comparative
Study of the Structural and Vibroelectronic Properties of Porphyrin
and Its Derivatives. Molecules.

[58] Aydin M. (2013). DFT and Raman
Spectroscopy of Porphyrin Derivatives: Tetraphenylporphine (TPP). Vib. Spectrosc..

[59] Shearer G. C., Chavan S., Ethiraj J., Vitillo J. G., Svelle S., Olsbye U., Lamberti C., Bordiga S., Lillerud K. P. (2014). Tuned to
Perfection: Ironing out the Defects in Metal–Organic Framework
UiO-66. Chem. Mater..

[60] Meng X., Wang H.-N., Wang L.-S., Zou Y.-H., Zhou Z.-Y. (2019). Enhanced
Proton Conductivity of a MOF-808 Framework through Anchoring Organic
Acids to the Zirconium Clusters by Post-Synthetic Modification. CrystEngComm.

[61] Luo H., Wang M., Liu S., Xue C., Tian Z., Zou Y., Ren X. (2017). Proton Conductance
of a Superior Water-Stable Metal-Organic
Framework and Its Composite Membrane with Poly­(vinylidene fluoride). Inorg. Chem..

