# Supplementary material for: Effect of Elevated Air Humidity on the Structure and Proton Conductivity of Porphyrin-Based Zr(IV)-MOFs
Source: Inorg Chem. 2025 Jul 29;64(31):15993–6004. doi: 10.1021/acs.inorgchem.5c02165 (PMC12344768; doi:10.1021/acs.inorgchem.5c02165)
Supplement: Supplementary file 1 [file ic5c02165_si_001.pdf]

Supporting information

Effect of elevated air humidity on the structure and proton conductivity of porphyrin-based Zr(IV)-MOFs

Jan Hynek,[a] Matouš Kloda,[a] Miroslava Litecká,[a] Anna Vykydalová,[a,b] Jakub Tolasz,[a] Miroslav Pospíšil,[c] Zuzana Morávková,[d] Mandeep K. Chahal,[e] Ludvík Beneš,[f] Tomáš Plecháček,[f] Klára Melánová,[f]*

[a] Institute of Inorganic Chemistry of the Czech Academy of Sciences, Husinec-Řež 1001, 25068 Řež, Czech Republic

[b] Polymer Institute, Slovak Academy of Sciences, Dúbravská Cesta 9, 84541, Bratislava, Slovakia

[c] Department of Chemical Physics and Optics, Faculty of Mathematics and Physics, Charles University, Ke Karlovu 3, Prague 12116, Czech Republic

[d] Institute of Macromolecular Chemistry of the Czech Academy of Sciences, Heyrovského nám. 2, Prague, 162 06, Czech Republic

[e] School of Chemistry and Forensic Science, University of Kent, Canterbury, CT2 7NH, United Kingdom

[f] Center of Materials and Nanotechnologies, Faculty of Chemical Technology, University of Pardubice, Studentská 95, 53210 Pardubice, Czech Republic

* **Corresponding author email**: hynek@iic.cas.cz

Table of contents

Table of contents 2

Materials 2

Instrumental methods 2

Characterization of the materials 4

Powder XRD 4

Elemental analysis 6

Infrared spectra 7

Thermogravimetric analysis 9

Molecular dynamics calculation 17

Adsorption properties 18

Stability at defined air humidity 22

Evaluation of the structural changes by infrared and Raman spectroscopy 38

Scanning electron microscopy images 51

Proton conductivity 53

Literature 68

Materials

5,10,15,20-tetrakis(4-carboxyphenyl)porphyrin (PorphyChem, France), zirconium chloride, zirconyl chloride octahydrate, imidazole (all Sigma-Aldrich), formic acid, benzoic acid (both Lach-Ner, Czech Republic), diphenylphosphinic acid (DPPA, BLDpharm), acetone (VWR Chemicals) and dimethylformamide (DMF, Penta, Czech Republic) were used as purchased.

Instrumental methods

Powder X-ray diffraction (XRD) of the samples was measured using a PANalytical X'Pert PRO diffractometer in a transmission setup equipped with a conventional Cu X-ray tube (40 kV, 30 mA) or Bruker D8 ADVANCE.DAVINCI (Bruker AXS, Germany) diffractometer with Bragg-Brentano θ-θ goniometer equipped with a LynxEye XE-T detector and Cu X-ray tube (40 kV, 30 mA). The diffraction angles were measured at room temperature from 2 to 20° (2q) in 0.01° steps with a counting time of 0.45 s per step. Qualitative analysis was performed with the HighScorePlus software package (PANalytical, Almelo, The Netherlands, version 3.0).

Thermal analyses (DTA/TGA) were carried out on a Setaram SETSYS Evolution-16-MS (Setaram, Caluire, France) instrument coupled with a mass spectrometer. The measurements were performed in argon (60 mL min^{-1}) from 25 to 800 °C with a heating rate of 5 °C min^{-1}.

Fourier transform infrared spectra (FTIR) in region 4000–400 cm^{-1} were recorded using a Thermo Nicolet NEXUS 870 FTIR Spectrometer (DTGS TEC detector; 64 scans; resolution 2 cm^{-1}) in transmission mode in potassium bromide pellets; as well as using a GoldenGate ATR accessory (MCT/A detector; 256 scans; resolution 2 cm^{-1}). The spectra were corrected for the carbon dioxide and humidity in the optical path.

Raman spectra were collected on a Renishaw inVia Reflex Raman spectrometer (Leica DM LM microscope; objective magnification x50) with a He-Ne 633 nm laser (holographic grating 1800 lines mm^{-1}); and on a Renishaw inVia Qontor Raman microspectrometer (Leica DM LM microscope; objective magnification x50) with a sapphire 488 nm (holographic grating with 2400 lines mm^{-1}). Spectra were obtained on at least 20 spots on the sample to ensure the sample was homogeneous – average spectra of each detected form are presented.

The content of C, H and N was determined by a standard combustion technique (Thermo Scientific FlashSmartTM 2000 Elemental analyzer). The content of P and Zr was measured by ICP-MS (Agilent 7900 equipped with an Ar burner, ORS 4 collision cell and orthogonal hyperbolic quadrupole mass analyser), 20 ppb indium solution was used as an internal standard. Prior the measurement, the samples were dissolved in the mixture of acids (12 mL of HCl, 4 mL of HNO_3, and 4 mL HF for 10 mg of sample) under microwave irradiation.

Adsorption isotherms of Ar were recorded using a 3P micro 300 instrument (3P Instruments) equipped with cryoTune at the boiling point of Ar. Before measurement, the samples were degassed at 100 °C for 24 h under dynamic vacuum and then activated again at 100 °C for 1 h. The BET surface area was calculated from 0.005 – 0.1 p/p_0 range as provided by the 3P Instrument software. The external surface area was calculated by t-plot from 0.4 – 0.9 p/p_0 range. Adsorption of water vapor was measured using a Belsorp maxII instrument (Microtrac MRB). The measurements were carried out at 298 K. Before the measurement, the sample was degassed at 100 °C for 16 h under dynamic vacuum.

The high-resolution scanning electron microscopy images were taken by FEI NovaNanoSEM 450 instrument with an additional CBS detector for backscattered electrons and an accelerating voltage of 5kV was used for the measurement. To increase the signal-to-noise ratio, an overvoltage of 2000V was applied as stage bias. A pinch of each powdered sample was dispersed in 1 mL of acetone in a glass vial by 10 min of ultrasonication. After that 10 µL of the suspension was dropped onto a silicon wafer and the samples were dried in a vacuum chamber.

Characterization of the materials

Powder XRD

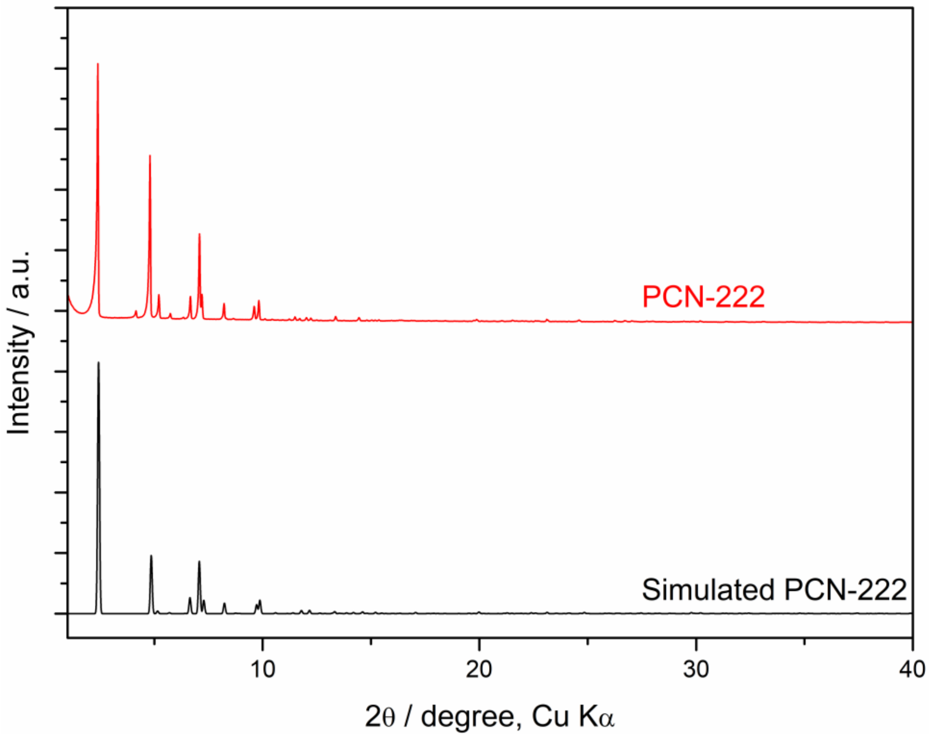

Figure S1: Comparison of measured (top) and simulated (bottom) powder XRD pattern of PCN-222.

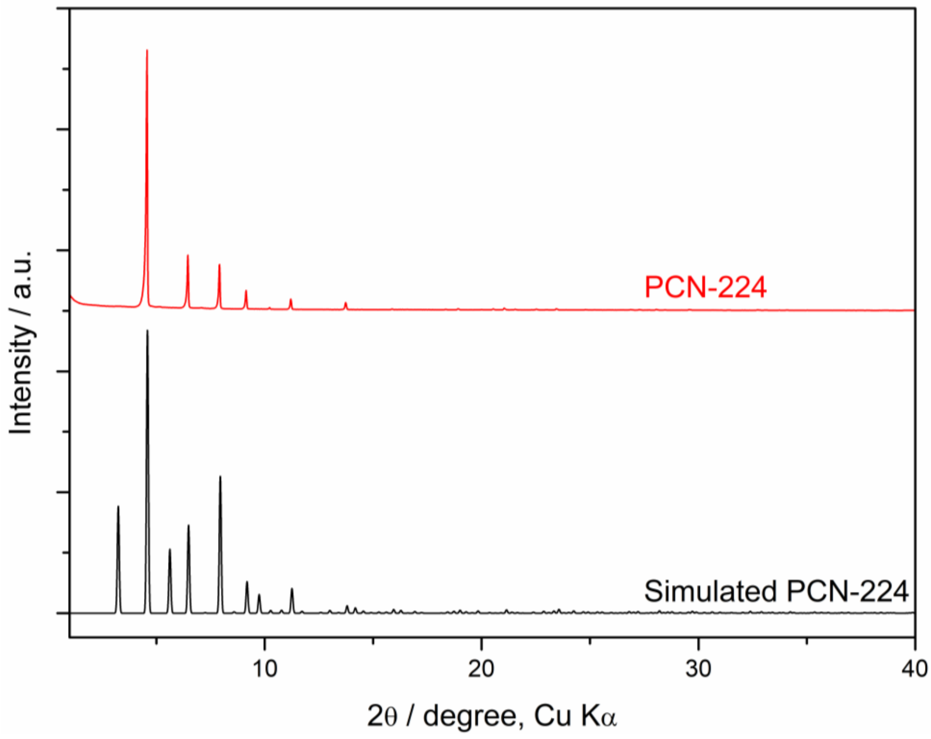

Figure S2: Comparison of measured (top) and simulated (bottom) powder XRD pattern of PCN-224.

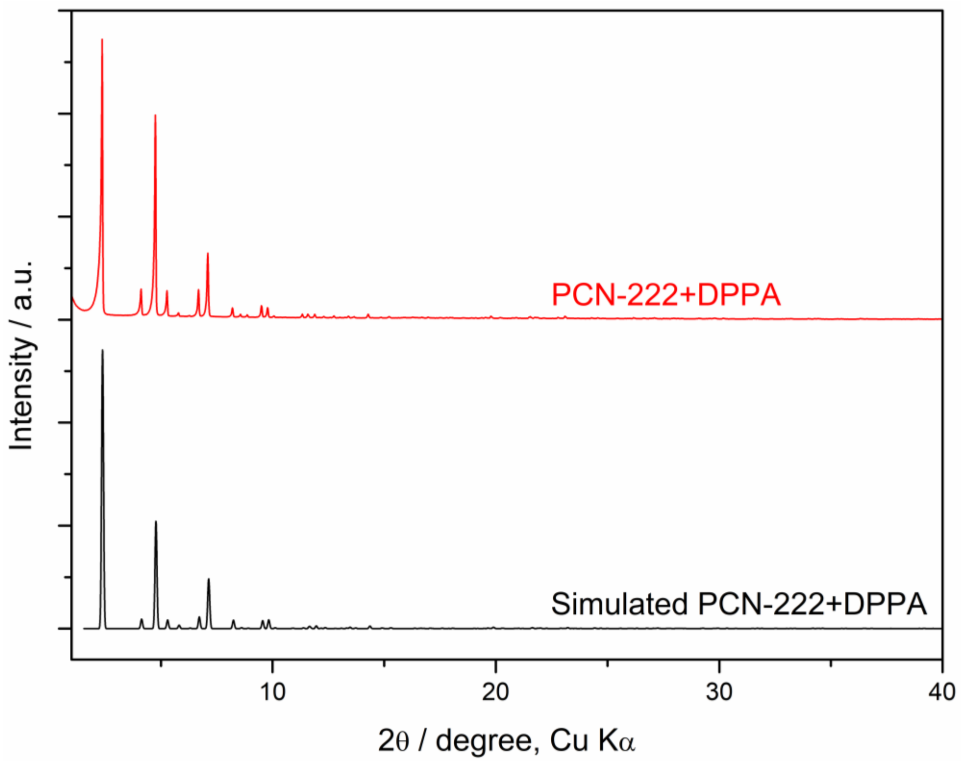

Figure S3: Comparison of measured (top) and simulated (bottom) powder XRD pattern of PCN-222+DPPA.

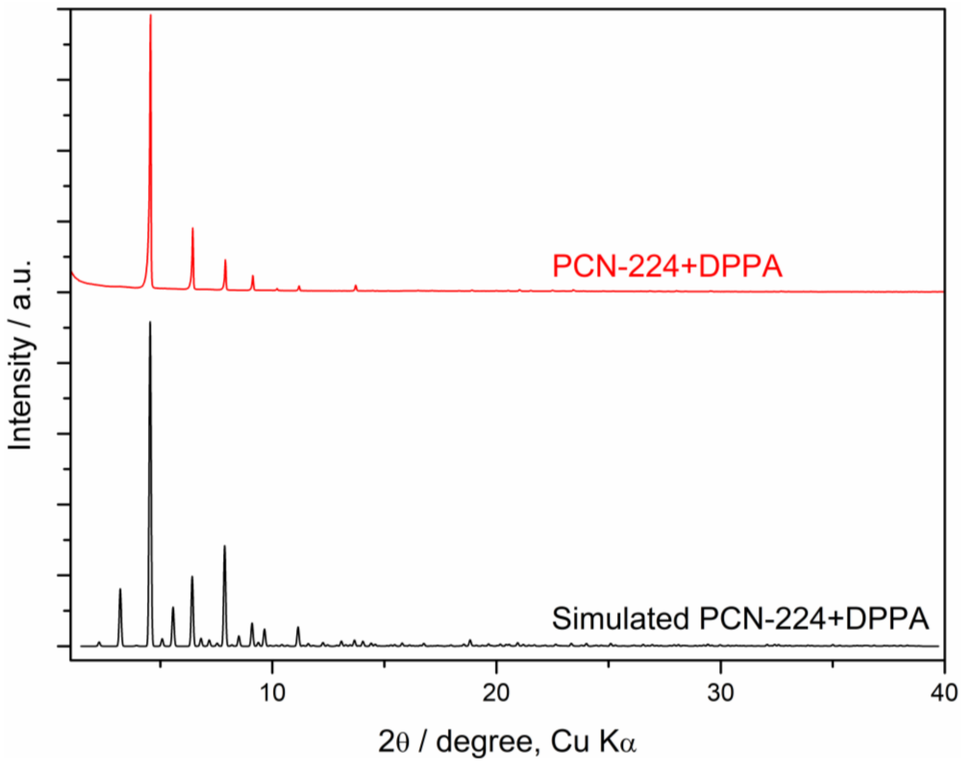

Figure S4: Comparison of measured (top) and simulated (bottom) powder XRD pattern of PCN-224+DPPA.

Elemental analysis

Table S1: Elemental composition of the studied MOFs in weight % determined by CHN analysis and ICP-MS. The values of P / Zr_6 ratio were calculated based on the atomic content of the respective elements. The imidazole / TCPP ratio was calculated based on the difference in the content of N in samples before and after the adsorption of imidazole.

Sample	C	H	N	P	Zr	P / Zr_6 ratio	Imidazole / TCPP ratio
PCN-222	46.58	2.47	4.73	-	20.3	-	-
Im@PCN-222	47.38	2.90	8.27	-	17.9	-	1.87
PCN-222+DPPA	52.74	3.02	3.97	1.78	16.7	1.88	-
Im@PCN-222+DPPA	52.61	3.15	6.67	1.80	16.2	1.96	1.62
PCN-224	53.51	2.97	4.23	-	17.6	-	-
Im@PCN-224	52.13	3.06	6.58	-	16.4	-	1.32
PCN-224+DPPA	54.18	3.12	3.72	2.24	15.3	2.59	-
Im@PCN-224+DPPA	56.24	3.29	6.23	2.23	15.0	2.63	1.59

Infrared spectra

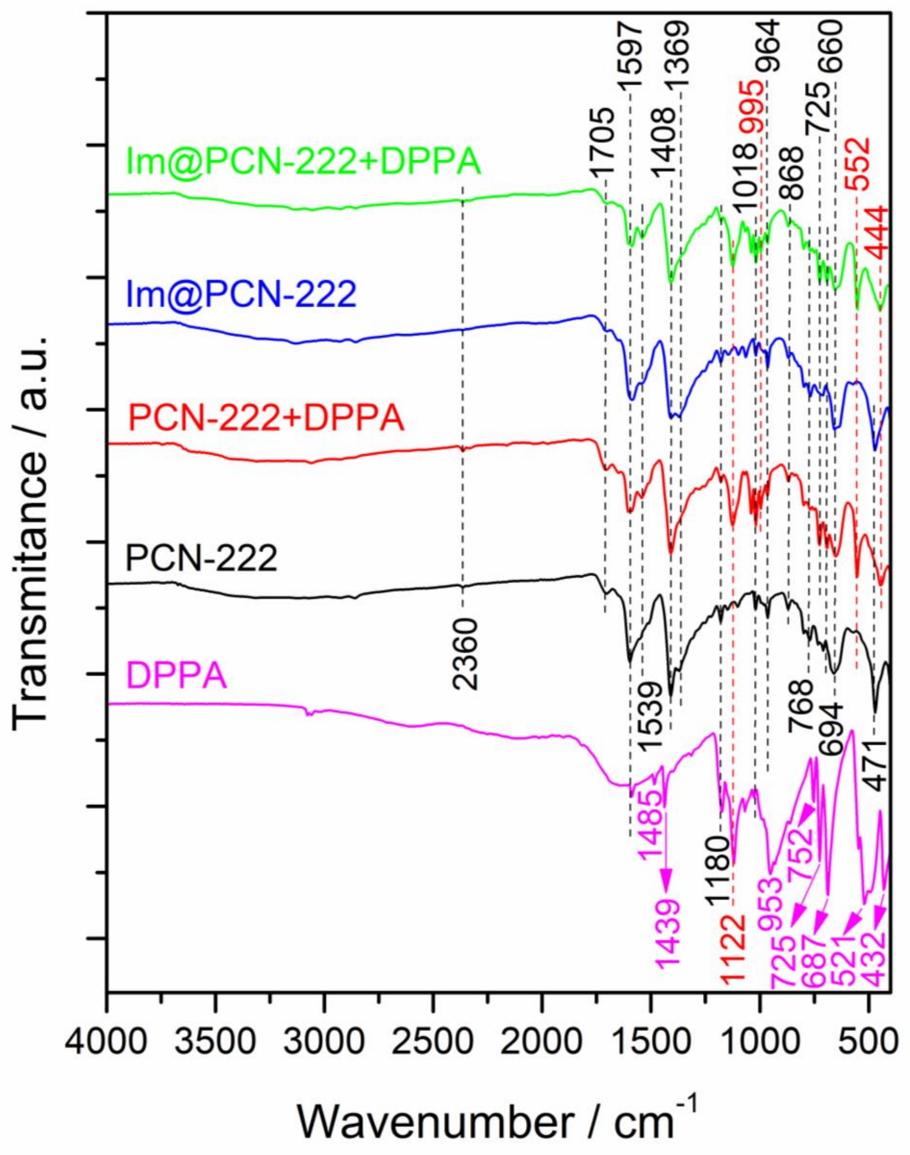

Figure S5: Infrared spectra of the PCN-222 samples and DPPA. Signals specific for DPPA-modified materials are marked red. The spectra are shifted vertically to avoid overlaps.

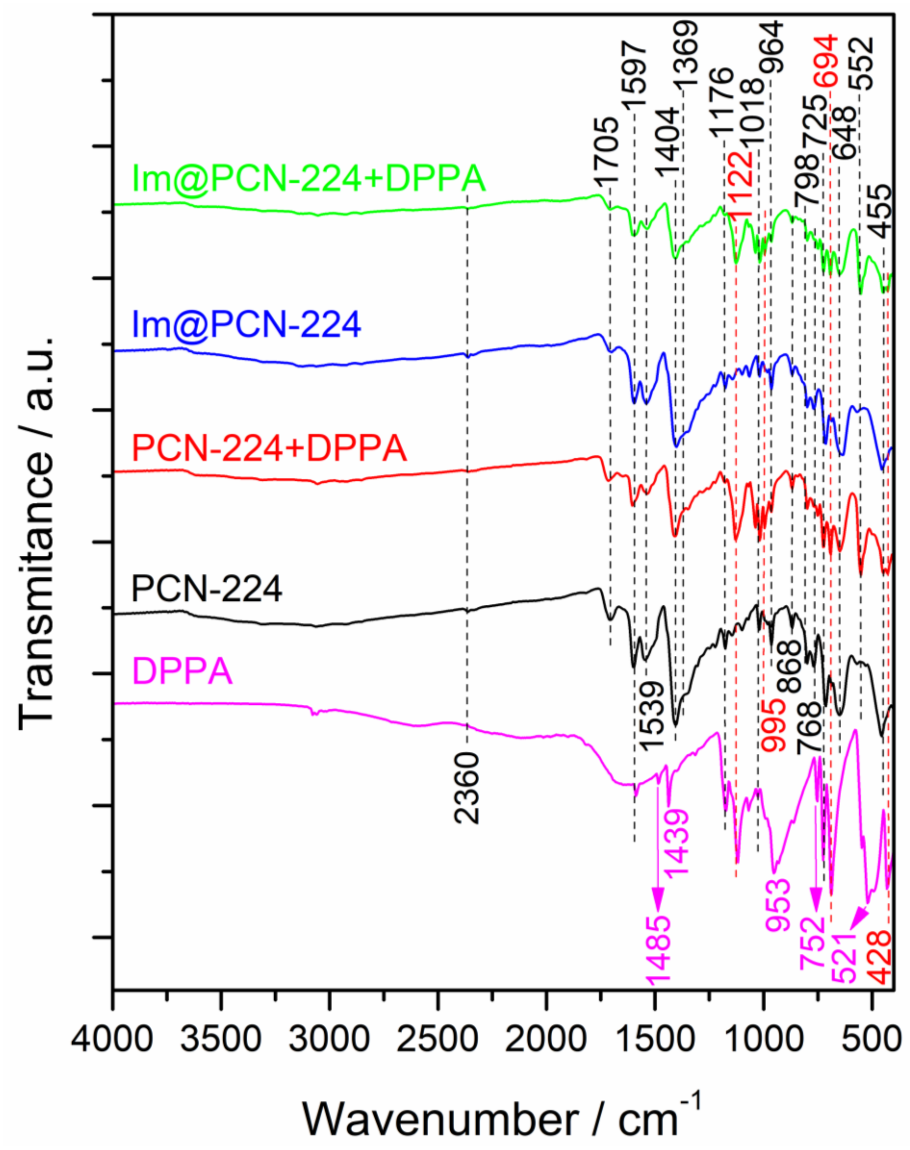

Figure S6: Infrared spectra of the PCN-224 samples and DPPA. Signals specific for DPPA-modified materials are marked red. The spectra are shifted vertically to avoid overlaps.

Thermogravimetric analysis

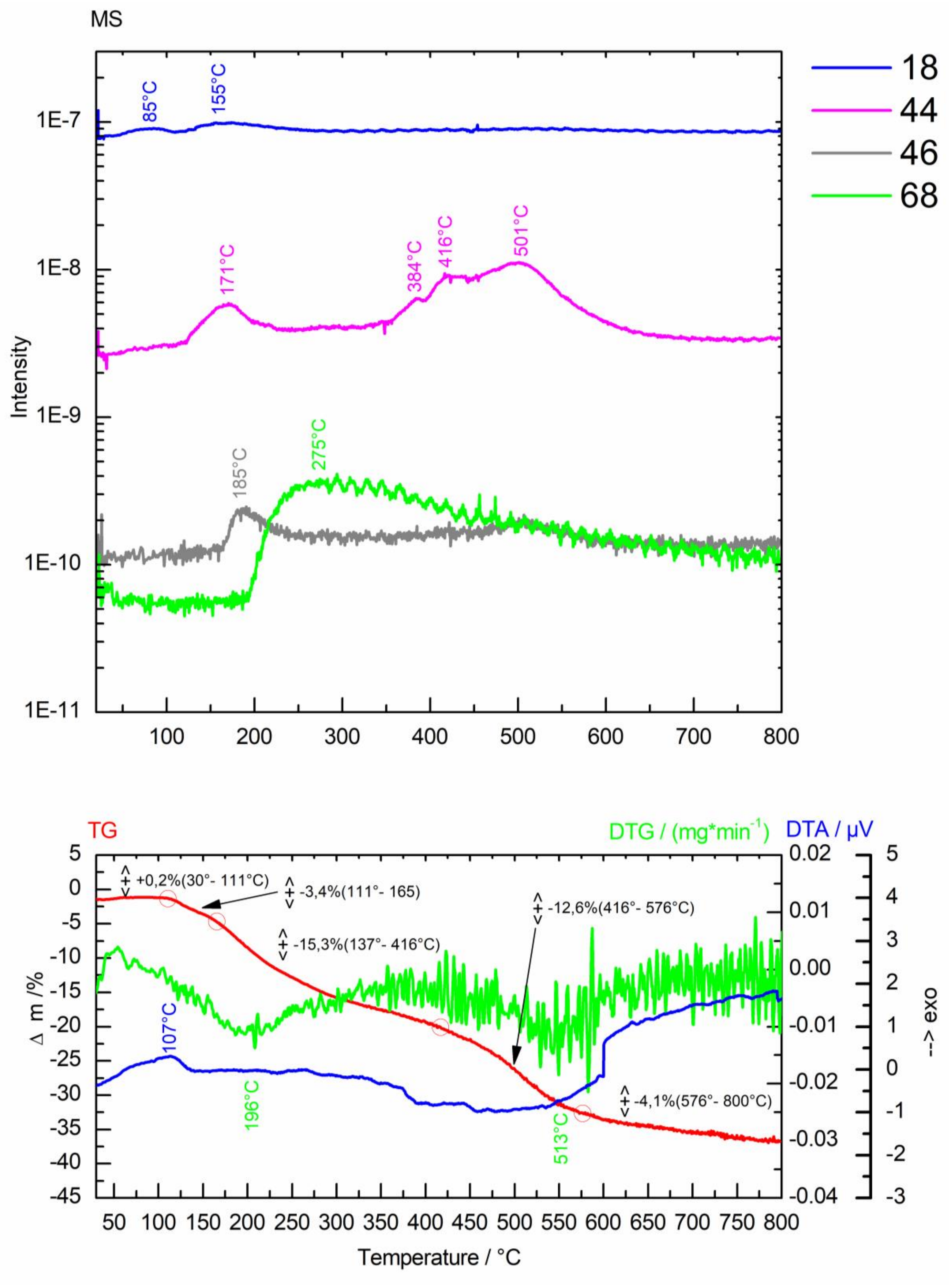

Figure S7: TGA/DTA curves (bottom) and the evolution of gases (top) for PCN-222 in Ar; m/z = 18 – H_2O, m/z = 44 – CO_2, m/z = 46 – HCOOH, and m/z = 68 – imidazole.

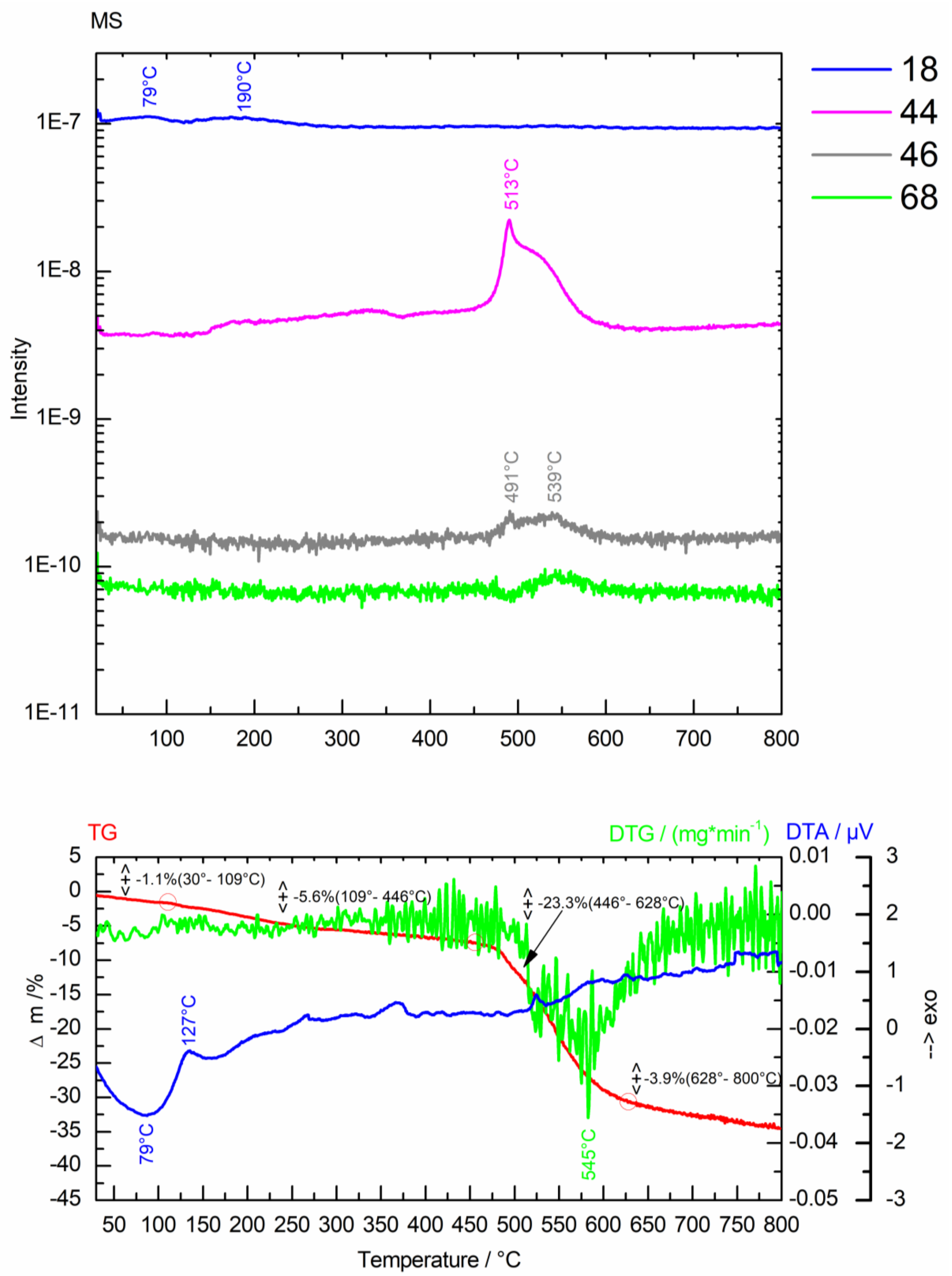

Figure S8: TGA/DTA curves (bottom) and the evolution of gases (top) for PCN-222+DPPA in Ar; m/z = 18 – H_2O, m/z = 44 – CO_2, m/z = 46 – HCOOH, and m/z = 68 – imidazole.

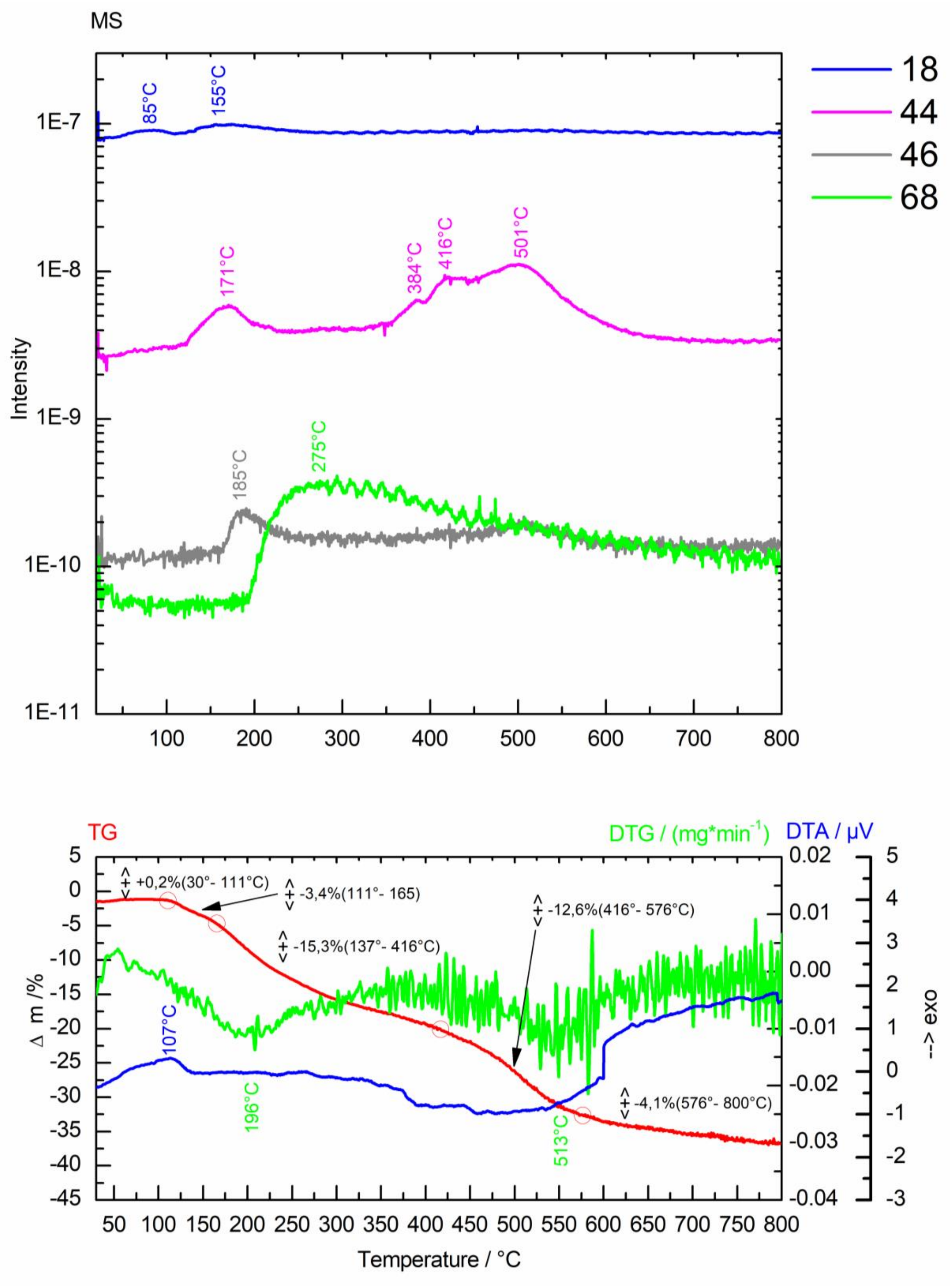

Figure S9: TGA/DTA curves (bottom) and the evolution of gases (top) for Im@PCN-222 in Ar; m/z = 18 – H_2O, m/z = 44 – CO_2, m/z = 46 – HCOOH, and m/z = 68 – imidazole.

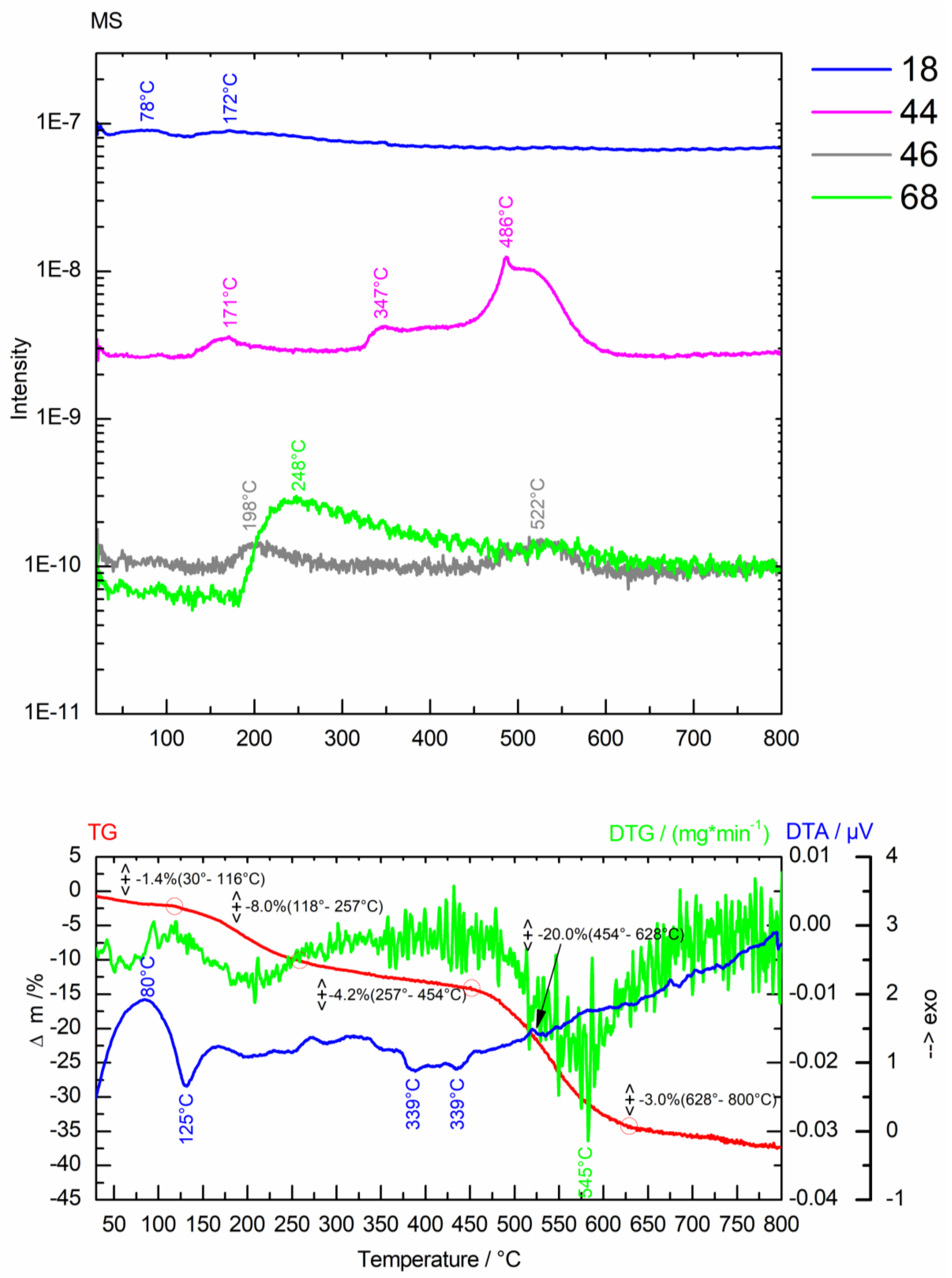

Figure S10: TGA/DTA curves (bottom) and the evolution of gases (top) for Im@PCN-222+DPPA in Ar; m/z = 18 – H_2O, m/z = 44 – CO_2, m/z = 46 – HCOOH, and m/z = 68 – imidazole.

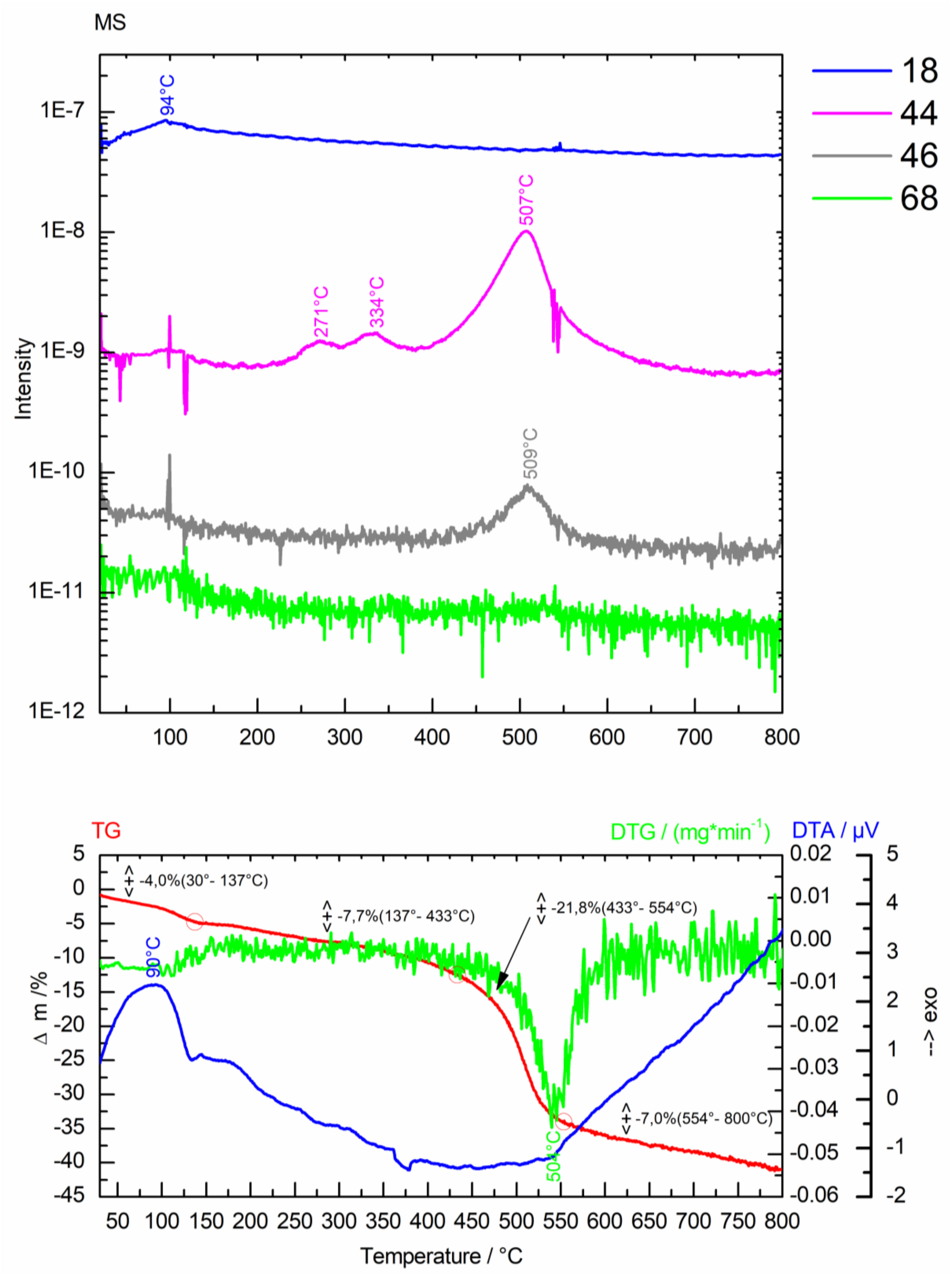

Figure S11: TGA/DTA curves (bottom) and the evolution of gases (top) for PCN-224 in Ar; m/z = 18 – H_2O, m/z = 44 – CO_2, m/z = 46 – HCOOH, and m/z = 68 – imidazole.

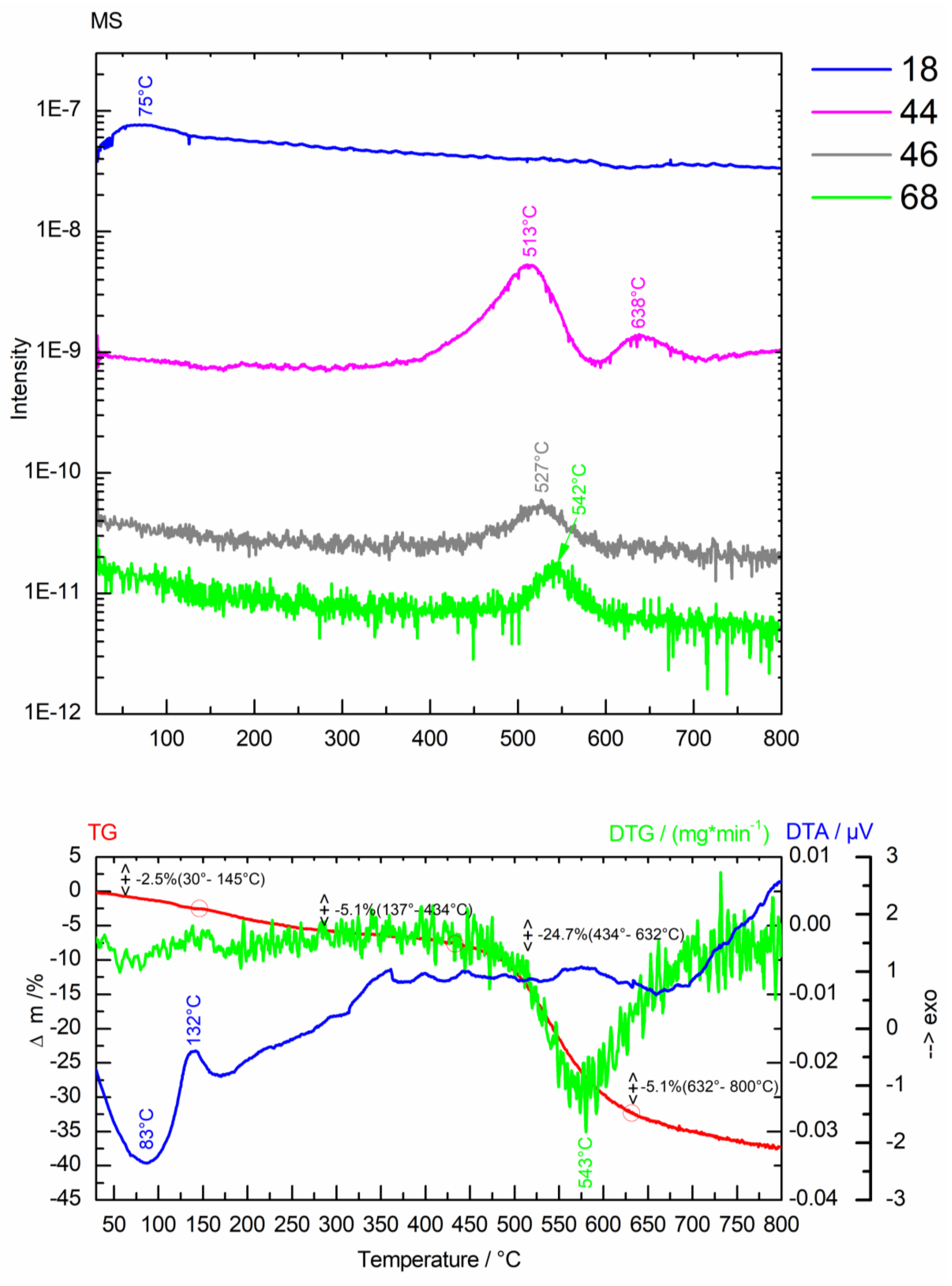

Figure S12: TGA/DTA curves (bottom) and the evolution of gases (top) for PCN-224+DPPA in Ar; m/z = 18 – H_2O, m/z = 44 – CO_2, m/z = 46 – HCOOH, and m/z = 68 – imidazole.

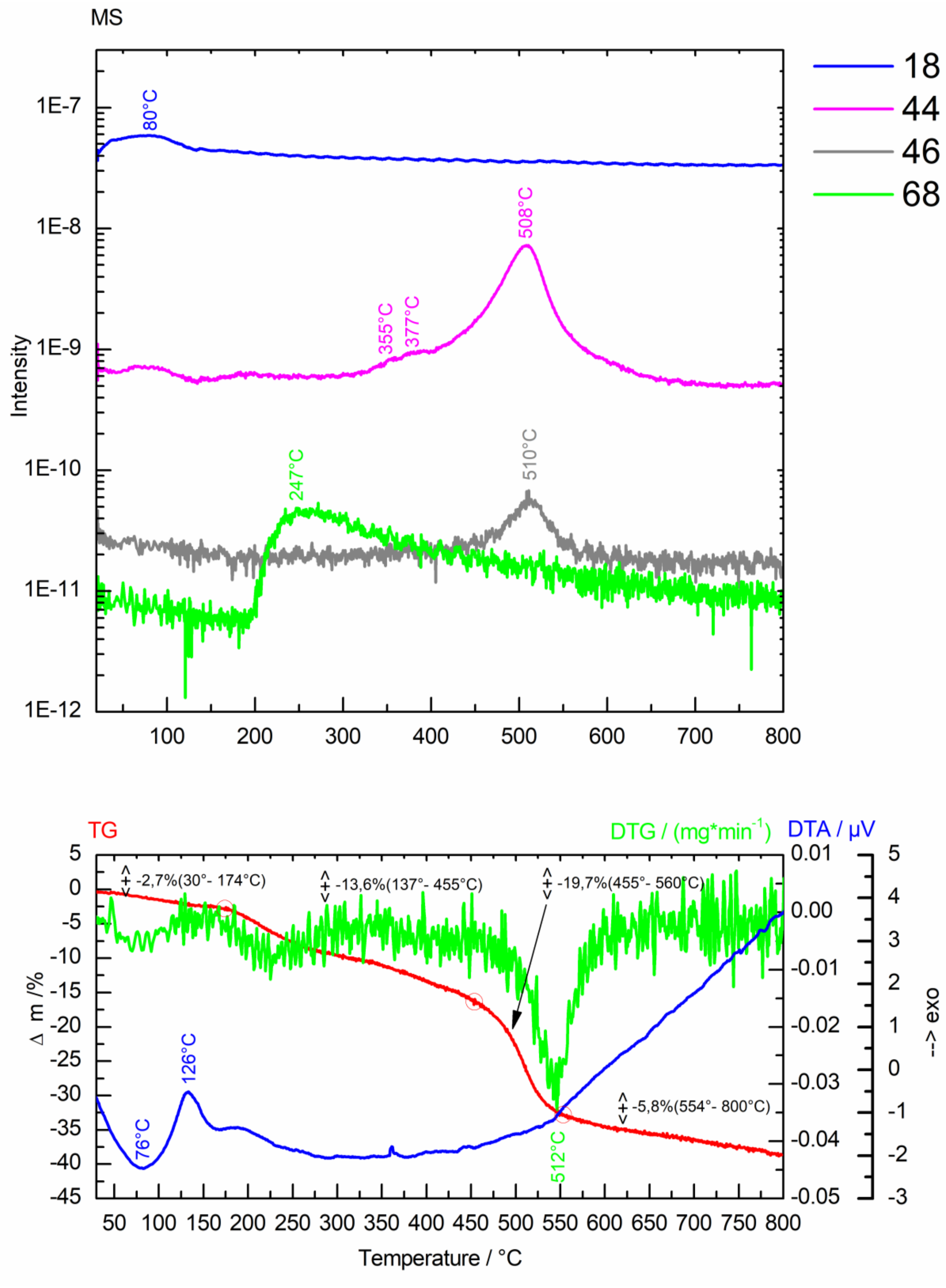

Figure S13: TGA/DTA curves (bottom) and the evolution of gases (top) for Im@PCN-224 in Ar; m/z = 18 – H_2O, m/z = 44 – CO_2, m/z = 46 – HCOOH, and m/z = 68 – imidazole.

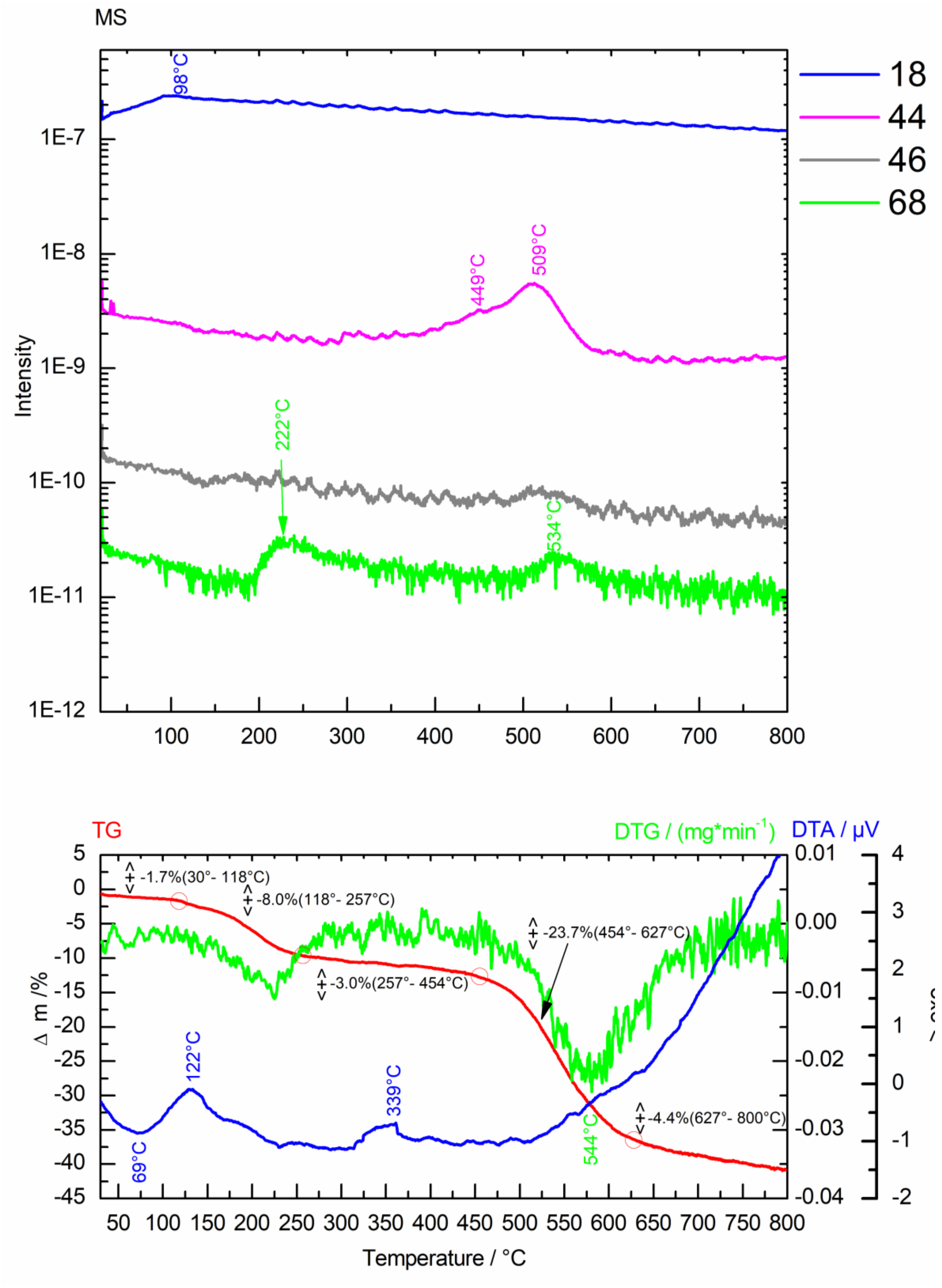

Figure S14: TGA/DTA curves (bottom) and the evolution of gases (top) for Im@PCN-224+DPPA in Ar; m/z = 18 – H_2O, m/z = 44 – CO_2, m/z = 46 – HCOOH, and m/z = 68 – imidazole.

Molecular dynamics calculation

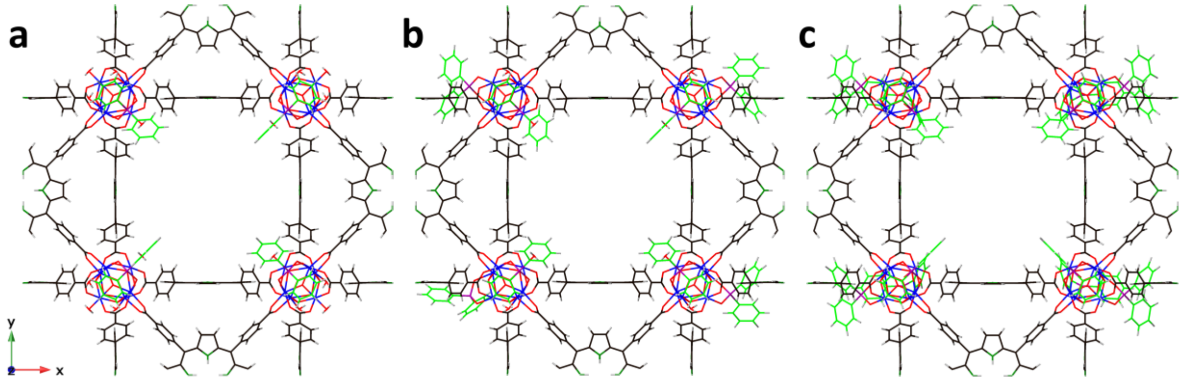

Figure S15. Structural model of PCN-224+DPPA with DPPA molecules bound in the bridging coordination mode containing (a) 1, (b) 2, and (c) 3 DPPA molecules per one SBU. The bonded DPPA molecules are depicted in green color for better clarity.

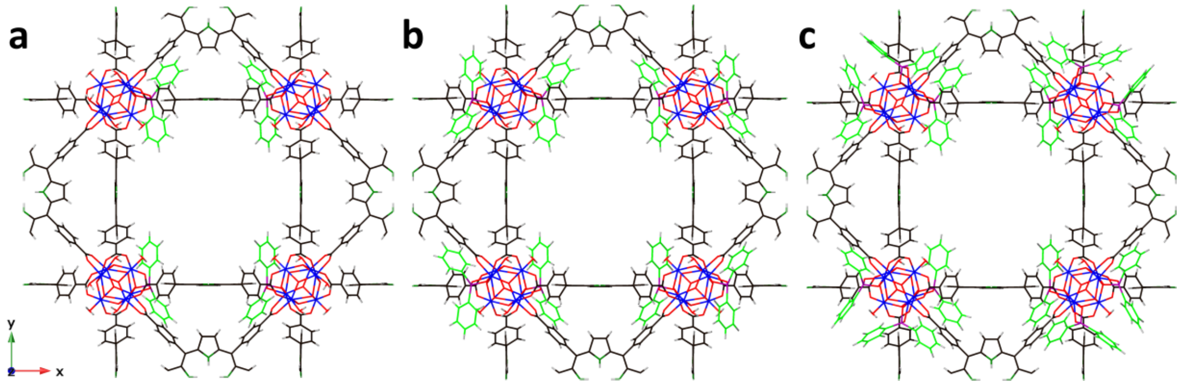

Figure S16. Structural model of PCN-224+DPPA with DPPA molecules bound in the chelating coordination mode containing (a) 1, (b) 2, and (c) 3 DPPA molecules per one SBU. The bonded DPPA molecules are depicted in green color for better clarity.

Table S2. Total energy values of the first, second and third DPPA molecule on an SBU of PCN-224 bound in either bridging or chelating coordination mode.

Coordination mode		E_{total} / kcal·mol^{-1}	$E_{non\text{-}bonded}$ / kcal·mol^{-1}	E_{bonded} / kcal·mol^{-1}
bridging	1st DPPA	124	-112	235
	2nd DPPA	92	-147	239
	3rd DPPA	356	-88	444
chelating	1st DPPA	214	-87	301
	2nd DPPA	219	-82	301
	3rd DPPA	236	-74	310

Adsorption properties

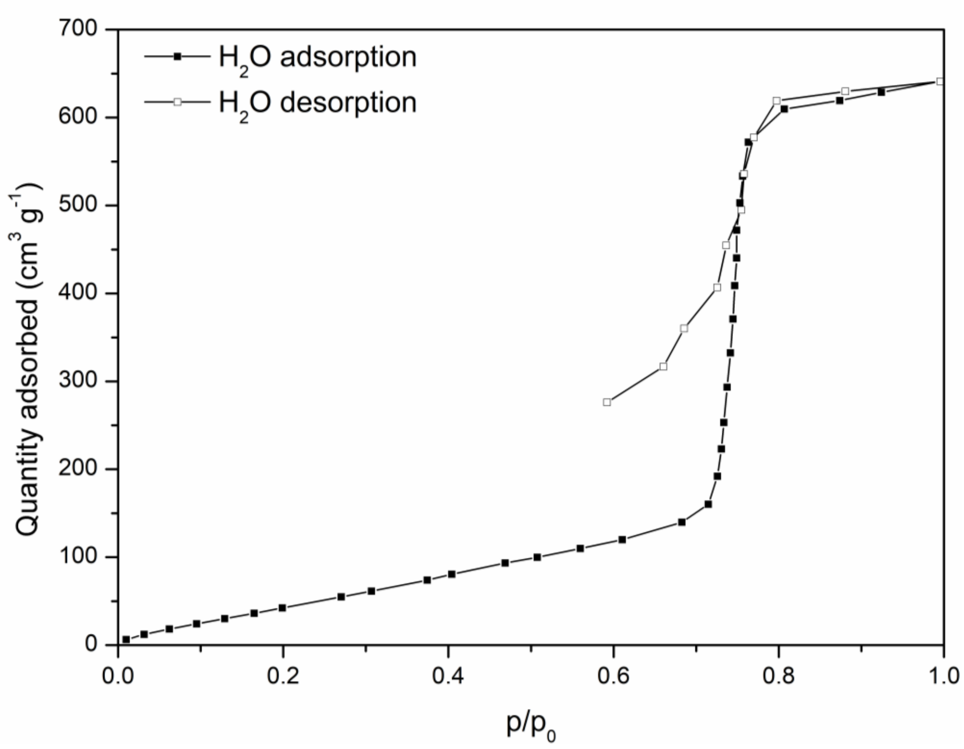

Figure S17: Adsorption isotherm of H_2O vapors for PCN-222 measured at 298 K.

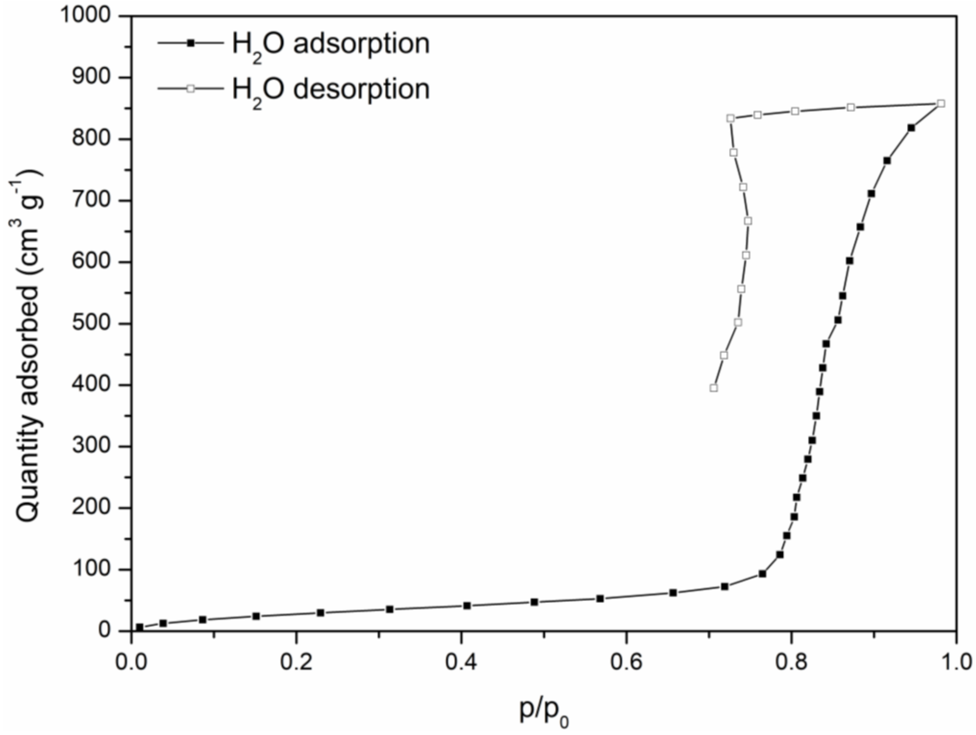

Figure S18: Adsorption isotherm of H_2O vapors for PCN-222+DPPA measured at 298 K.

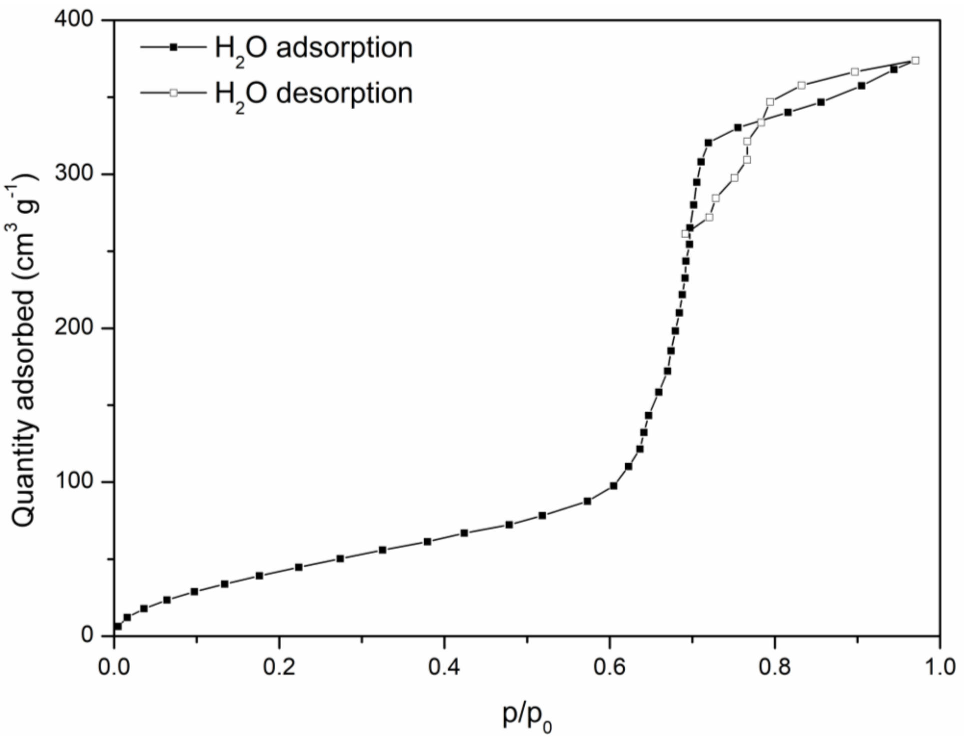

Figure S19: Adsorption isotherm of H_2O vapors for PCN-224 measured at 298 K.

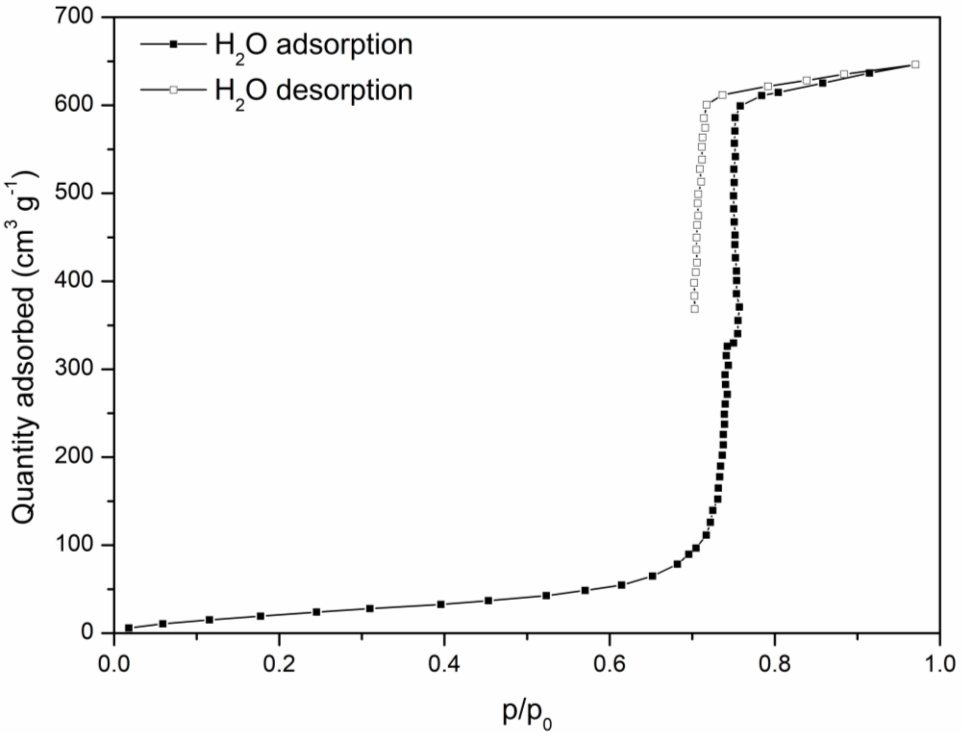

Figure S20: Adsorption isotherm of H_2O vapors for PCN-224+DPPA measured at 298 K.

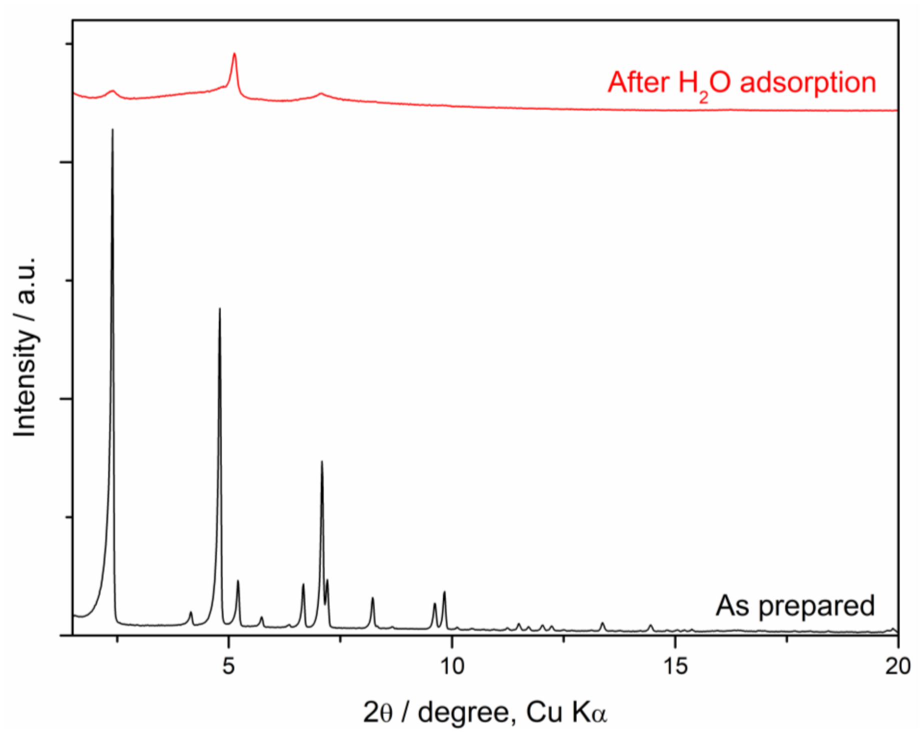

Figure S21: Powder XRD pattern of PCN-222 before (bottom) and after (top) the measurement of water uptake. Diffractograms are shifted vertically to avoid overlaps.

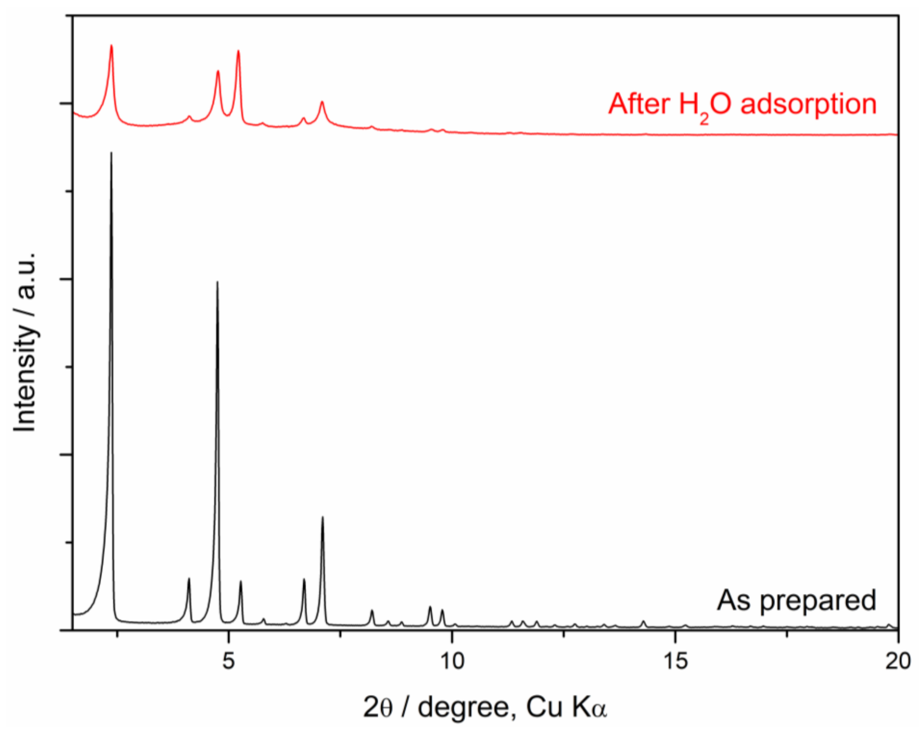

Figure S22: Powder XRD pattern of PCN-222+DPPA before (bottom) and after (top) the measurement of water uptake. Diffractograms are shifted vertically to avoid overlaps.

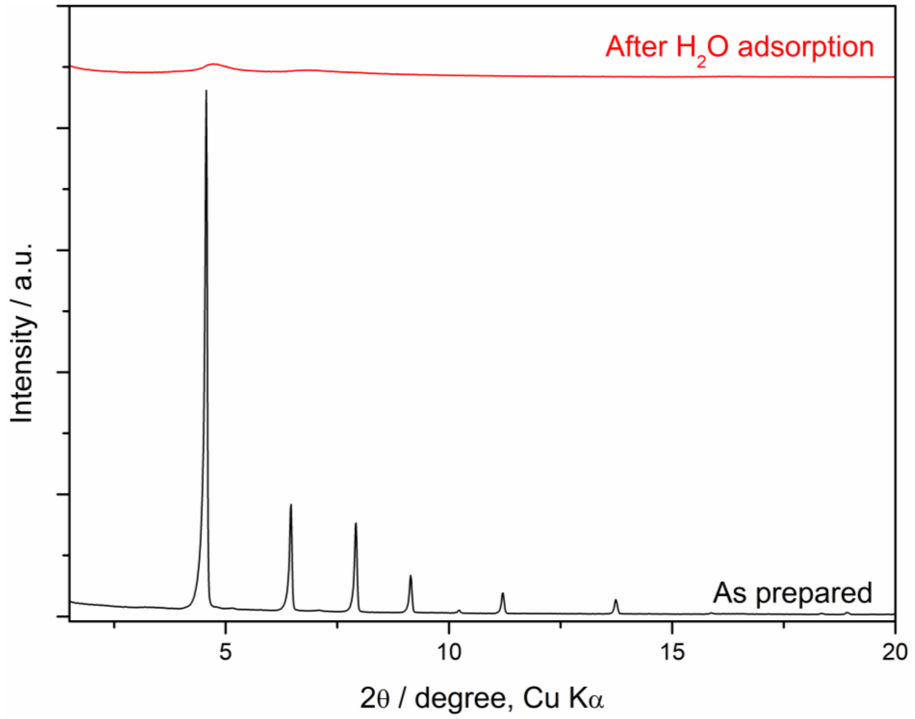

Figure S23: Powder XRD pattern of PCN-224 before (bottom) and after (top) the measurement of water uptake. Diffractograms are shifted vertically to avoid overlaps.

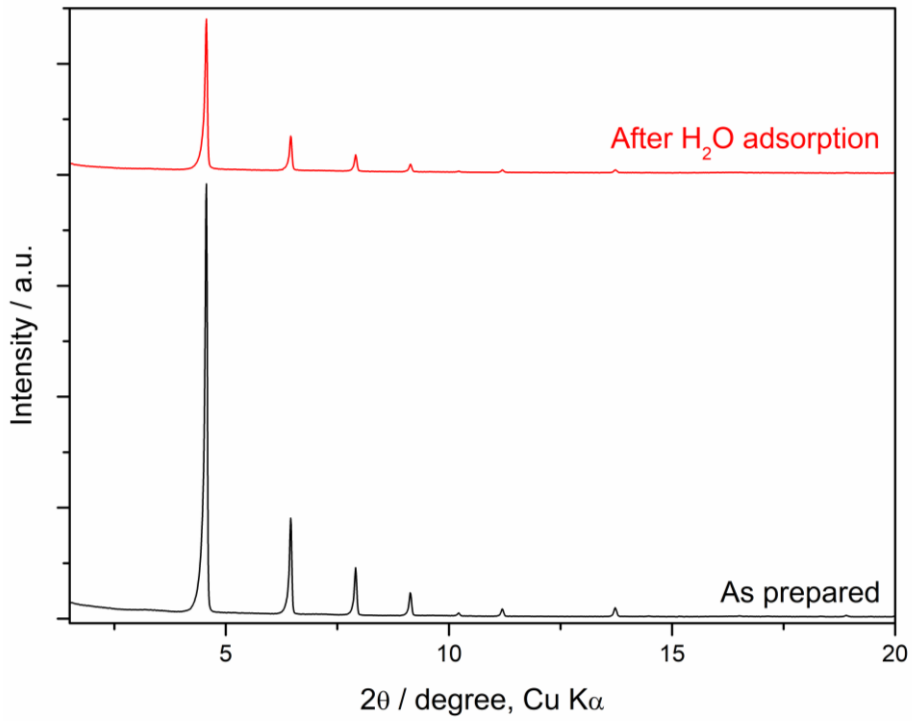

Figure S24: Powder XRD pattern of PCN-224+DPPA before (bottom) and after (top) the measurement of water uptake. Diffractograms are shifted vertically to avoid overlaps.

Stability at defined air humidity

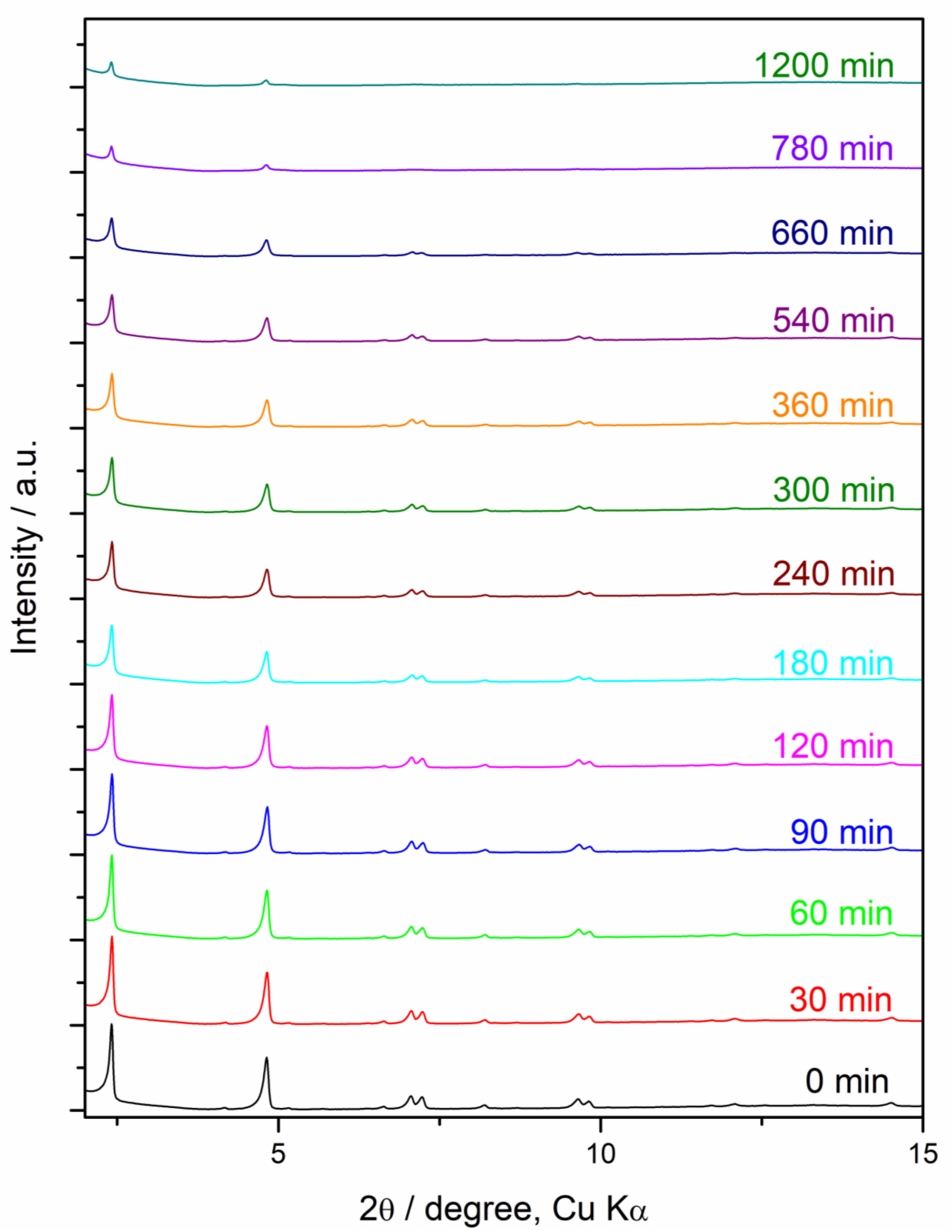

Figure S25: Time dependence of the powder diffraction patterns of PCN-222 exposed to 75% relative humidity. Diffractograms are shifted vertically to avoid overlaps.

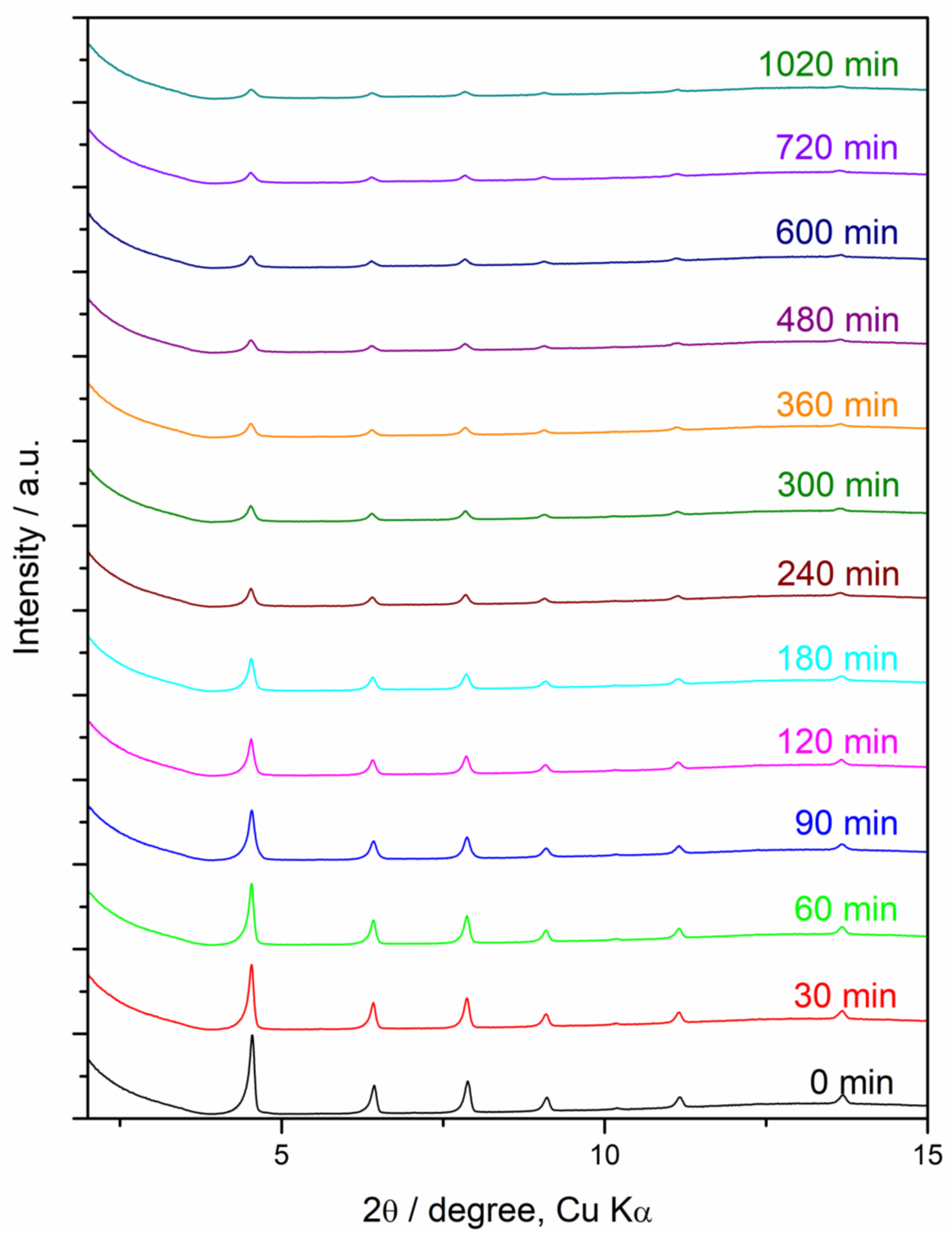

Figure S26: Time dependence of the powder diffraction patterns of PCN-224 exposed to 75% relative humidity. Diffractograms are shifted vertically to avoid overlaps.

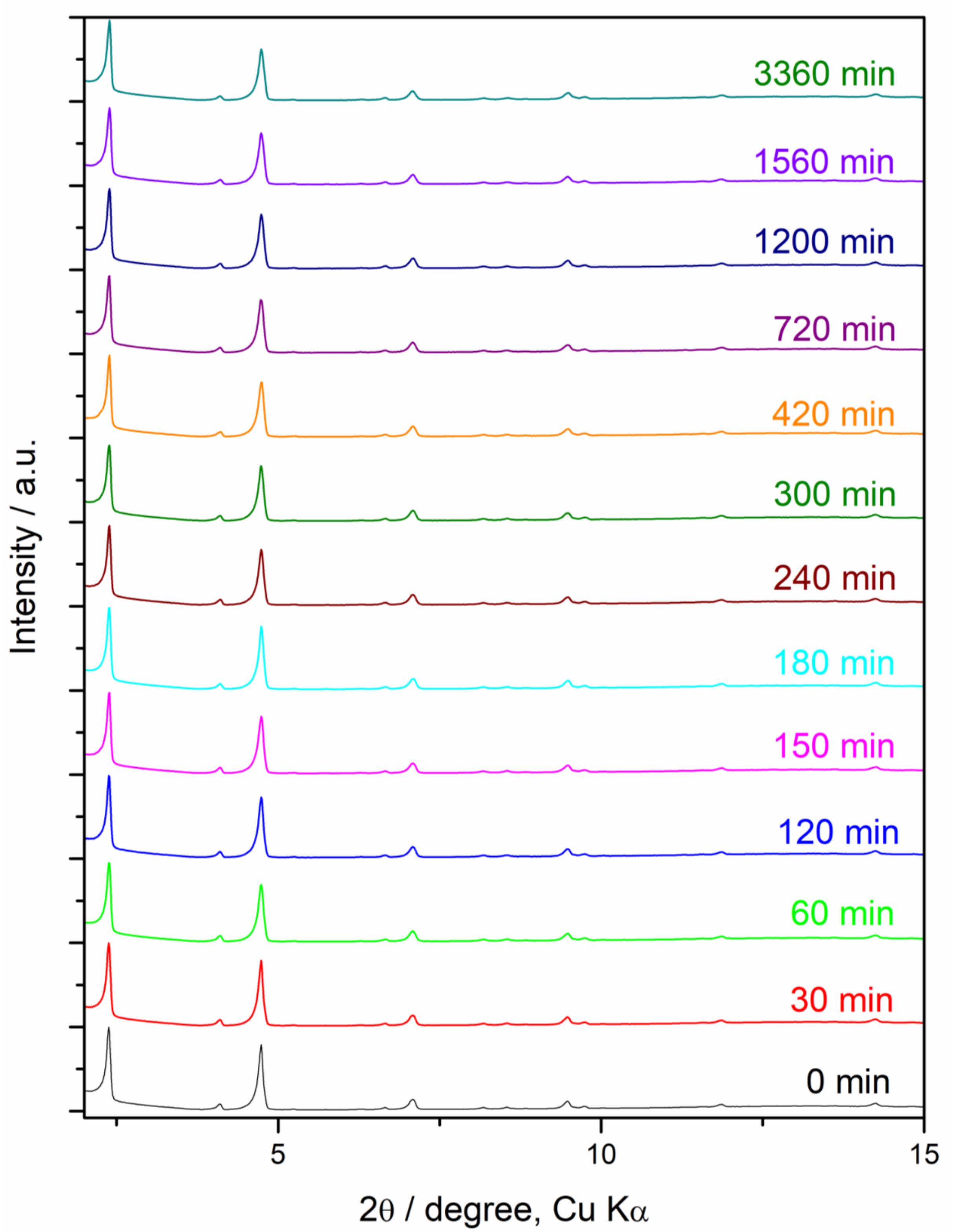

Figure S27: Time dependence of the powder diffraction patterns of PCN-222+DPPA exposed to 75% relative humidity. Diffractograms are shifted vertically to avoid overlaps.

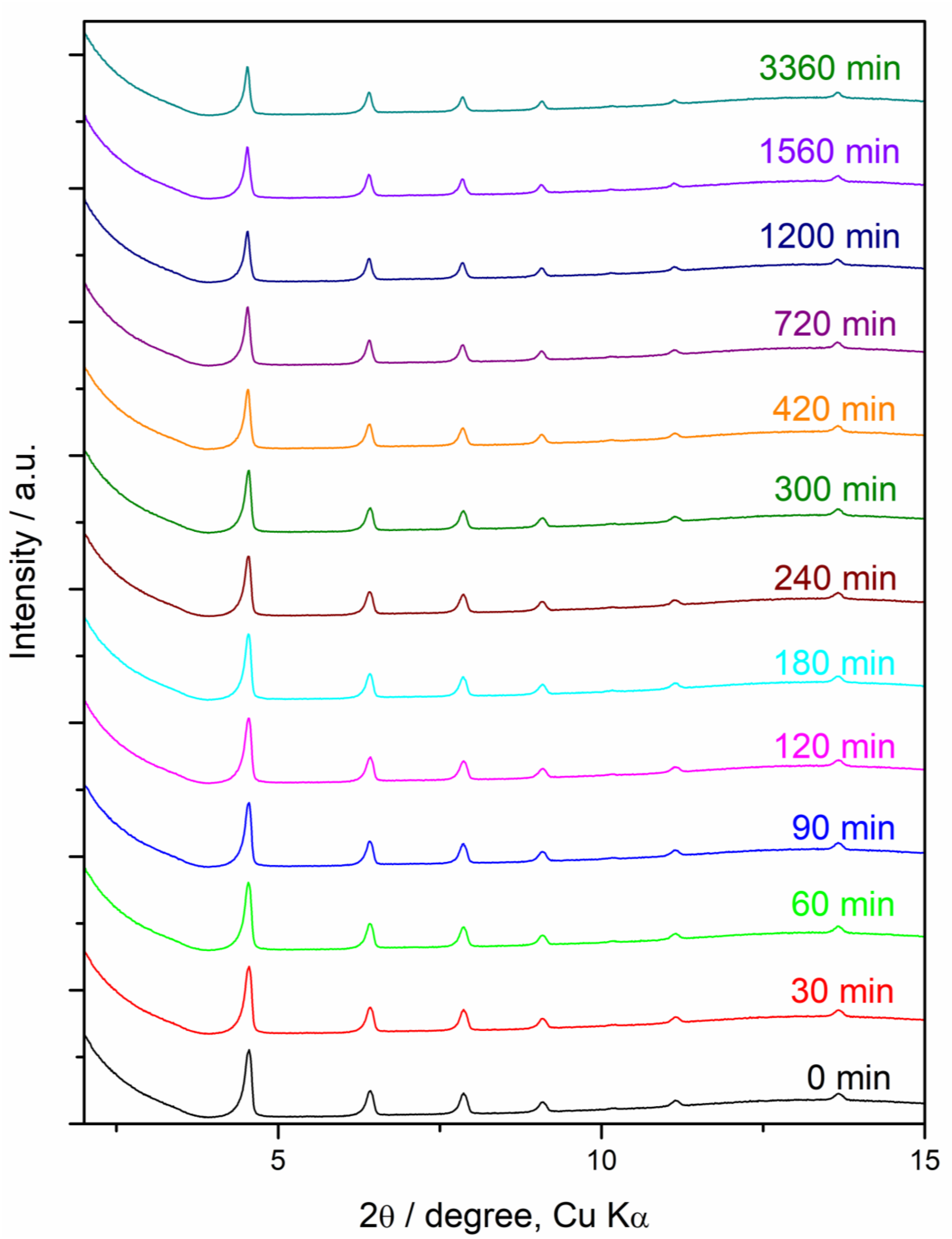

Figure S28: Time dependence of the powder diffraction patterns of PCN-224+DPPA exposed to 75% relative humidity. Diffractograms are shifted vertically to avoid overlaps.

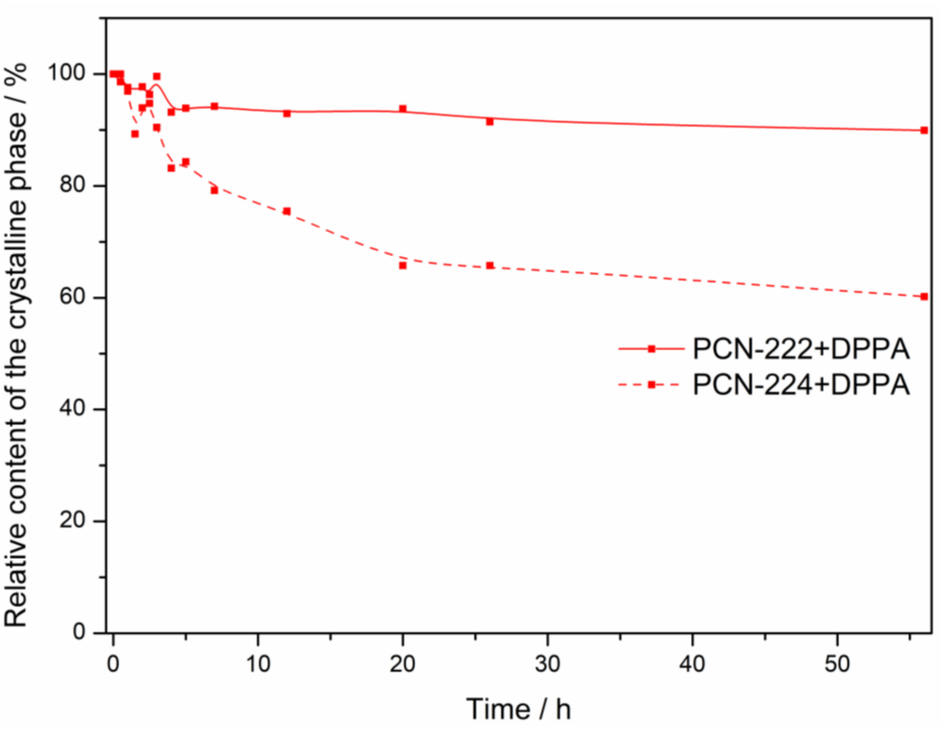

Figure S29: Long-term time dependence of the relative content of crystalline phase in PCN-222+DPPA (solid line) and PCN-224+DPPA (dashed line) upon exposure to 75% relative humidity.

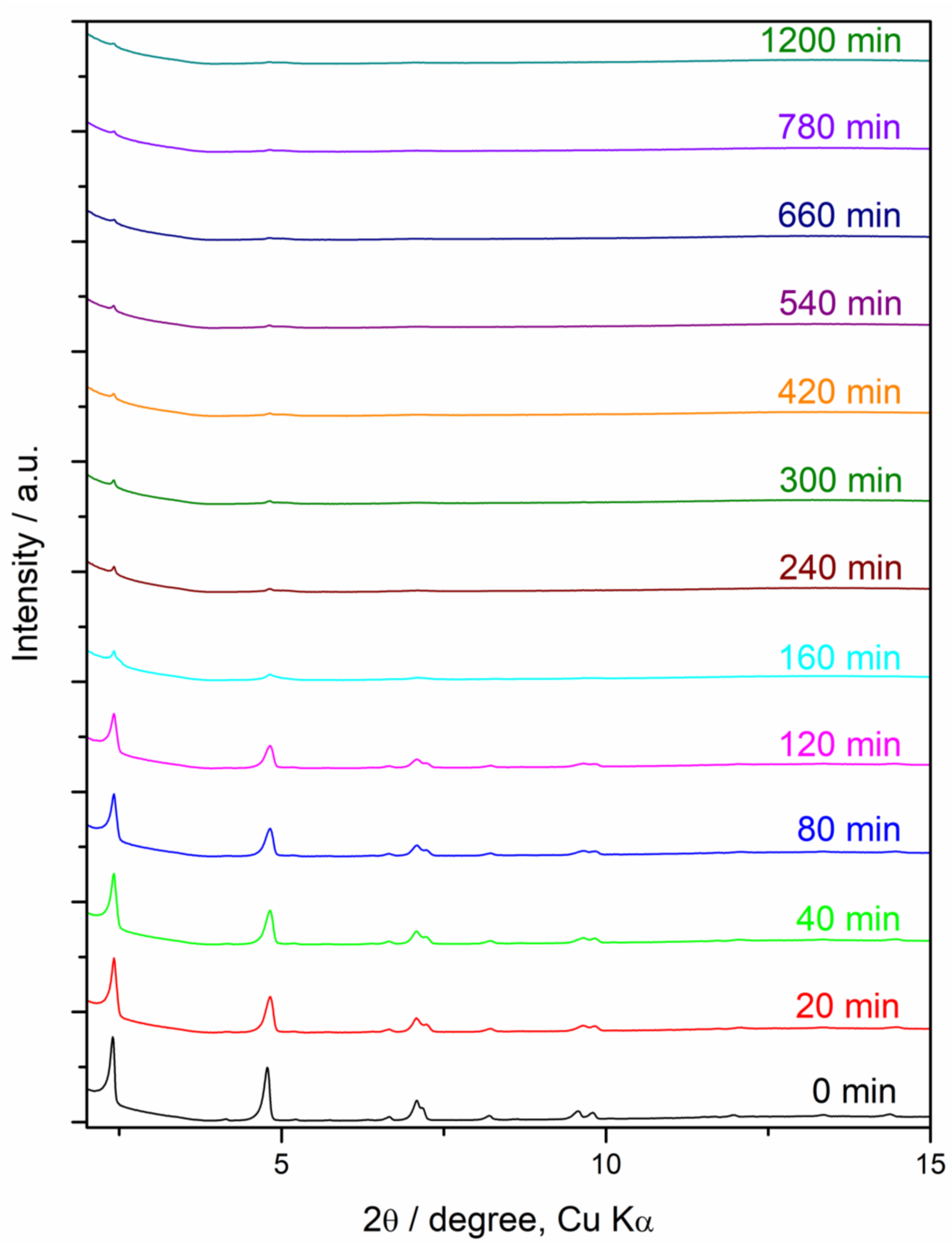

Figure S30: Time dependence of the powder diffraction patterns of PCN-222 exposed to 92% relative humidity. Diffractograms are shifted vertically to avoid overlaps.

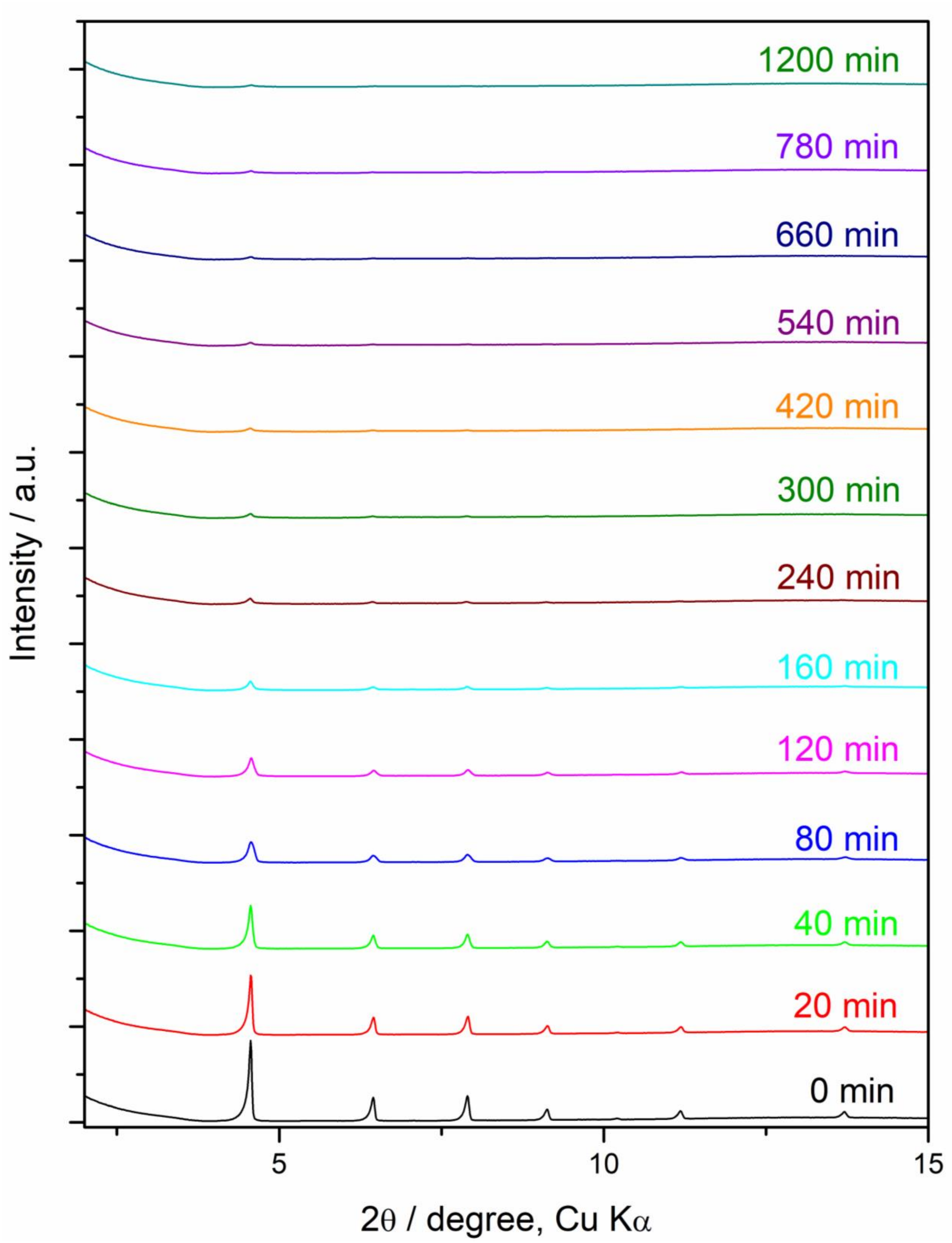

Figure S31: Time dependence of the powder diffraction patterns of PCN-224 exposed to 92% relative humidity. Diffractograms are shifted vertically to avoid overlaps.

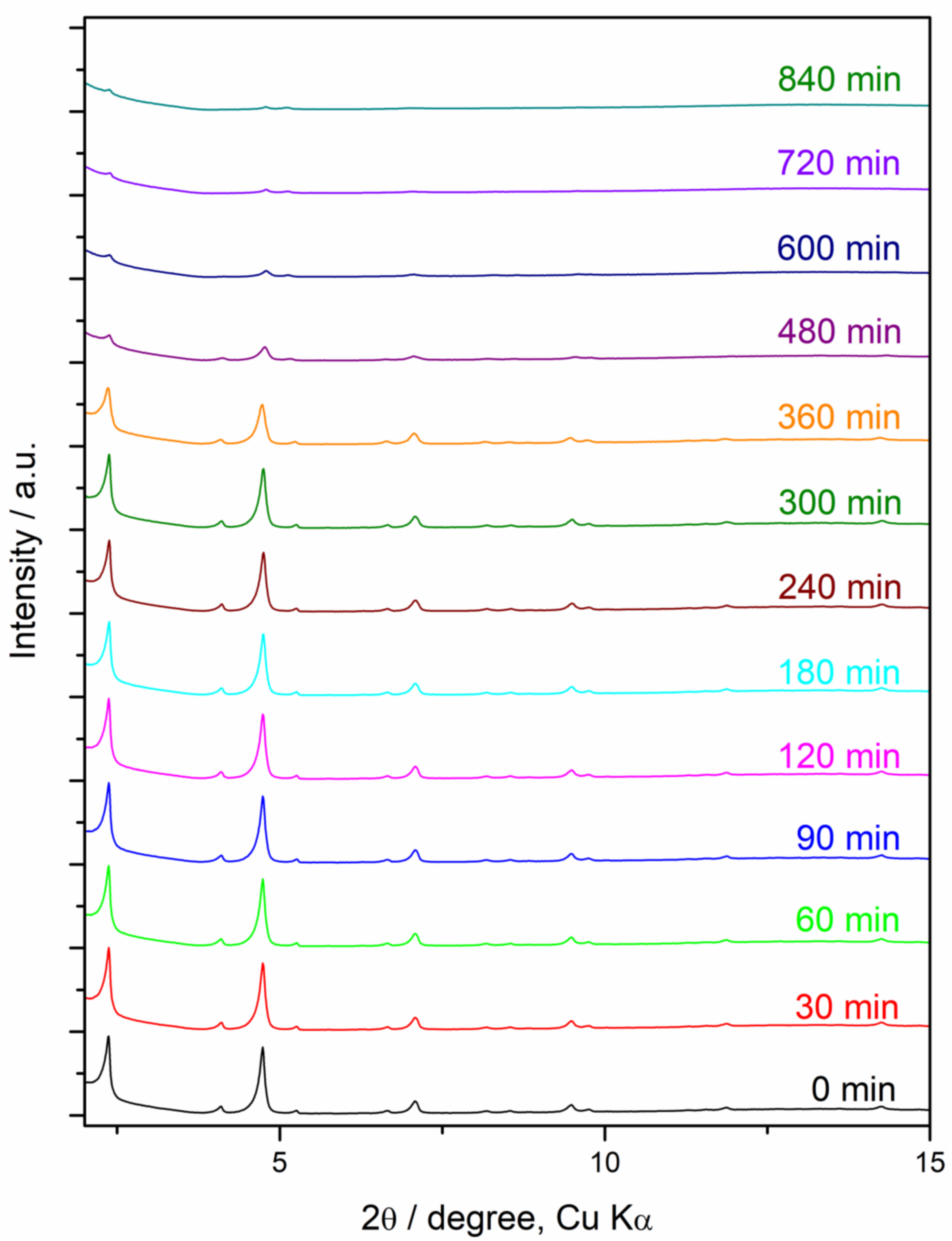

Figure S32: Time dependence of the powder diffraction patterns of PCN-222+DPPA exposed to 92% relative humidity. Diffractograms are shifted vertically to avoid overlaps.

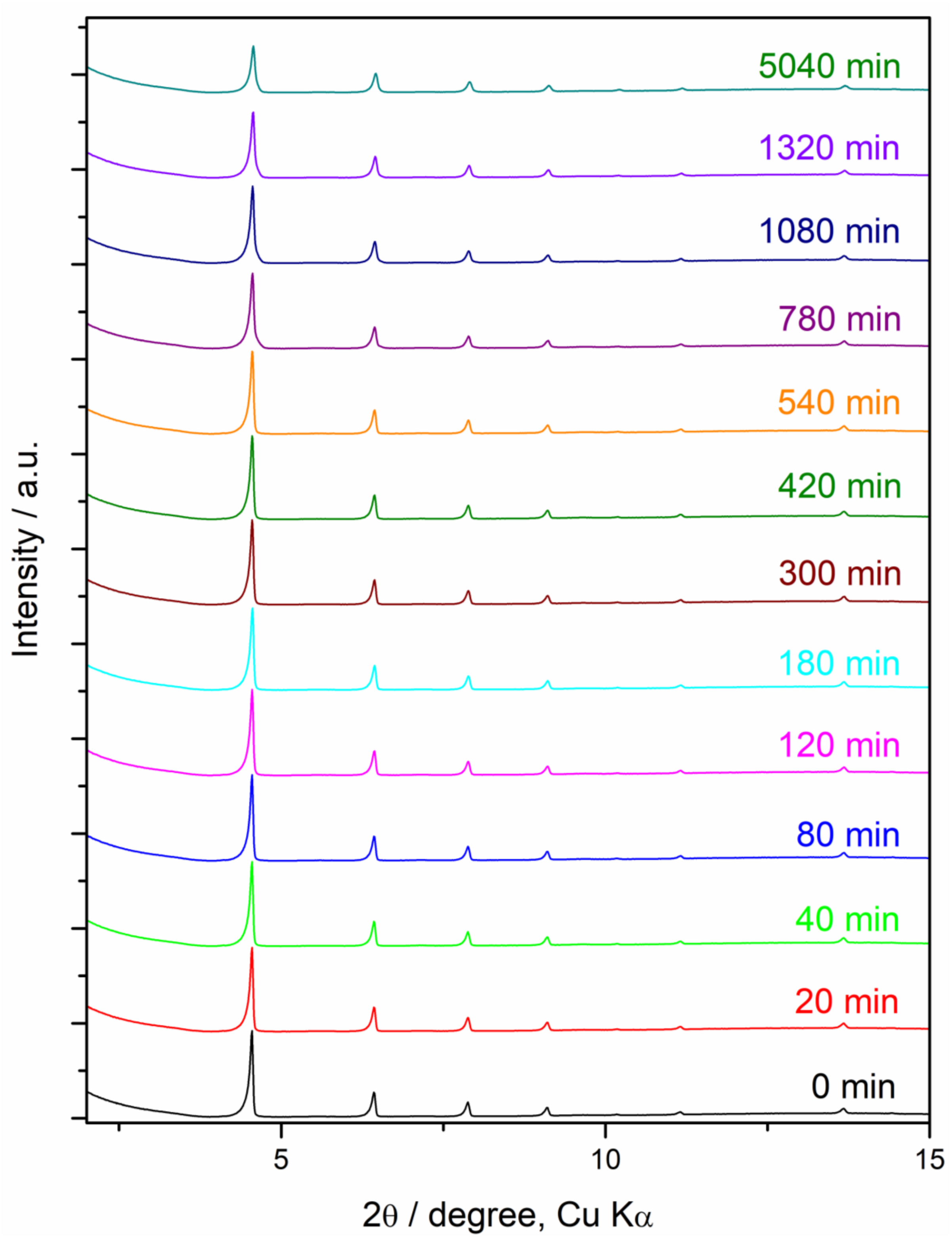

Figure S33: Time dependence of the powder diffraction patterns of PCN-224+DPPA exposed to 92% relative humidity. Diffractograms are shifted vertically to avoid overlaps.

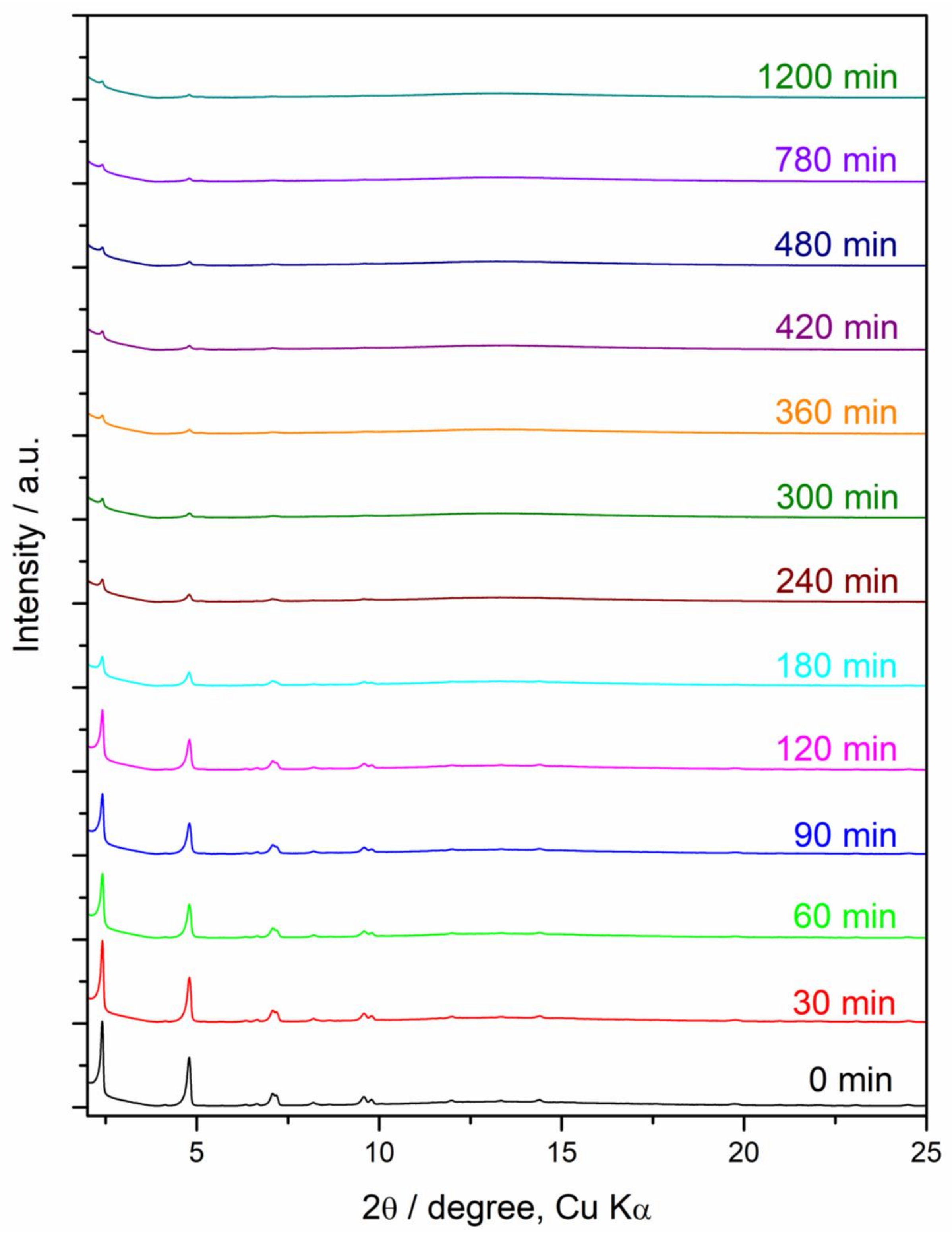

Figure S34: Time dependence of the powder diffraction patterns of Im@PCN-222 exposed to 75% relative humidity. Diffractograms are shifted vertically to avoid overlaps.

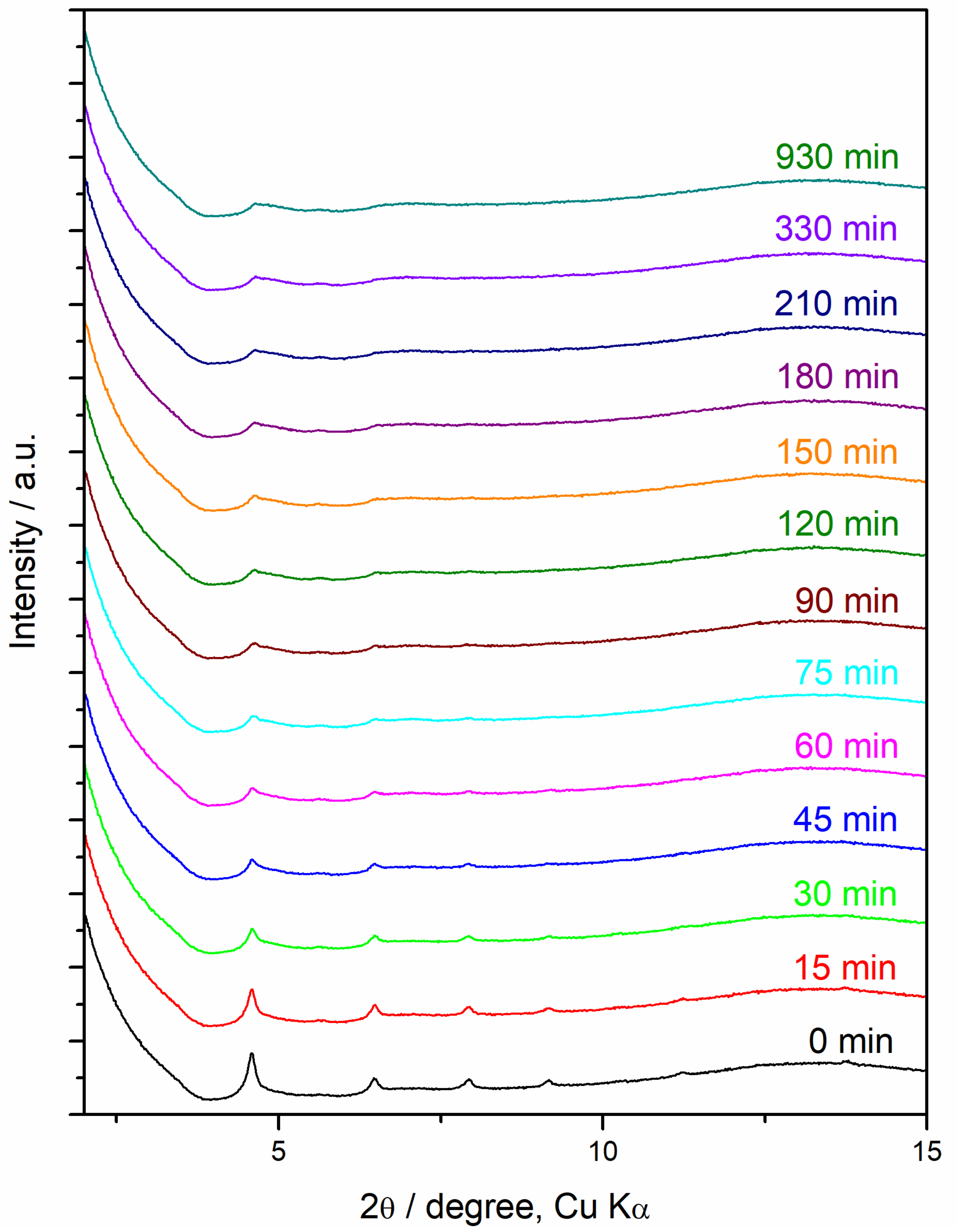

Figure S35: Time dependence of the powder diffraction patterns of Im@PCN-224 exposed to 75% relative humidity. Diffractograms are shifted vertically to avoid overlaps.

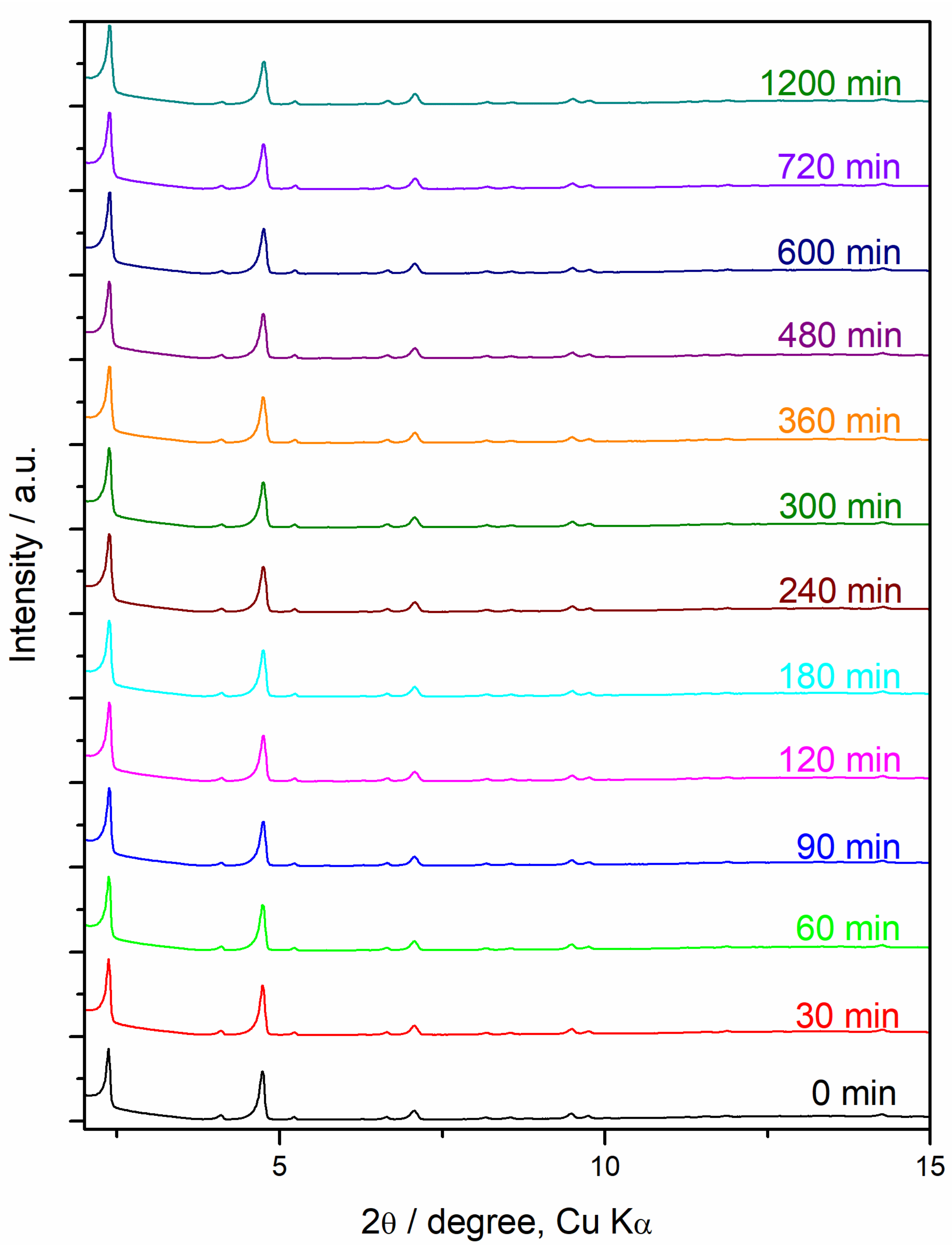

Figure S36: Time dependence of the powder diffraction patterns of Im@PCN-222+DPPA exposed to 75% relative humidity. Diffractograms are shifted vertically to avoid overlaps.

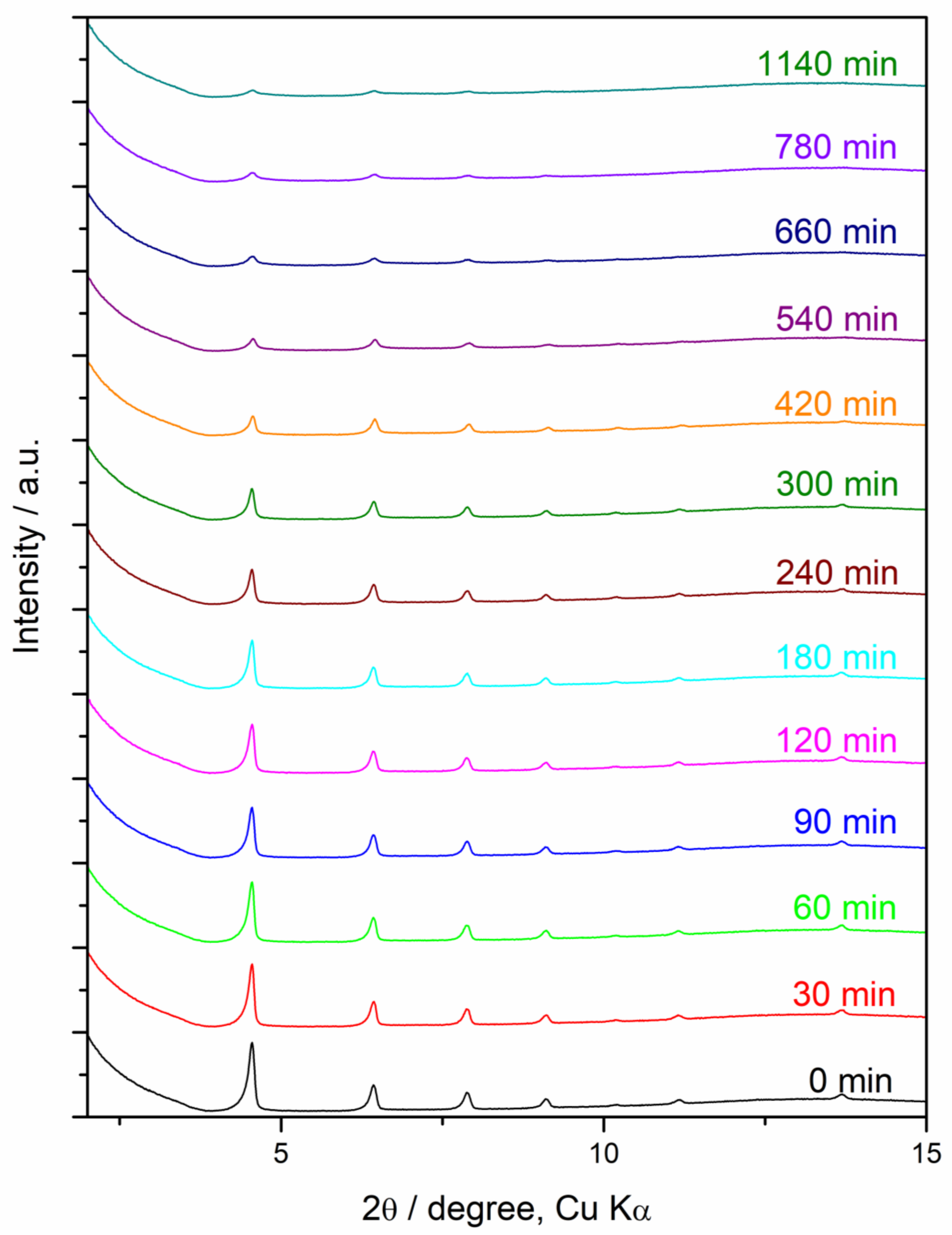

Figure S37: Time dependence of the powder diffraction patterns of Im@PCN-224+DPPA exposed to 75% relative humidity. Diffractograms are shifted vertically to avoid overlaps.

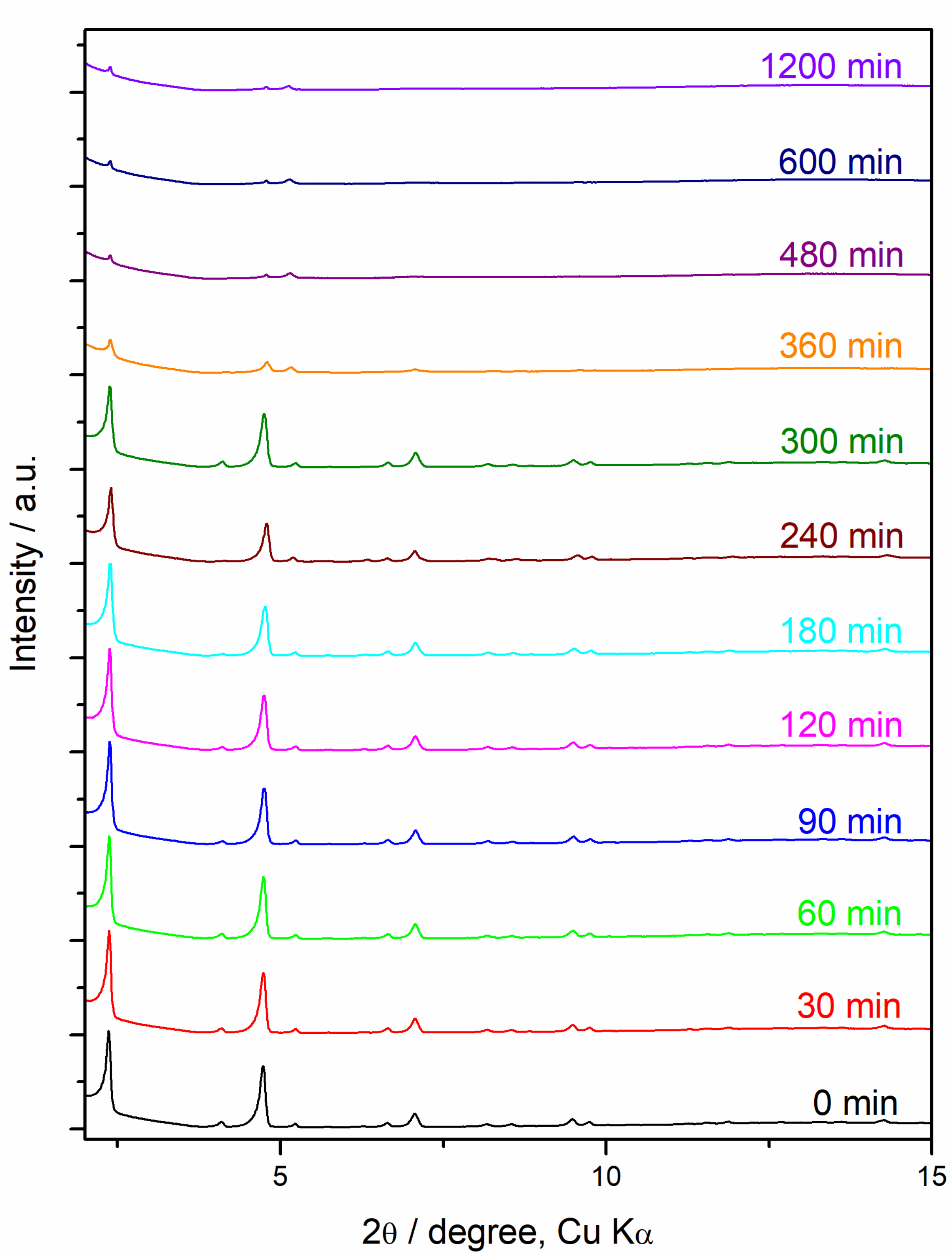

Figure S38: Time dependence of the powder diffraction patterns of Im@PCN-222+DPPA exposed to 92% relative humidity. Diffractograms are shifted vertically to avoid overlaps.

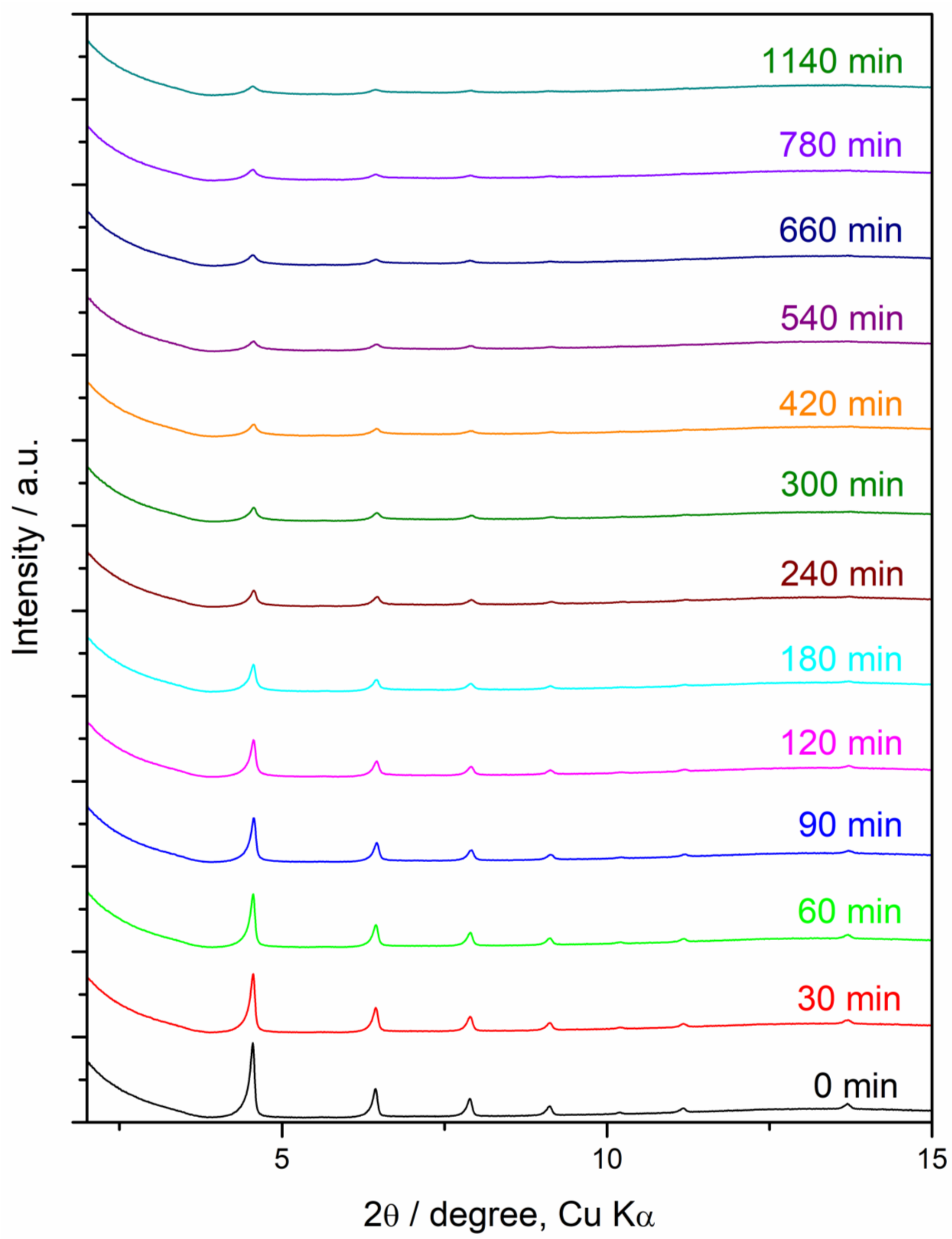

Figure S39: Time dependence of the powder diffraction patterns of Im@PCN-224+DPPA exposed to 92% relative humidity. Diffractograms are shifted vertically to avoid overlaps.

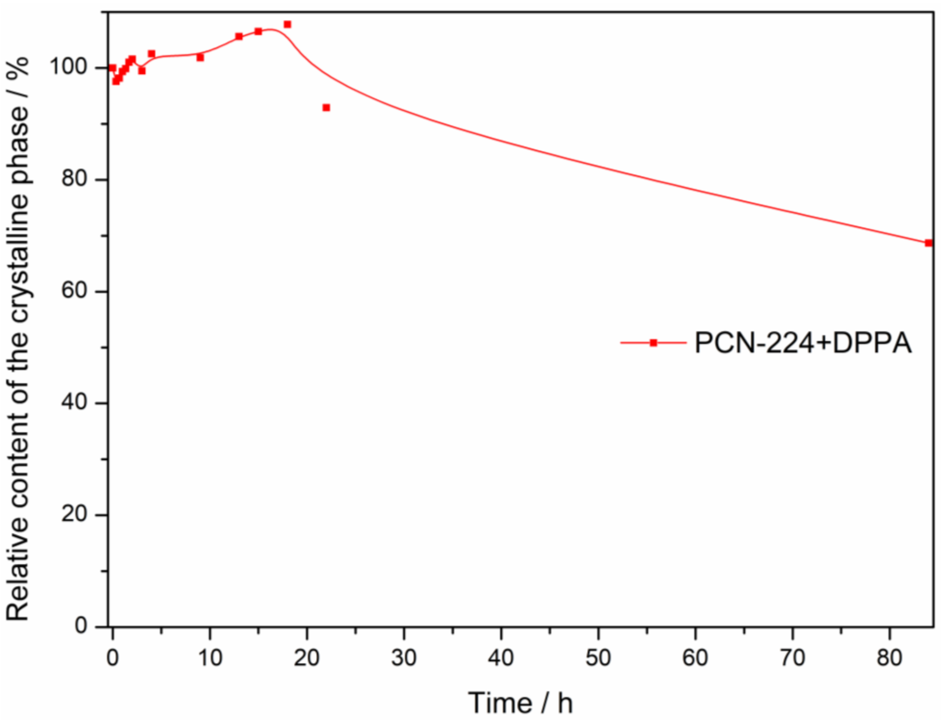

Figure S40: Long-term time dependence of the relative content of crystalline phase in PCN-224+DPPA upon exposure to 92% relative humidity.

Evaluation of the structural changes by infrared and Raman spectroscopy

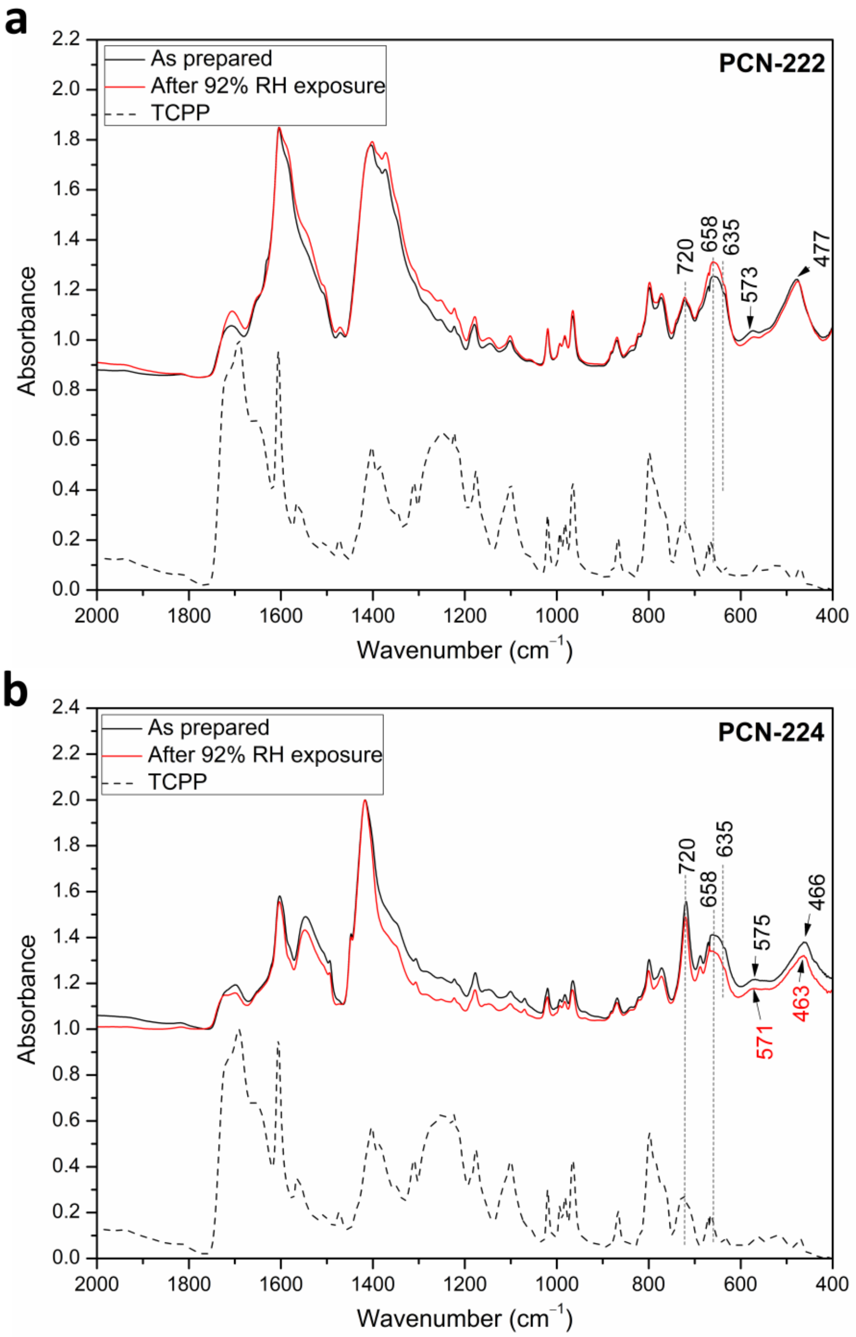

Figure S41: Infrared spectra of (a) PCN-222 and (b) PCN-224 measured in pressed KBr pellets before (black) and after (red) the exposure to 92% relative humidity for 24 h, comparison with the spectrum of TCPP ligand (dashed line). The spectra are vertically shifted to avoid overlaps.

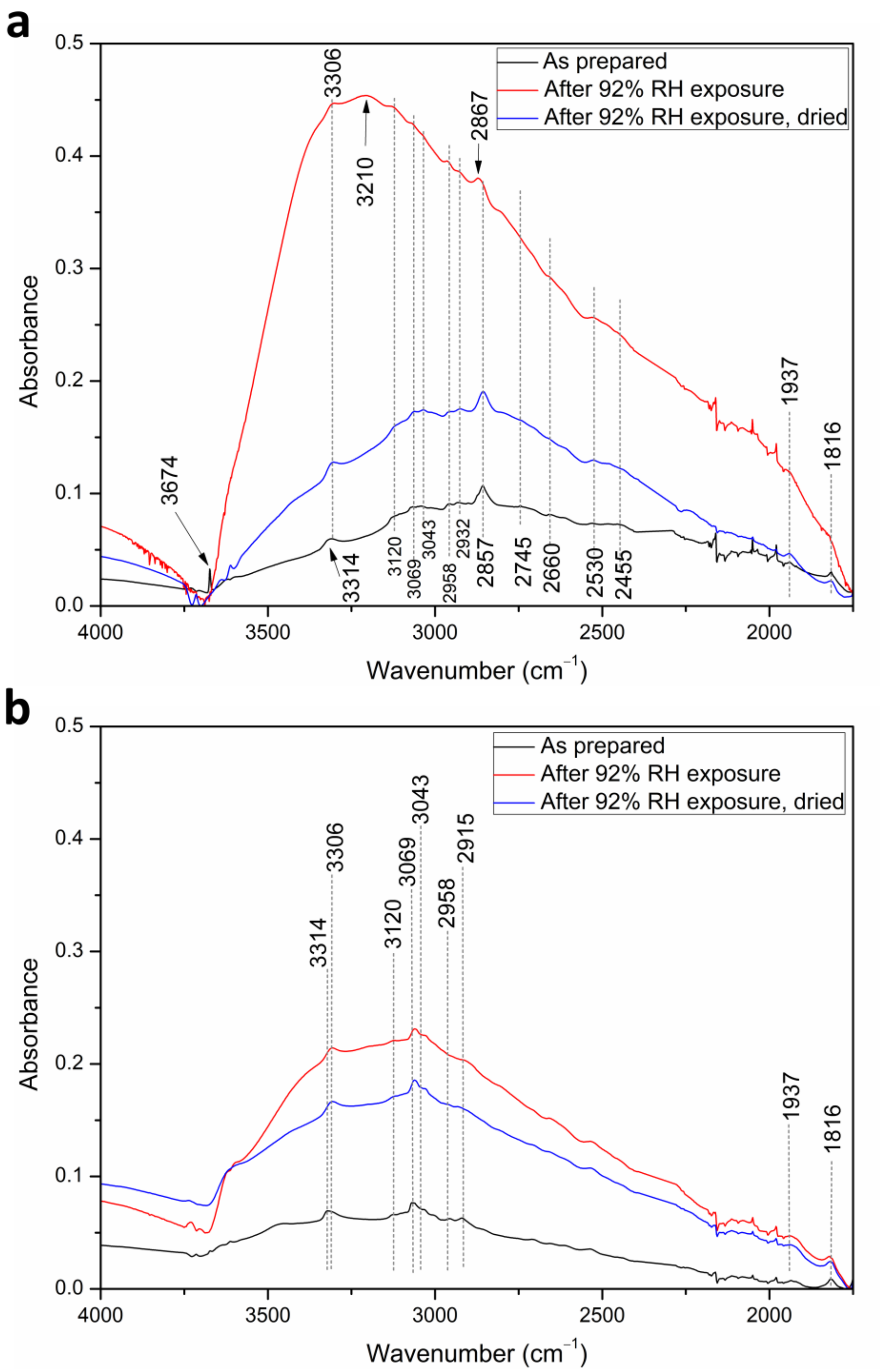

Figure S42: Infrared spectra of (a) PCN-222 and (b) PCN-224 measured on ATR before (black) and after (red) the exposure to 92% relative humidity for 24 h and after drying of the post-exposure sample (blue). For better visibility only 4000 – 1800 cm^{-1} region is presented in this figure. The spectra are vertically shifted to avoid overlaps.

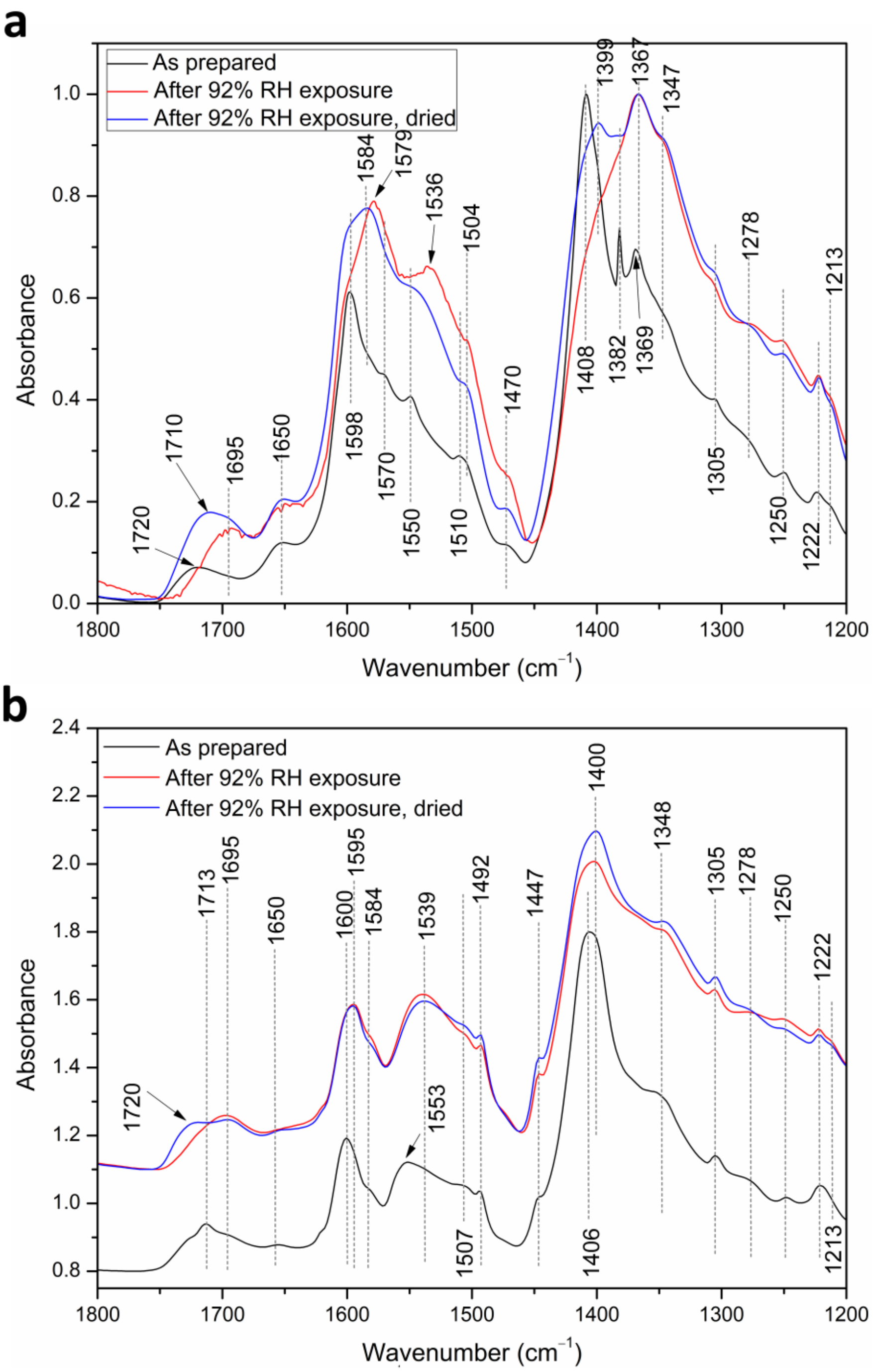

Figure S43: Infrared spectra of (a) PCN-222 and (b) PCN-224 measured on ATR before (black) and after (red) the exposure to 92% relative humidity for 24 h and after drying of the post-exposure sample (blue). For better visibility only 1800 – 1200 cm^{-1} region is presented in this figure. The spectra are vertically shifted to avoid overlaps.

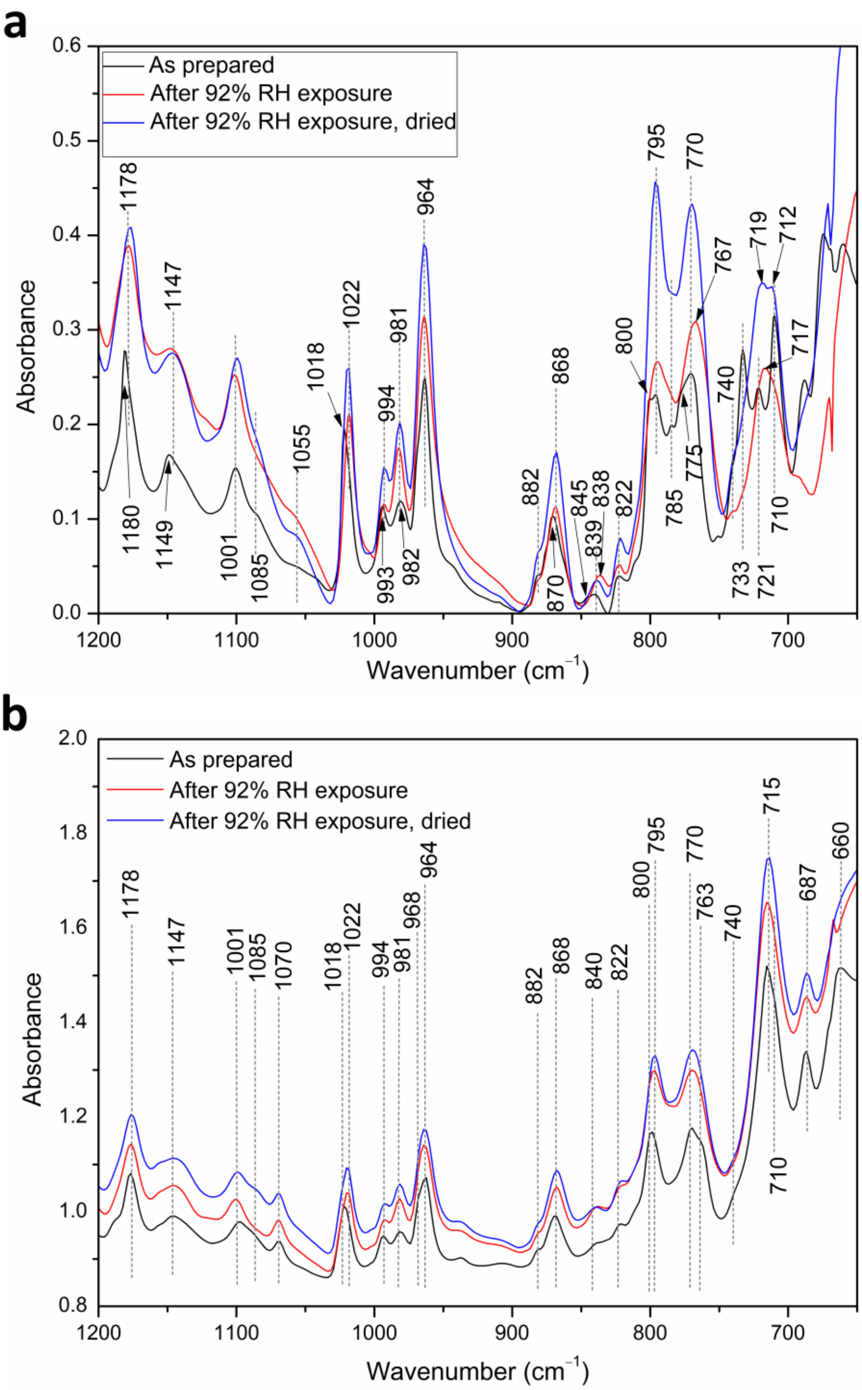

Figure S44: Infrared spectra of (a) PCN-222 and (b) PCN-224 measured on ATR before (black) and after (red) the exposure to 92% relative humidity for 24 h and after drying of the post-exposure sample (blue). For better visibility only 1200 – 600 cm^{-1} region is presented in this figure. The spectra are vertically shifted to avoid overlaps.

Table S3: Summary of the bands in the FTIR spectra of TCPP linker, PCN-222 and PCN-224 samples and their attribution based on the literature.[1–7] Positions of the bands attributed to acetone and *N*,*N*-dimethylformamide were taken from NIST database.

TCPP	PCN-222				PCN-224				Attribution
	As prepared	After 92% RH exposure	After 92% RH exposure, dried	KBr pelletts	As prepared	After 92% RH exposure	After 92% RH exposure, dried	KBr pelletts	
	3674 m sharp								OH stretching vibrations of the hydroxyl group at μ3 sites
3410 br w					3451 w	3451 w	3451 w		NH of pyrrole rings of the linker, OH of water H-bonded to Zr—O
3308 w	3314 w	3306 w	3014 w		3314 m	3314 m	3314 m		NH of pyrrole rings of the linker, OH of water H-bonded to Zr—O
2900 m br	3000 m br	3210 vs br	3000 m br		3000 m br	3000 m br	3000 m br		OH stretching of adsorbed water and free acid groups
	3120 w	3120 w	3120 w		3120w	3120w	3120w		Aromatic CH stretching
3070 w br	3069 w	3069 w	3069 w		3069 w	3069 w	3069 w		Aromatic CH stretching
	3043 w	3043 w	3043 w						Aromatic CH stretching, asymmetric CH_3 stretching of acetone
	2958 w	2958 w	2958 w						Asymmetric CH_3 stretching of acetone
	2932 w	2932 w	2932 w						CH_3 stretching of acetone and DMF
2915 w	2900 w				2915 w	2915 w	2915 w		CH stretching of free formic acid
	2857 m	2867 w	2857 m						Symmetric CH_3 stretching of DMF, CH stretching of formic

									acid coordinated to the Zr cluster
	2745 w	2745 sh	2745 sh						OH stretching of water H-bonded to hydroxyl ligand
2605 w									Overtone
2510 w									Overtone
1937 vw	1937 w	1937 w	1937 w	1937 w	1937 w	1937 w	1937 w	1937 vw	Overtone
1816 vw	1816 w	1816 w	1816 w	1813 w	1816 m	1816 w	1816 w	1813 vw	Overtone
1713 s sh, 1680 s	1720 m	1693 m	1710 m, 1695 sh	1706 m	1713 w		1720 m	1706 m	C=O stretching of COOH groups and acetone
1640 s	1650 m	1650 m	1650 m	1650 sh	1650 m	1650 w	1650 w	1650 m	DMF, water
1602 s	1598 vs	1598 sh	1598 sh	1604 vs	1602 s	1593 s	1593 s	1604 s	Phenyl ring stretching, asymmetric stretching of COO^- groups of bridging formate and the linker
	1584 sh, 1570 sh	1579 vs	1584 vs	1590 sh	1580 sh	1580 sh	1580 sh	1590 sh	Asymmetric stretching of COO^- groups of free carboxylates
1560 m	1550 m							1560 s	CH rocking on the macrocycle
		1550 s br	1536 s	1544 sh	1553 s	1540 s	1540 s	1544 sh	Asymmetric stretching of COO^- groups in bidentate coordination
1505 m	1510 w	1504 sh	1504 w	1507 w	1507 m	1507 m	1507 m	1507 w	NH deformation
					1492 s	1492 s	1492 s		Benzoic acid

1470 m	1470 w	1470 w	1470 w	1472 w		1470 w sh	1470 w sh	1472 w	Macrocycle stretching
Sh		Sh	sh	Sh	1447 w	1447 w	1447 w	Sh	Symmetric stretching of COO^{-} of the linker in bidentate coordination to a Zr atom, CH rocking on phenyl rings
1400 s	1408 vs	sh	1399 vs	1404 vs	1405 vs	1405 sh, 1400 vs	1405 sh, 1400 vs	1404 vs	Symmetric stretching of COO^{-} groups of the linker bridging two Zr atoms
1382 s	1382 s sharp		sh	1388 m					Pyrrole ring stretching
	1369 s	1367 vs	1367 vs	1373 vs				1373 sh	Symmetric stretching of free COO^{-} groups, pyrrole ring stretching, acetone, DMF
1347 m	1347 sh	1347 sh	1347 sh	1350 sh	1345 s	1345 s	1345 s	1350 sh	Symmetric stretching of COO^{-} groups in monodentate coordination to a Zr atom
1309 m	1305 w	Sh	sh	1305 sh	1305 w	1305 w	1305 w	1305 sh	pyrrole ring stretching, COOH C—O stretching
Sh	1278 w	1278 w	1278 w	1278 w	1278 w	1278 w	1278 w	1278 w	COOH C—O stretching
Sh	1250 w	1250 w	1250 w	1251 w	1250 w	1250 w	1250 w	1251 w	Pyrrole ring stretching
1233, 1220	1222 w	1222 w	1222 w	1223 w	1222 w	1222 w	1222 w	1223 w	Pyrrole ring stretching, acetone
1212 m	1213 sh	1213 sh	1213 sh	1212 sh		1213 w	1213 w	1212 sh	Pyrrole ring stretching
1173 s	1180 m sharp	1178 m	1178 m	1179 m	1178 m	1178 m	1178 m	1179 m	CH rocking on phenyl rings
,	1149 w	1147 w	1147 w	1145 w	1147 w	1147 w	1147 w	1145 w	Pyrrole ring deformation
1097 s	1101 w	1101 w	1001 w	1102 w	1101 w	1101 w	1101 w	1102 w	CH rocking on phenyl rings

	1085 w	1085 sh	1085 sh		1070 w	1070 w	1070 w	1070 w	DMF, acetone
1058 sh	Br sh	1055 sh	1055 w						DMF
1018 m	1018 m	1022 m	1022 m	1020 m	1018 m	1022 m	1022 m	1020 m	Phenyl ring deformation
991 m	993 w	994 w	994 w	993 w	994 w	994 w	994 w	993 w	Deformation of the area around macrocycle—phenyl links
981 m	982 w	981 w	981 w	983 w	981 w	981 w	981 w	983 w	Macrocycle deformation
962 s	964 m	964 m	964 m	965 m	968 sh, 964 m	968 sh, 964 m	968 sh, 964 m	965 m	Phenyl CH wagging
880 vw	882 sh	882 sh	882 sh		882 sh	882 sh	882 sh		NH and $C_{\beta}H$ wagging, macrocycle deformation
865 m	870 w	868 w	868 w	870 w	870 w	868 w	868 w	870 w	NH and $C_{\beta}H$ wagging, macrocycle deformation
840 sh	839 w	838 w	838 w	838 w	839 w	839 w	839 w	838 w	NH and $C_{\beta}H$ wagging, macrocycle deformation
821 w	822 w	822 w	822 w	822 w	822 w	822 w	822 w	822 w	NH and $C_{\beta}H$ wagging, macrocycle deformation
791 s	795, 800 m	795 m	795 m	799 m	800 m	795 m	795 m	799 m	COO^- and COOH bending
781 s	805 w sharp								COO^- and COOH bending
	775 sh								COO^- and COOH bending
763 s	770 m struct.	767 m	770 m	773 m	770 m, 763 sh	770 m	770 m	773 m	COO^- and COOH bending
	733 m sharp		740 sh		740 sh	740 sh	740 sh		$Zr(\mu_3)$—O stretching
721 s	721 w	717 m	719 m	720 m	715 s	715 s	715 s	720 m	CH wagging on phenyl rings
709 s	710 m sharp		712 m						Zr—OC asymmetric stretching, CH wagging on phenyl rings

				658, 635 s				658, 635 s	Zr(μ_3)—O stretching
				573/ 571 w				575/ 571 w	Zr—OC symmetric stretching
				477/ 476 s				466/ 463 s	Zr(μ_3)—OH stretching

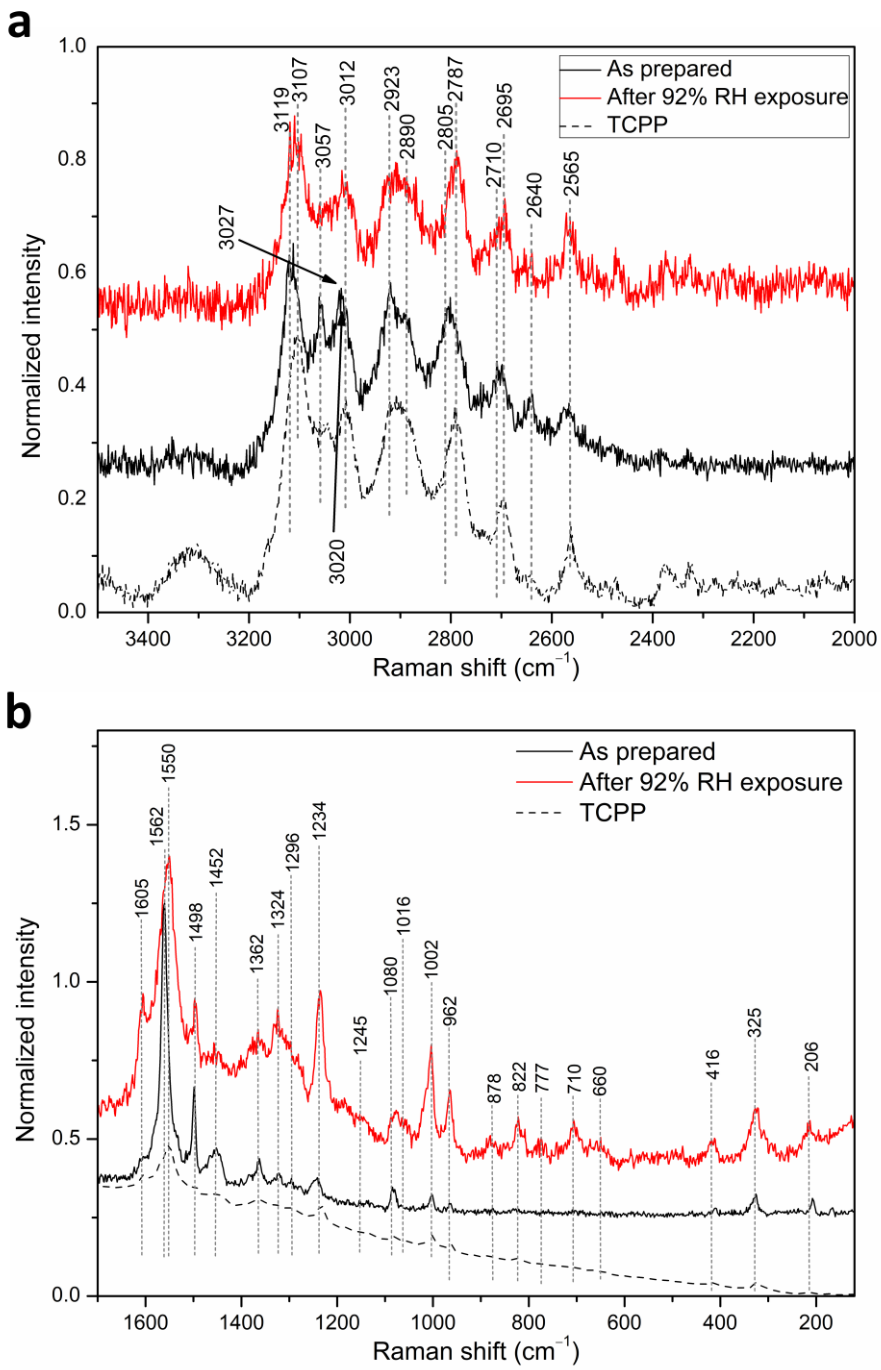

Figure S45: Average Raman spectra of PCN-222 before (black line) and after the exposure to 92% relative humidity for 24 h (red line) measured with 488 nm laser excitation in comparison with the spectrum of neat TCPP ligand (dashed line). The spectra are baseline corrected (subtraction of fluorescence) and normalized. For better visibility of individual bands, the spectra are separated into two regions presented in individual figures; (a) 3500 – 2000 cm^{-1} and (b) 1700 – 150 cm^{-1}. The spectra are vertically shifted to avoid overlaps.

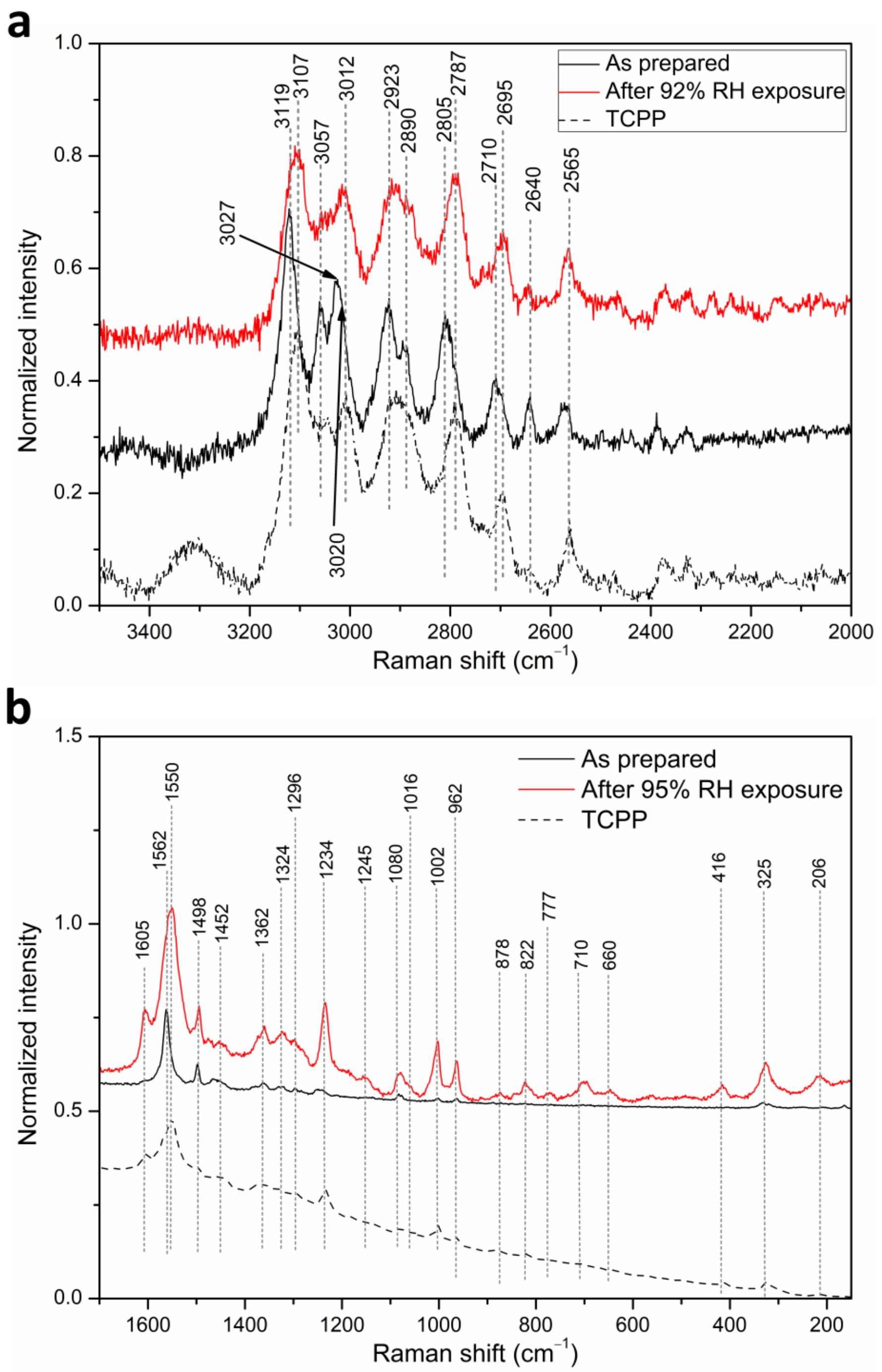

Figure S46: Average Raman spectra of PCN-224 before (black line) and after the exposure to 92% relative humidity for 24 h (red line) measured with 488 nm laser excitation in comparison with the spectrum of neat TCPP ligand (dashed line). The spectra are baseline corrected (subtraction of fluorescence) and normalized. For better visibility of individual bands, the spectra are separated into two regions presented in individual figures; (a) 3500 – 2000 cm^{-1} and (b) 1700 – 150 cm^{-1}. The spectra are vertically shifted to avoid overlaps.

Table S4: Summary of the bands in the Raman spectra of TCPP linker, PCN-222 and PCN-224 samples and their attribution based on the literature.[2,8–10]

TCPP	PCN-222		PCN-224		Attribution	Change upon degradation
	As prepared	After 92% RH exposure	As prepared	After 92% RH exposure		
1605 w	1605 w	1605 m		1605 m	phenyl ring stretching	
1550 s	1562 vs	1540 vs	1562 s	1562 vs	macrocycle C—C stretching	redshift
1498 w	1498 m	1498 m	1498 m	1498 m	macrocycle C—C stretching and **N—H rocking**	
1453 w	1453 m	1453 m	1453 w	1453 w	macrocycle C—C stretching and C_β—H rocking, COO^- symmetrical stretching	
1362 w	1362 m	1362 m, br	1362 w	1362 m	macrocycle C—C stretching and C—N bending	
1324 w	1324 w	1324 m, br	1324 w	1324 w	C—C stretching, C_β—H and **N—H rocking**	increase and redshift
1296 w	1296 w	1296 sh	1296 w	1296 w	phenyl ring C—C stretching	
	1234 m	1234 s	1245 m	1234 s	inter-ring C—C stretching between phenyl rings and the macrocycle	
		1138 w, br		1134 w	C_β—H and **N—H rocking**	
1080 w	1080 m	1080 w	1080 w	1080 m	C_β—H rocking	
		1016 sh		1016 sh	pyrrole ring stretching	redshift
1002 m	1002 m	1002 s	1002 w	1002 m	pyrrole ring stretching	
962 m	962 w	962 m	962 w	962 w	macrocycle breathing, **aromatic carboxylic acid skeletal vibration**	
		880 w		880 w	entire molecule bending, OH bending	
		822 w		822 w	**carboxylic acid group deformation**	
		815 sh		815 sh	**carboxylic acid group deformation**	
		777 w		777 w	**carboxylic OH bending**	

		710 w		710 w	out of plane twisting of the whole molecule, **COOH deformation**	
		660 w		660 w	out of plane twisting of the whole molecule, Zr-O cluster	
410 w	410 w	416 w		416 w	macrocycle breathing with phenyl rings moving in the opposite direction	
325 m	325 m	325 m	325 w	325 m	breathing of the whole molecule	decrease
206 w	206 w	206 w		206 w	out of plane deformation of the macrocycle, Zr—O(μ3) stretching	

Scanning electron microscopy images

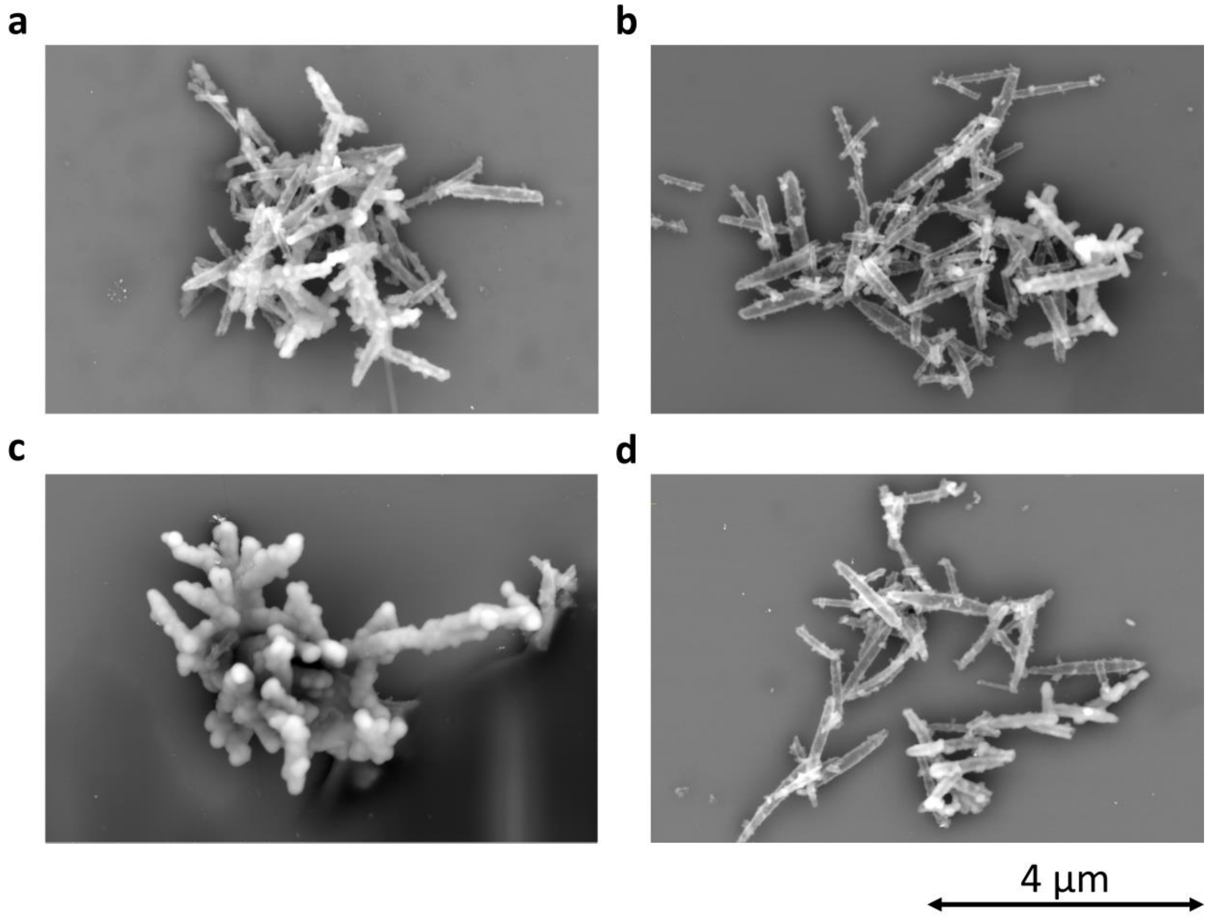

Figure S47: Scanning electron microscopy image of (a) PCN-222, (b) PCN-222+DPPA before and (c) PCN-222 and (d) PCN-222+DPPA after 24 h exposure to 92% relative humidity.

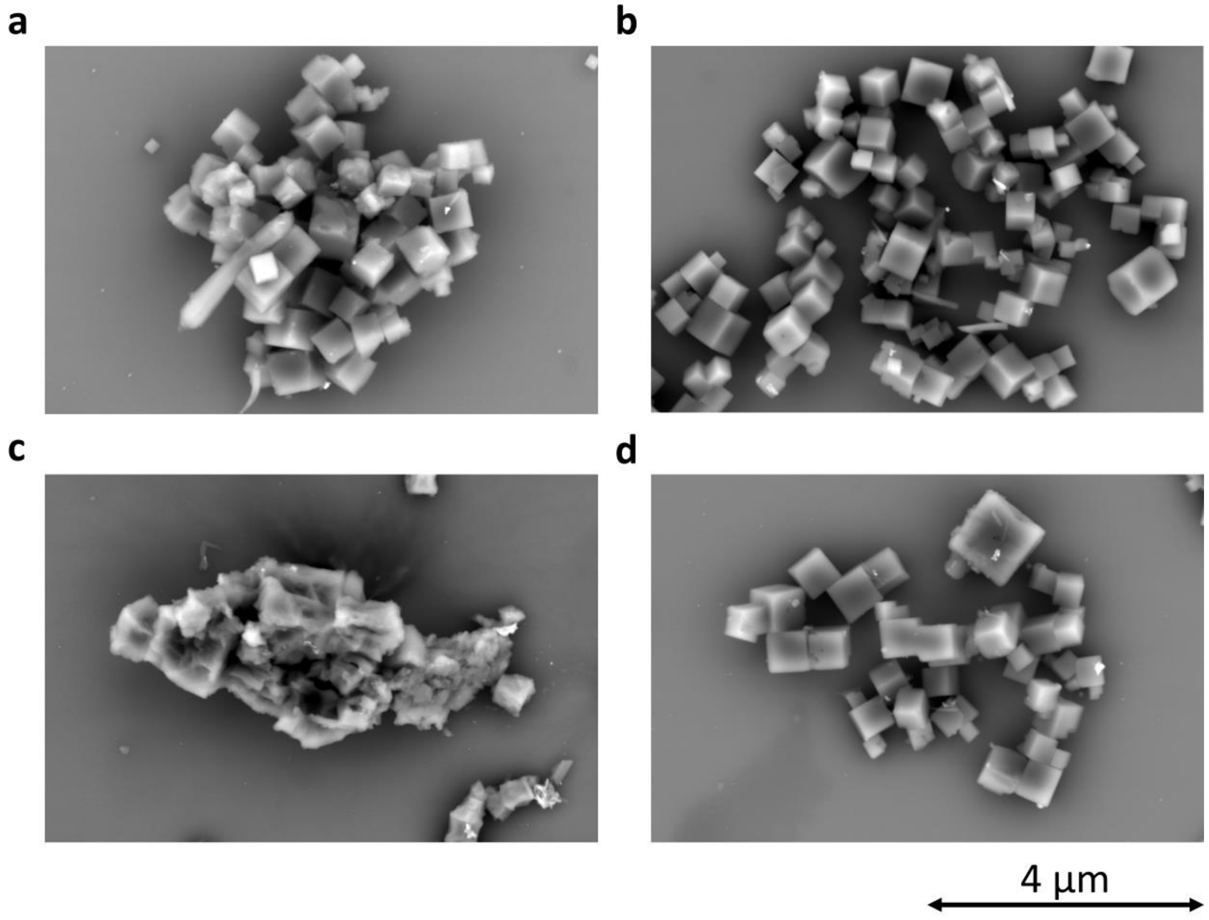

Figure S48: Scanning electron microscopy image of (a) PCN-224, (b) PCN-224+DPPA before and (c) PCN-224 and (d) PCN-224+DPPA after 24 h exposure to 92% relative humidity.

Proton conductivity

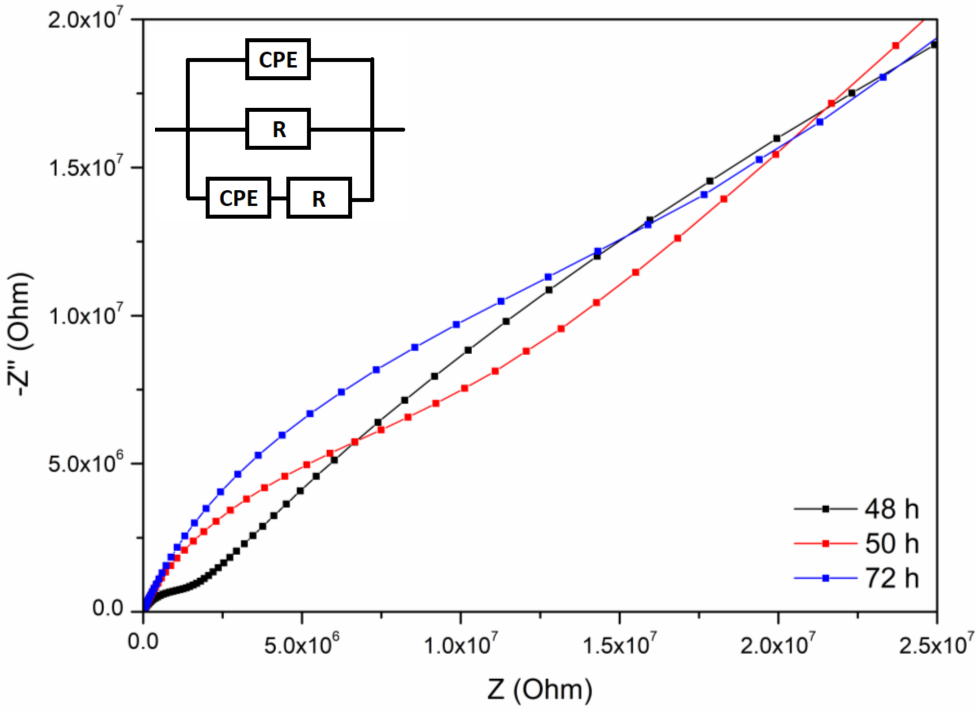

Figure S49: The Nyquist plots for PCN-222 measured at the relative humidity of 75% after 48, 50, and 72 h of exposure to the target air humidity. The equivalent circuit used for fitting is depicted in an inset figure.

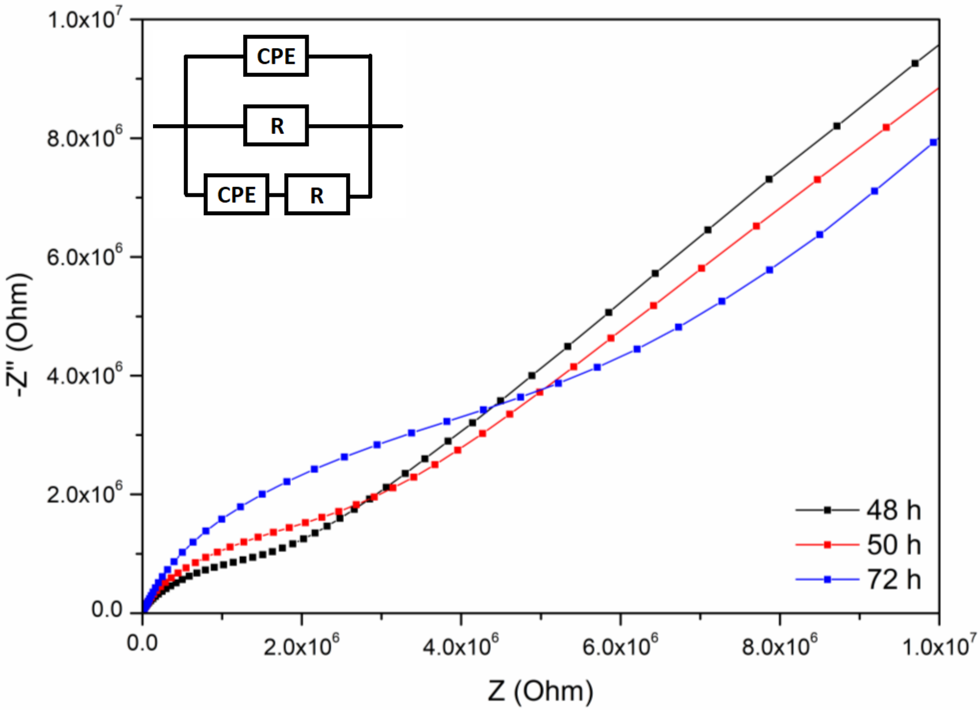

Figure S50: The Nyquist plots for Im@PCN-222 measured at the relative humidity of 75% after 48, 50, and 72 h of exposure to the target air humidity. The equivalent circuit used for fitting is depicted in an inset figure.

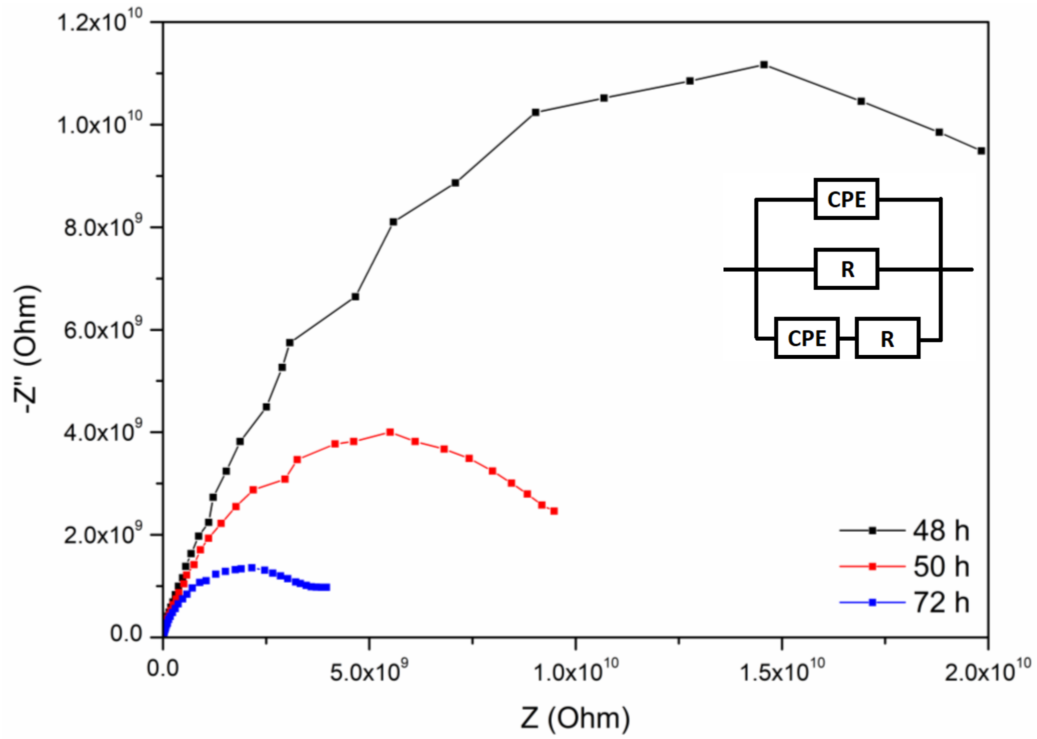

Figure S51: The Nyquist plots for PCN-222+DPPA measured at the relative humidity of 75% after 48, 50, and 72 h of exposure to the target air humidity. The equivalent circuit used for fitting is depicted in an inset figure.

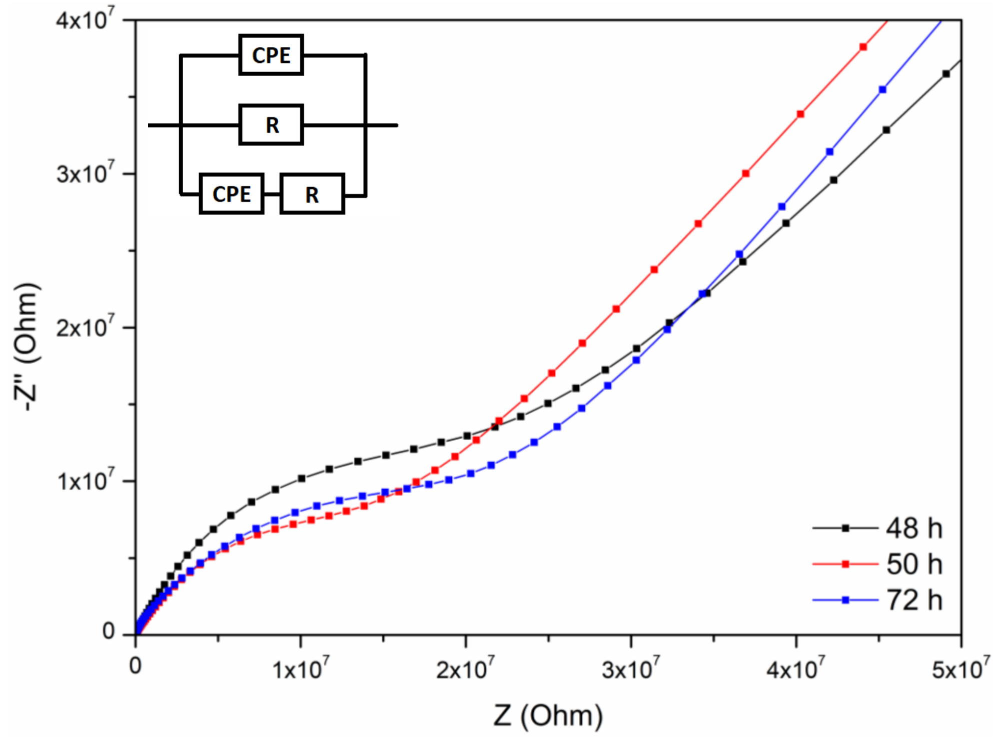

Figure S52: The Nyquist plots for Im@PCN-222+DPPA measured at the relative humidity of 75% after 48, 50, and 72 h of exposure to the target air humidity. The equivalent circuit used for fitting is depicted in an inset figure.

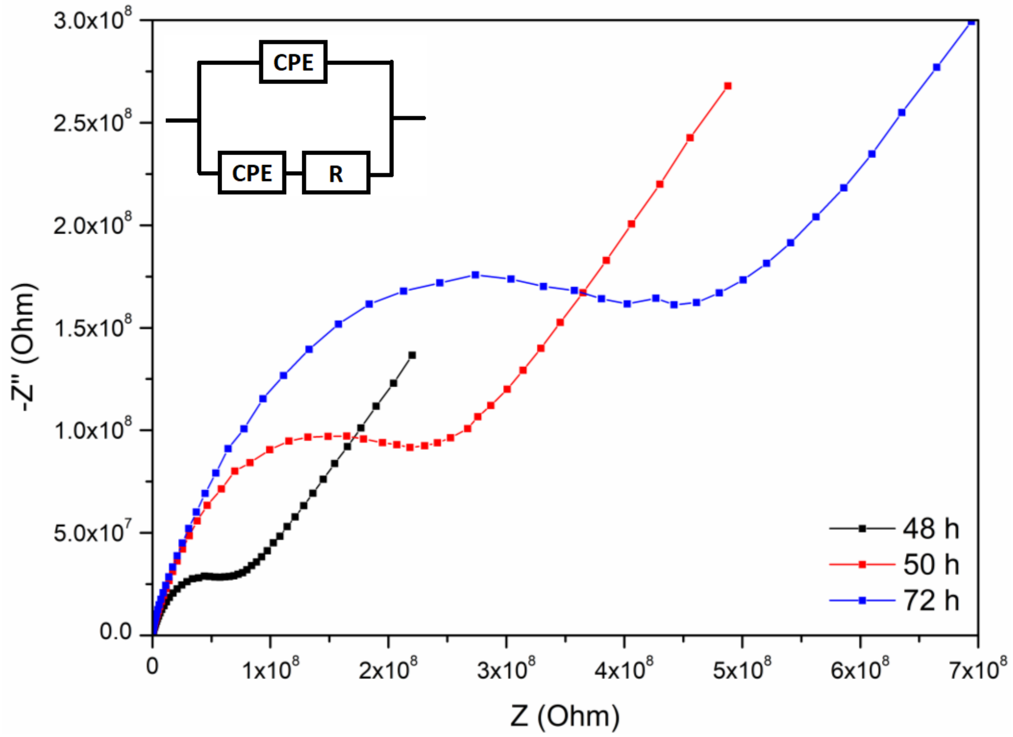

Figure S53: The Nyquist plots for PCN-224 measured at the relative humidity of 75% after 48, 50, and 72 h of exposure to the target air humidity. The equivalent circuit used for fitting is depicted in an inset figure.

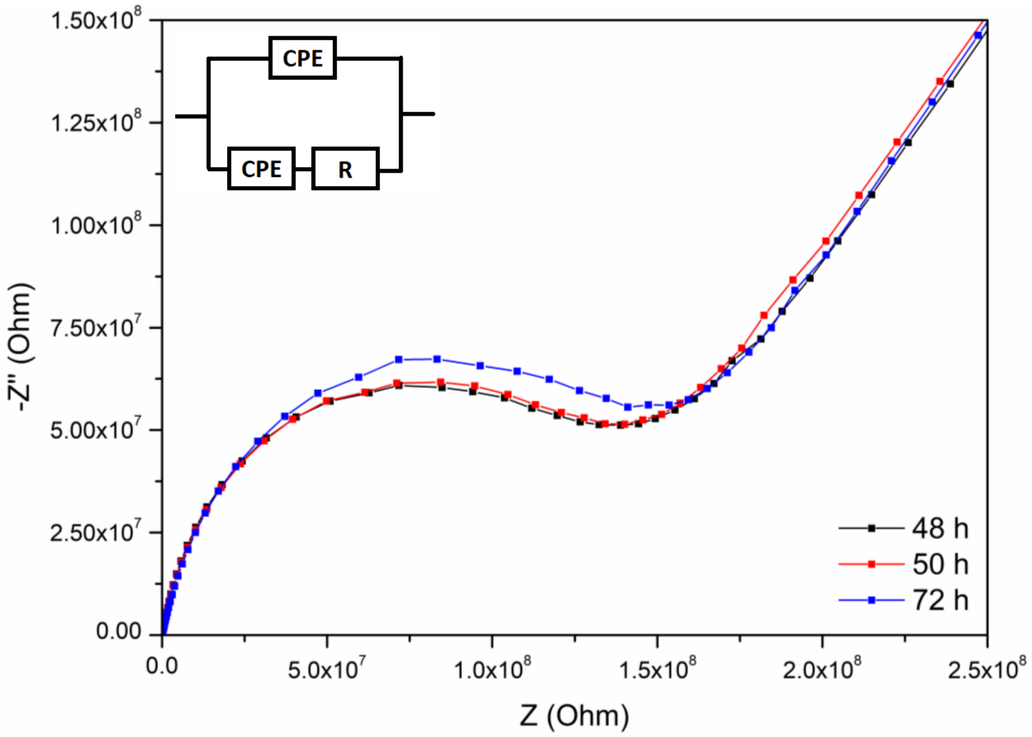

Figure S54: The Nyquist plots for Im@PCN-224 measured at the relative humidity of 75% after 48, 50, and 72 h of exposure to the target air humidity. The equivalent circuit used for fitting is depicted in an inset figure.

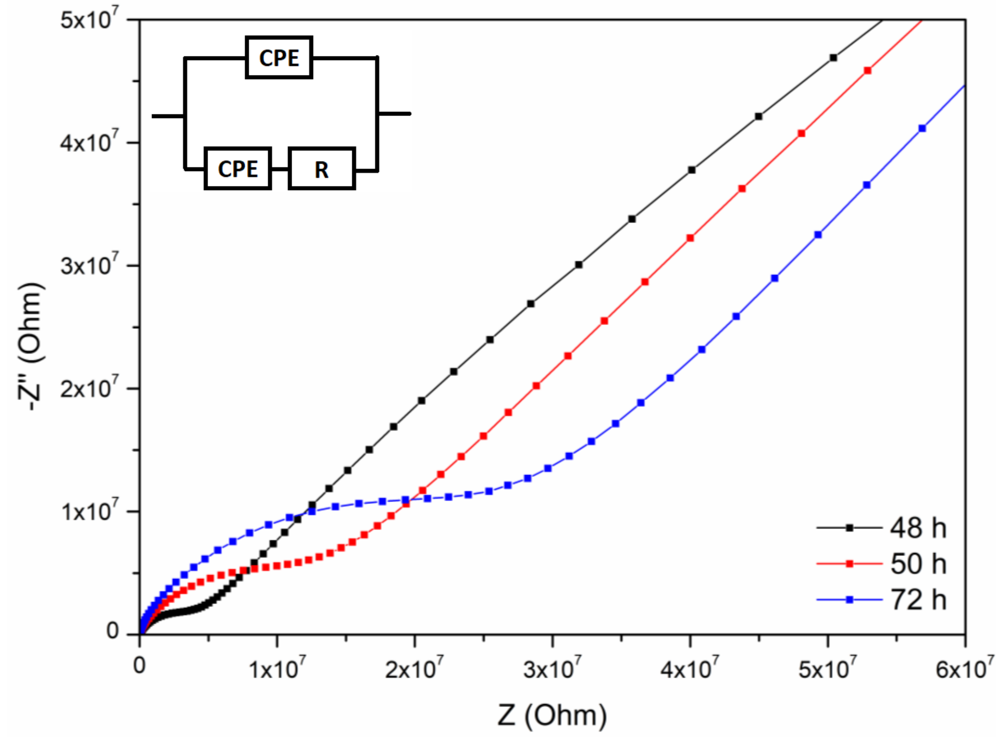

Figure S55: The Nyquist plots for PCN-224+DPPA measured at the relative humidity of 75% after 48, 50, and 72 h of exposure to the target air humidity. The equivalent circuit used for fitting is depicted in an inset figure.

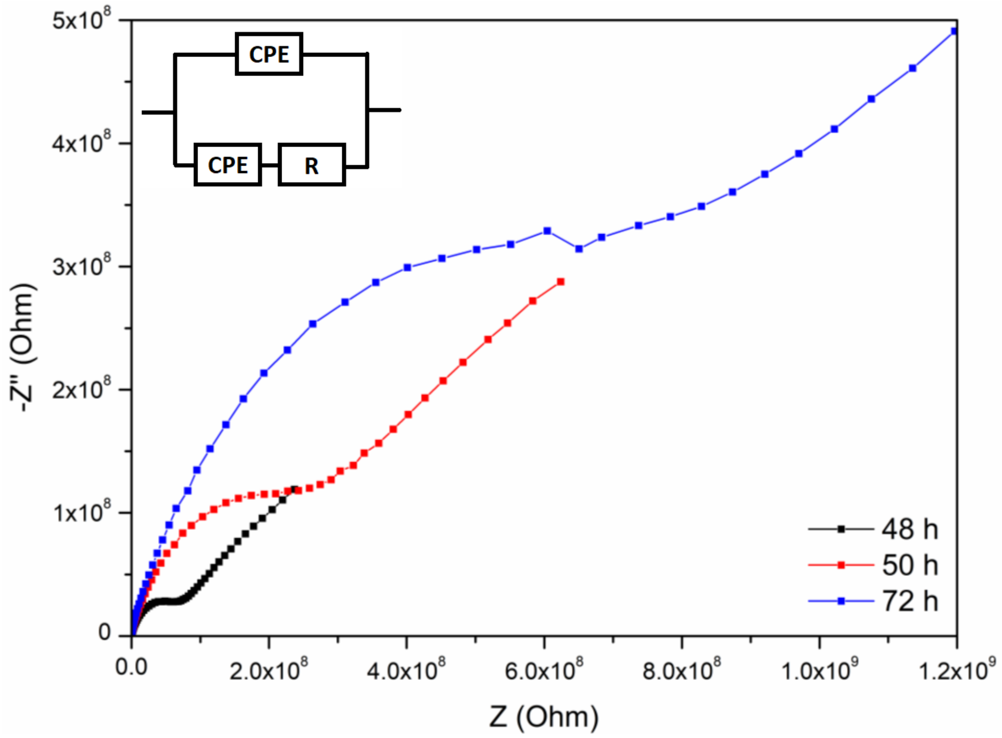

Figure S56: The Nyquist plots for Im@PCN-224+DPPA measured at the relative humidity of 75% after 48, 50, and 72 h of exposure to the target air humidity. The equivalent circuit used for fitting is depicted in an inset figure.

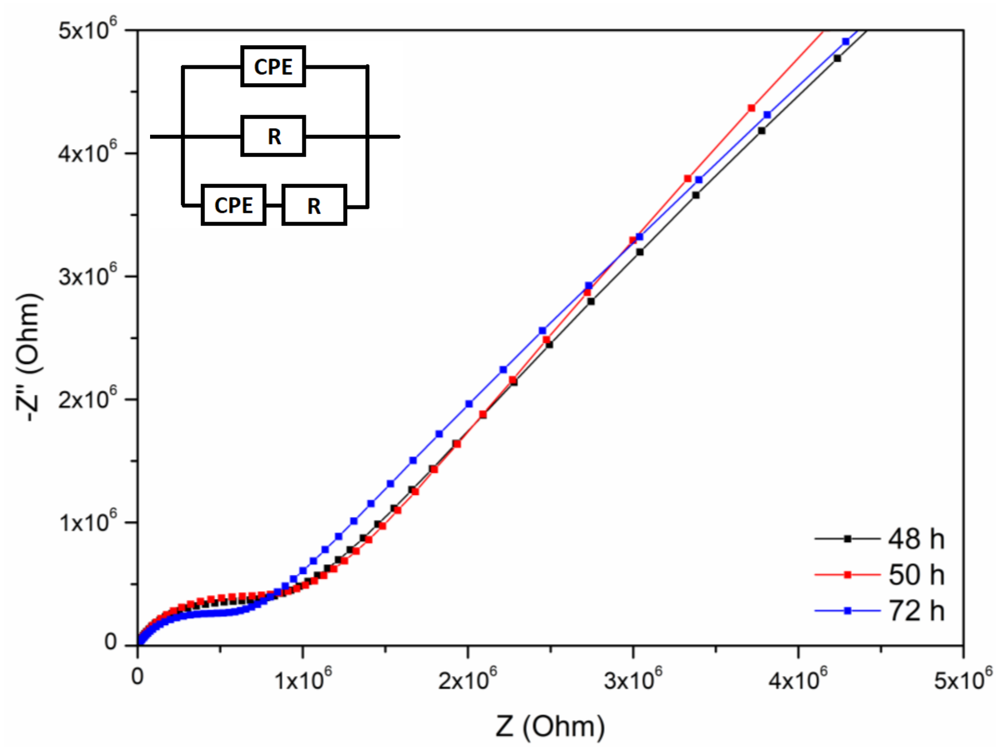

Figure S57: The Nyquist plots for PCN-222 measured at the relative humidity of 92% after 48, 50, and 72 h of exposure to the target air humidity. The equivalent circuit used for fitting is depicted in an inset figure.

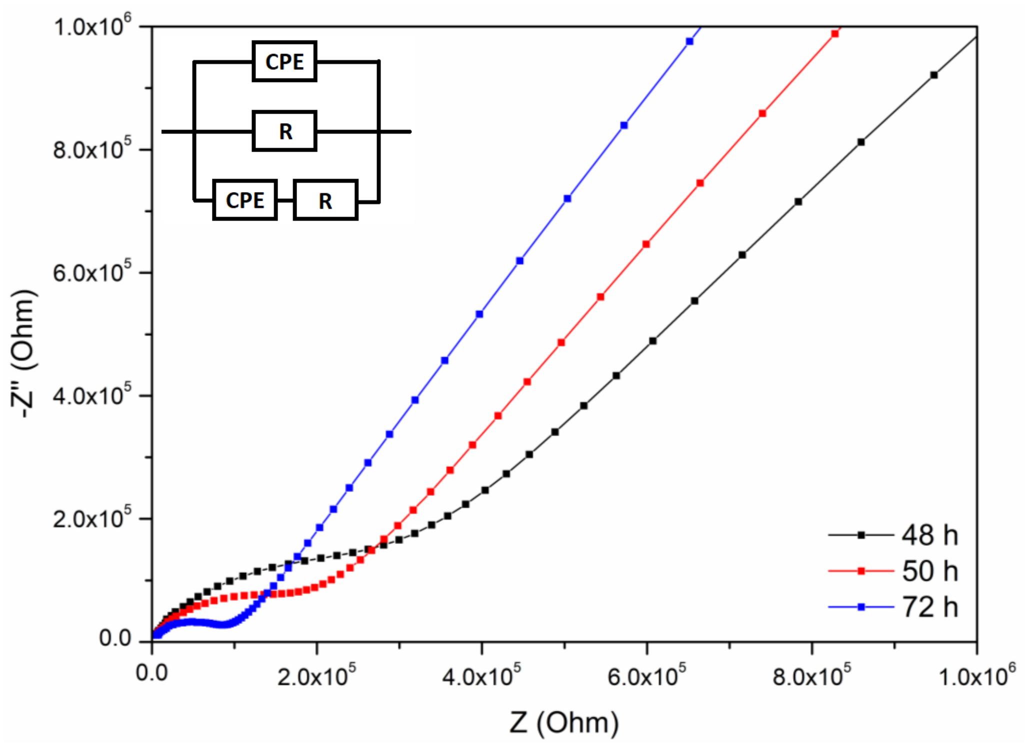

Figure S58: The Nyquist plots for Im@PCN-222 measured at the relative humidity of 92% after 48, 50, and 72 h of exposure to the target air humidity. The equivalent circuit used for fitting is depicted in an inset figure.

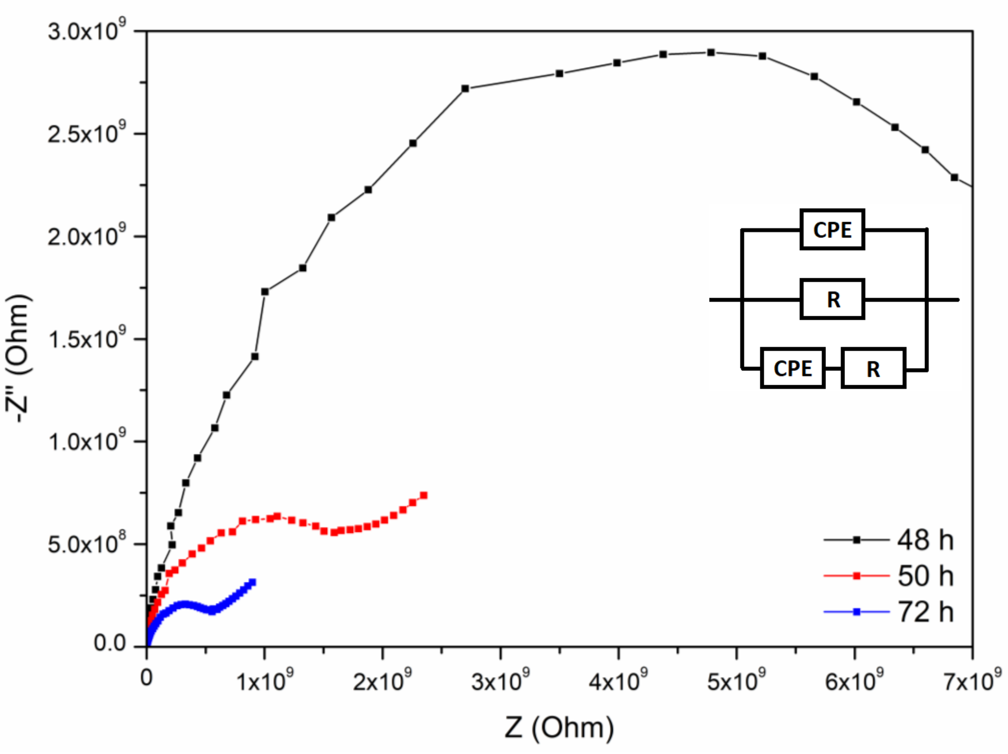

Figure S59: The Nyquist plots for PCN-222+DPPA measured at the relative humidity of 92% after 48, 50, and 72 h of exposure to the target air humidity. The equivalent circuit used for fitting is depicted in an inset figure.

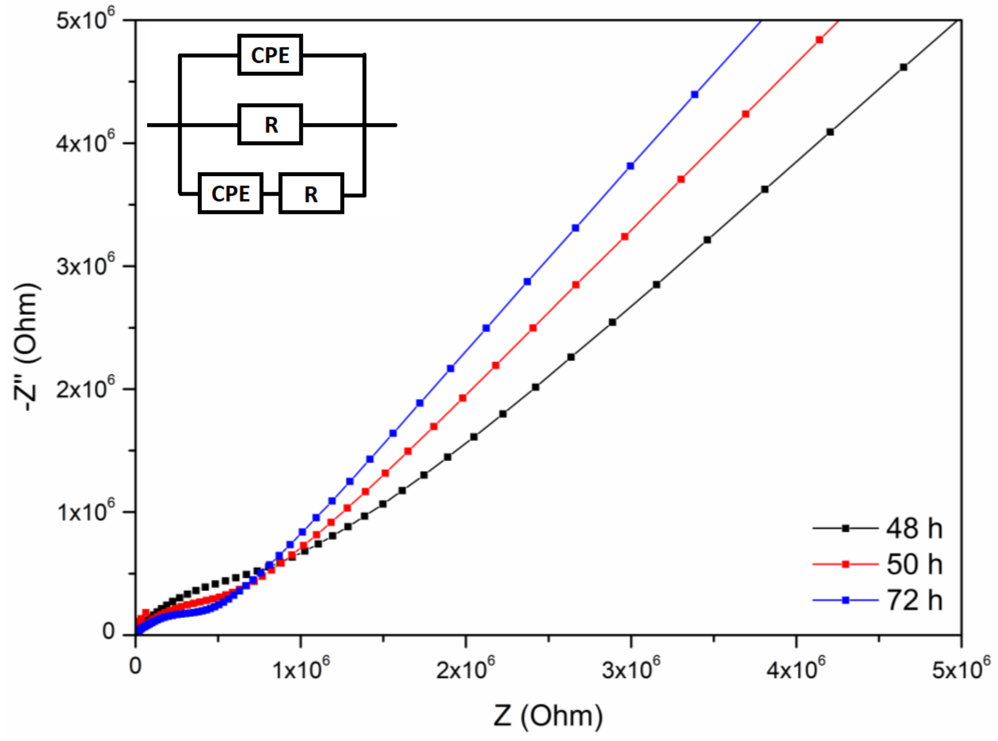

Figure S60: The Nyquist plots for Im@PCN-222+DPPA measured at the relative humidity of 92% after 48, 50, and 72 h of exposure to the target air humidity. The equivalent circuit used for fitting is depicted in an inset figure.

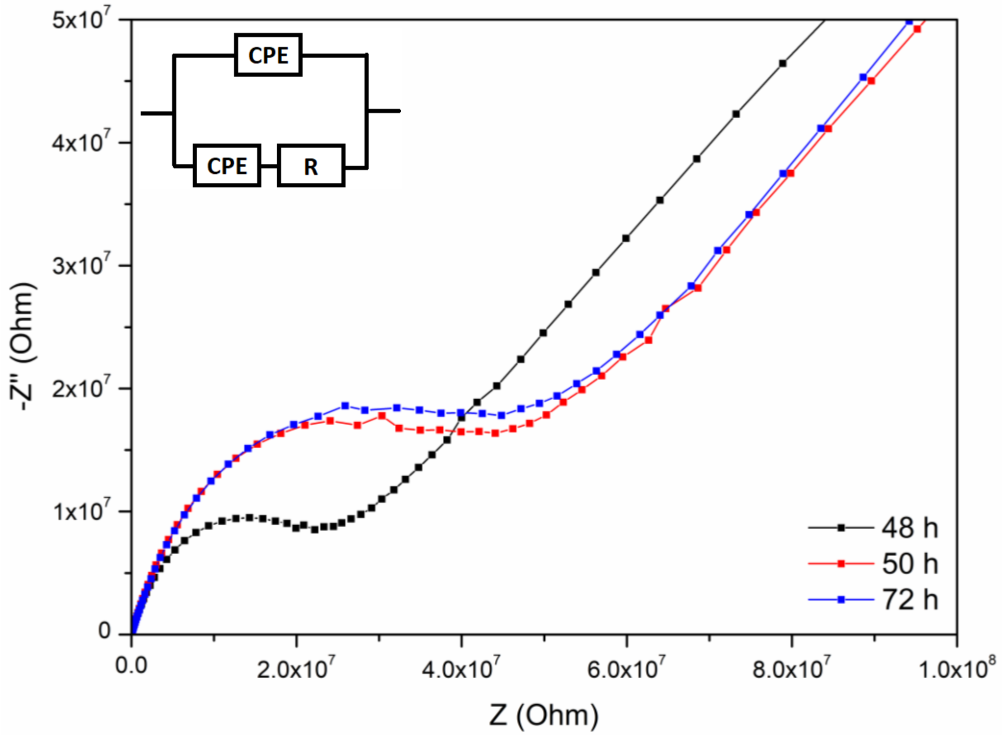

Figure S61: The Nyquist plots for PCN-224 measured at the relative humidity of 92% after 48, 50, and 72 h of exposure to the target air humidity. The equivalent circuit used for fitting is depicted in an inset figure.

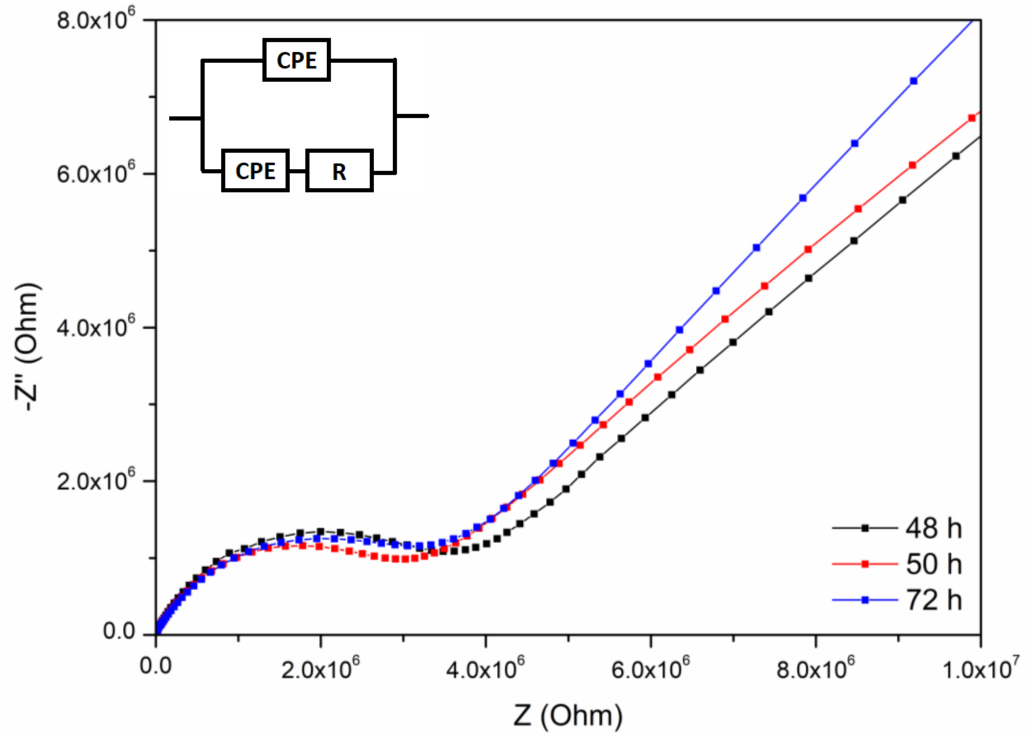

Figure S62: The Nyquist plots for Im@PCN-224 measured at the relative humidity of 92% after 48, 50, and 72 h of exposure to the target air humidity. The equivalent circuit used for fitting is depicted in an inset figure.

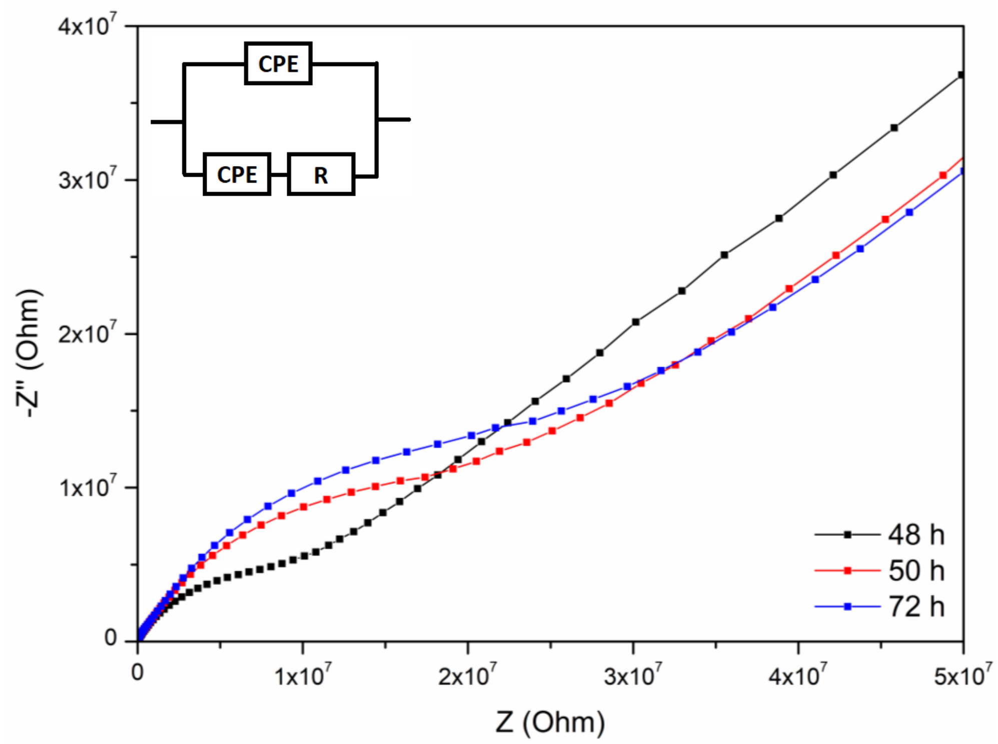

Figure S63: The Nyquist plots for PCN-224+DPPA measured at the relative humidity of 92% after 48, 50, and 72 h of exposure to the target air humidity. The equivalent circuit used for fitting is depicted in an inset figure.

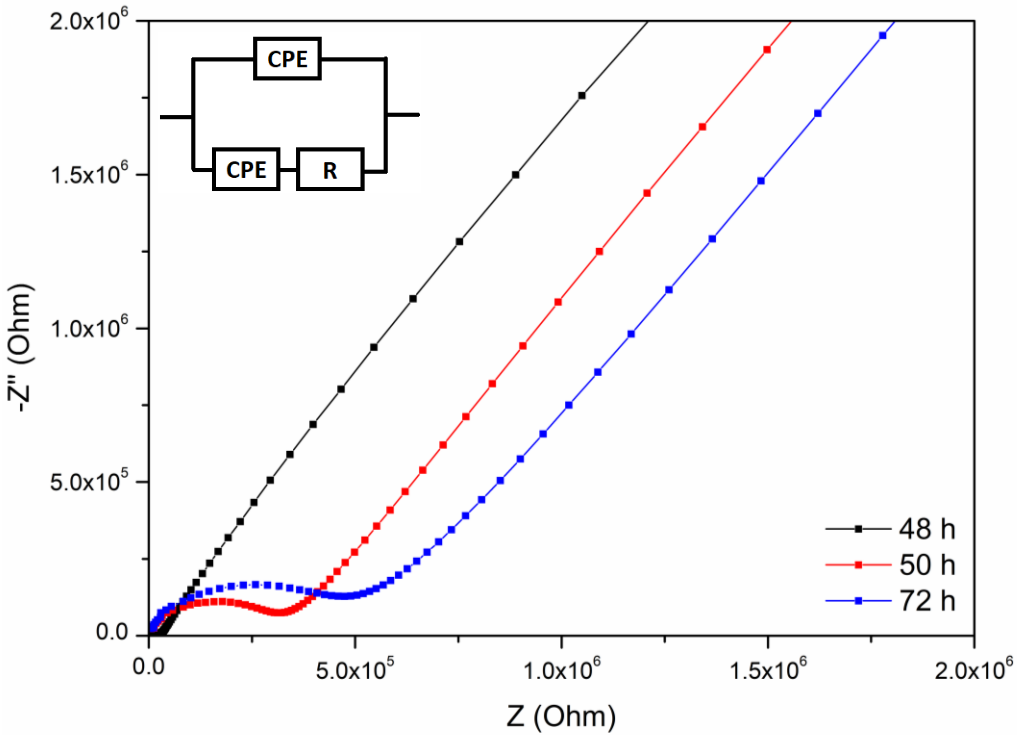

Figure S64: The Nyquist plots for Im@PCN-224+DPPA measured at the relative humidity of 92% after 48, 50, and 72 h of exposure to the target air humidity. The equivalent circuit used for fitting is depicted in an inset figure.

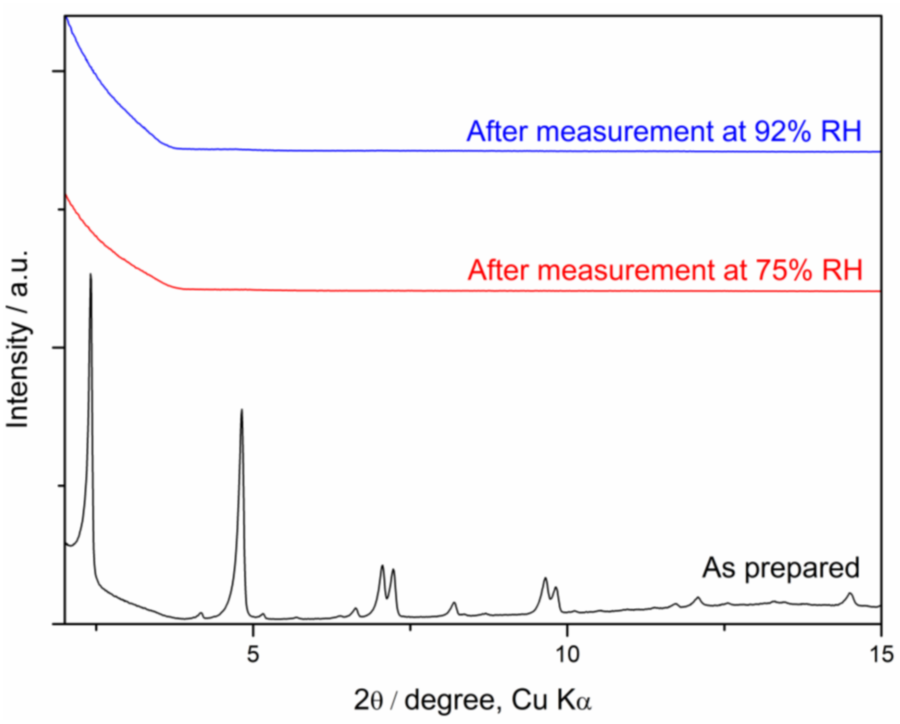

Figure S65: Powder XRD patterns of PCN-222 before (below) and after the measurement of proton conductivity at 75% (middle) and 92% (top) relative humidity. Diffractograms are shifted vertically to avoid overlaps.

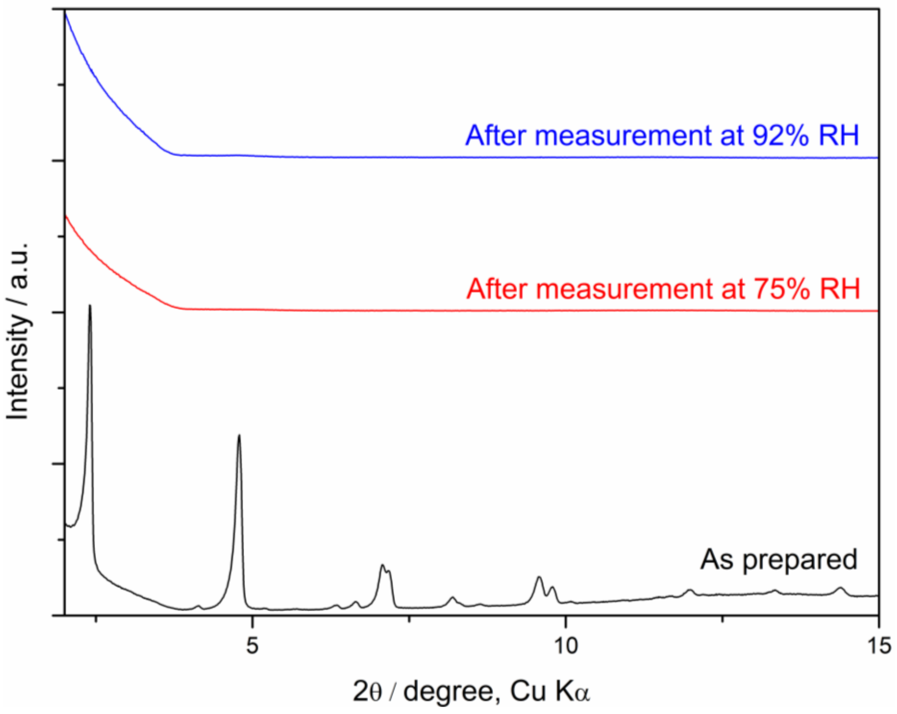

Figure S66: Powder XRD patterns of Im@PCN-222 before (below) and after the measurement of proton conductivity at 75% (middle) and 92% (top) relative humidity. Diffractograms are shifted vertically to avoid overlaps.

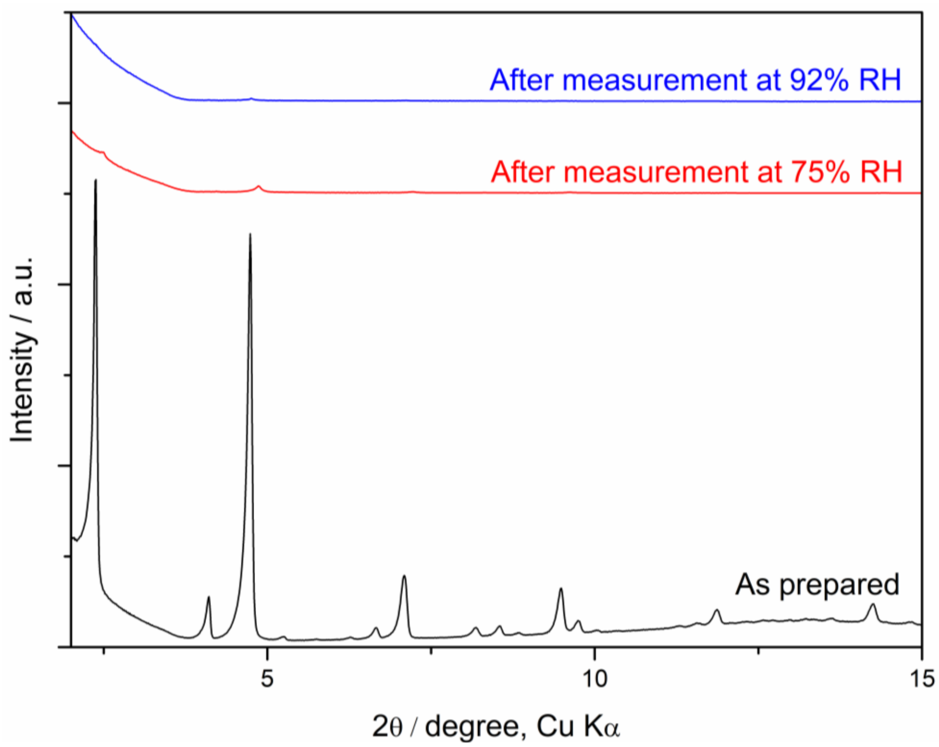

Figure S67: Powder XRD patterns of PCN-222+DPPA before (below) and after the measurement of proton conductivity at 75% (middle) and 92% (top) relative humidity. Diffractograms are shifted vertically to avoid overlaps.

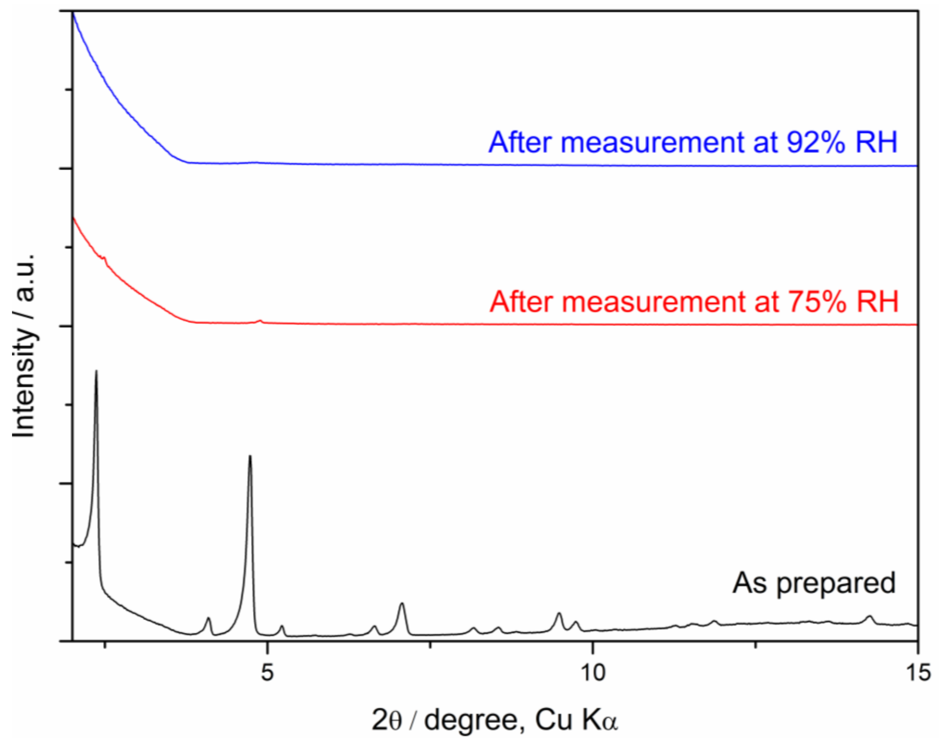

Figure S68: Powder XRD patterns of Im@PCN-222+DPPA before (below) and after the measurement of proton conductivity at 75% (middle) and 92% (top) relative humidity. Diffractograms are shifted vertically to avoid overlaps.

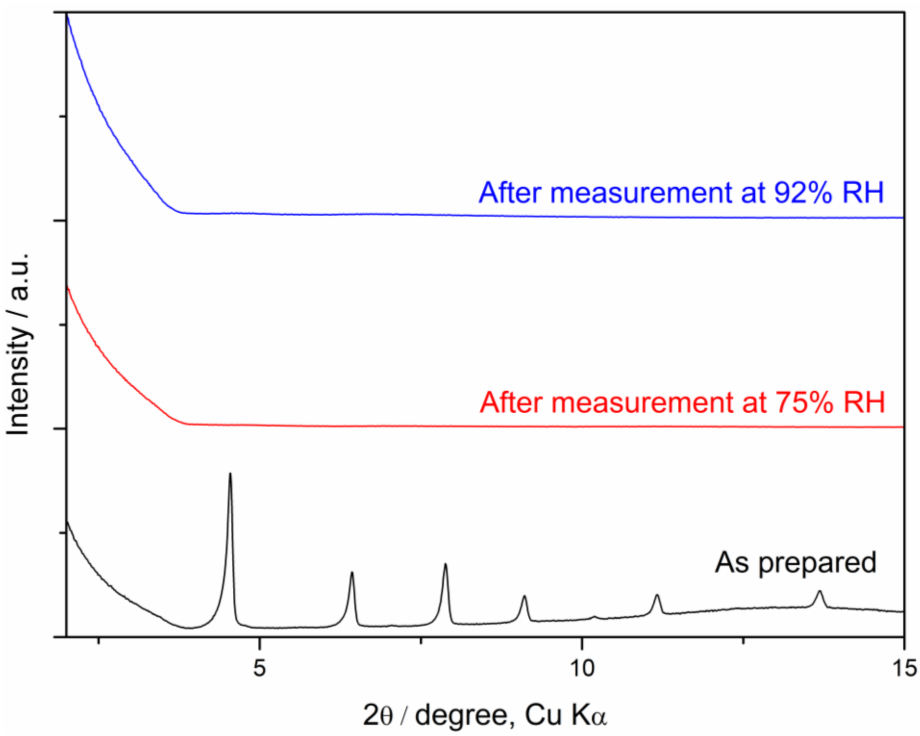

Figure S69: Powder XRD patterns of PCN-224 before (below) and after the measurement of proton conductivity at 75% (middle) and 92% (top) relative humidity. Diffractograms are shifted vertically to avoid overlaps.

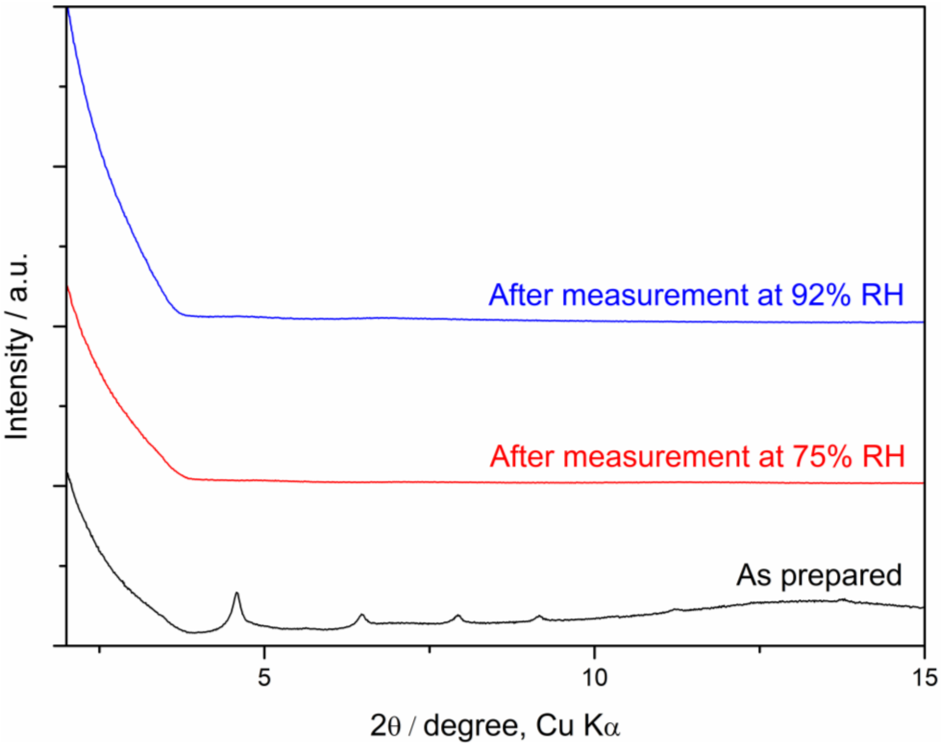

Figure S70: Powder XRD patterns of Im@PCN-224 before (below) and after the measurement of proton conductivity at 75% (middle) and 92% (top) relative humidity. Diffractograms are shifted vertically to avoid overlaps.

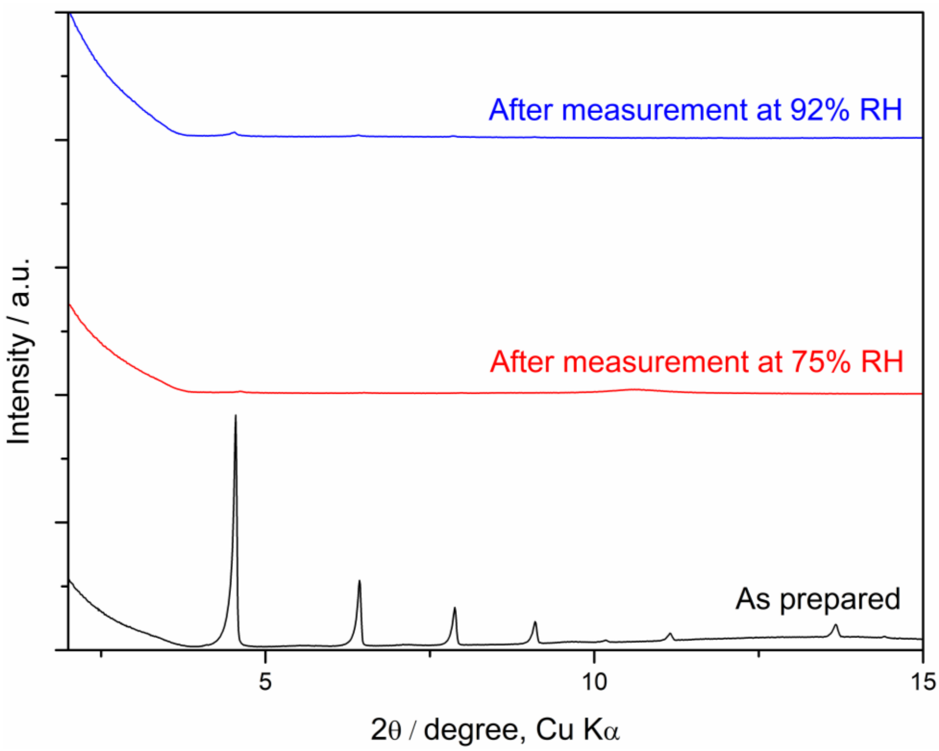

Figure S71: Powder XRD patterns of PCN-224+DPPA before (below) and after the measurement of proton conductivity at 75% (middle) and 92% (top) relative humidity. Diffractograms are shifted vertically to avoid overlaps.

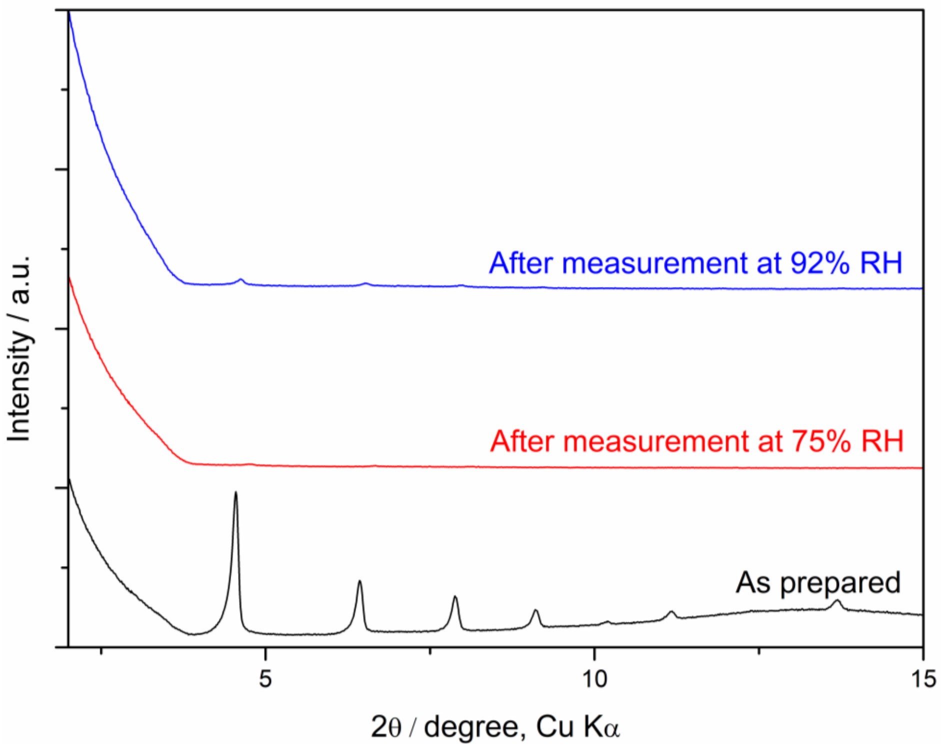

Figure S72: Powder XRD patterns of Im@PCN-222+DPPA before (below) and after the measurement of proton conductivity at 75% (middle) and 92% (top) relative humidity. Diffractograms are shifted vertically to avoid overlaps.

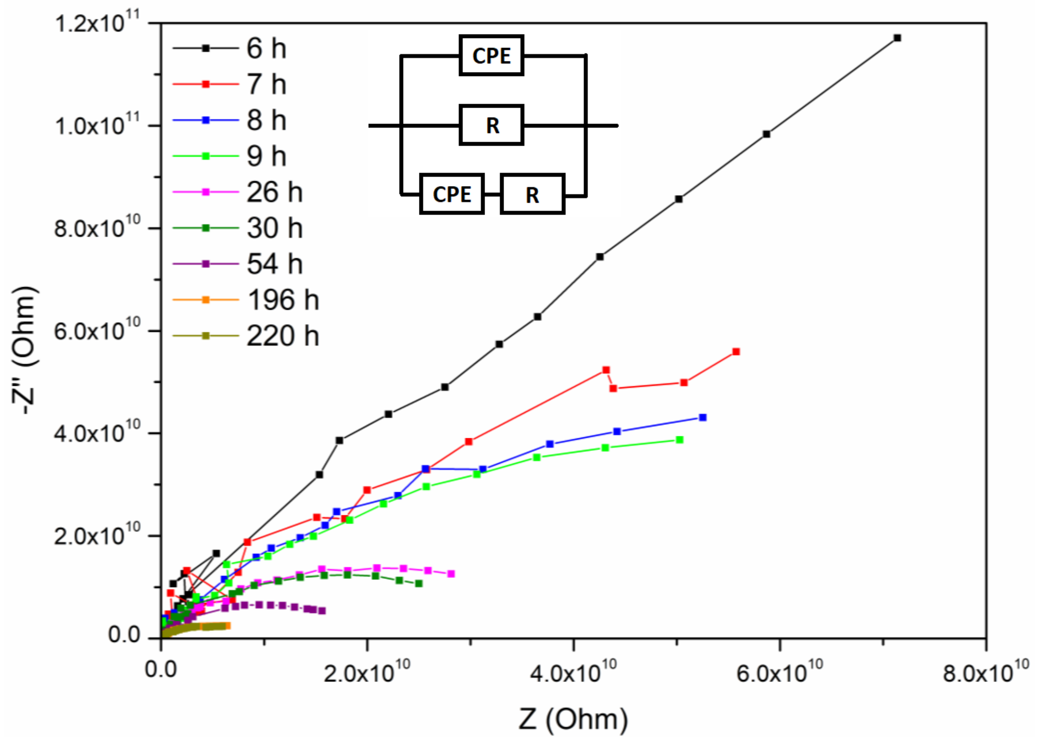

Figure S73: The Nyquist plots for PCN-222+DPPA measured at the relative humidity of 75% after different times of exposure to the target air humidity with the 6 h incubation before the pellet preparation. The equivalent circuit used for fitting is depicted in an inset figure.

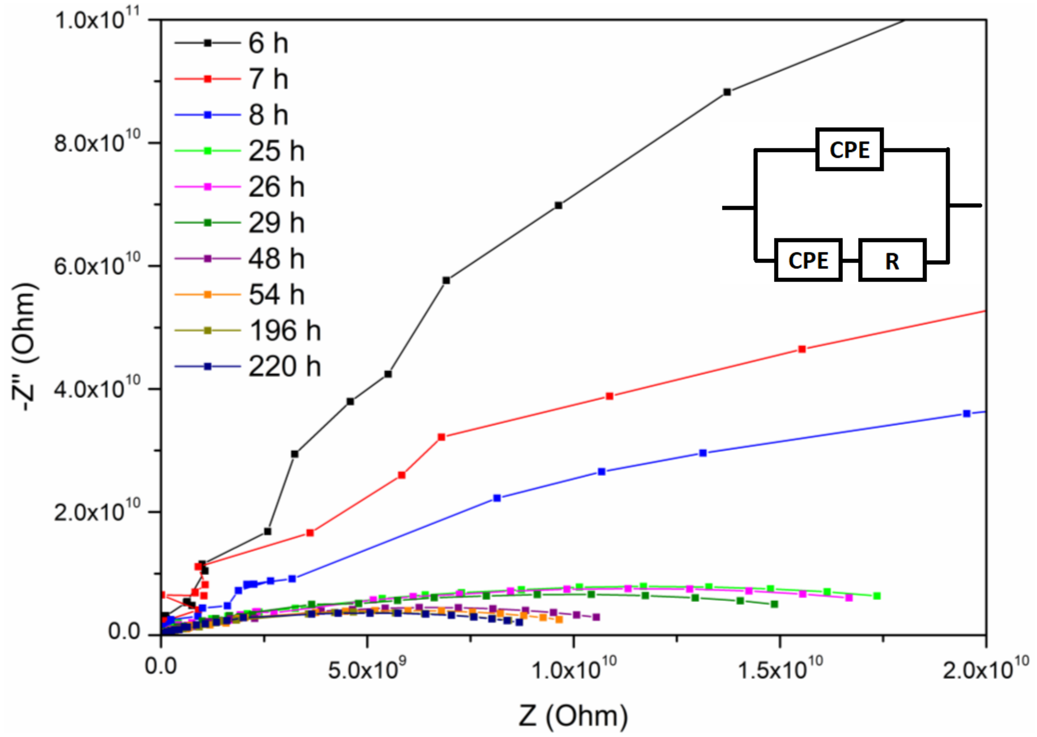

Figure S74: The Nyquist plots for PCN-224+DPPA measured at the relative humidity of 75% after different times of exposure to the target air humidity with the 6 h incubation before the pellet preparation. The equivalent circuit used for fitting is depicted in an inset figure.

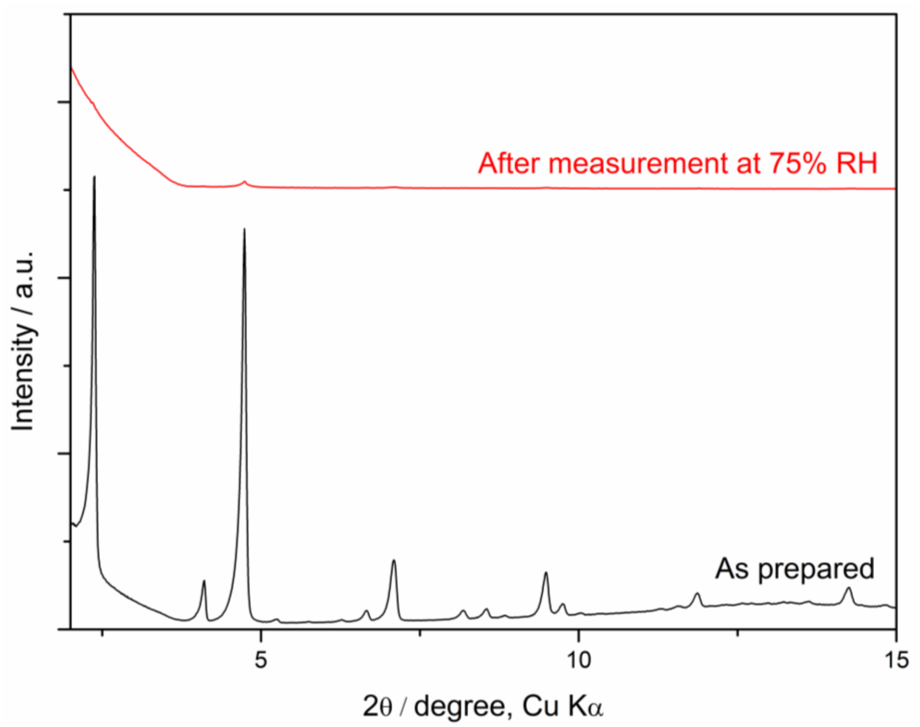

Figure S75: Powder XRD patterns of PCN-222+DPPA before (below) and after the measurement of proton conductivity (top) at 75% relative humidity with the 6 h incubation before the pellet preparation. Diffractograms are shifted vertically to avoid overlaps.

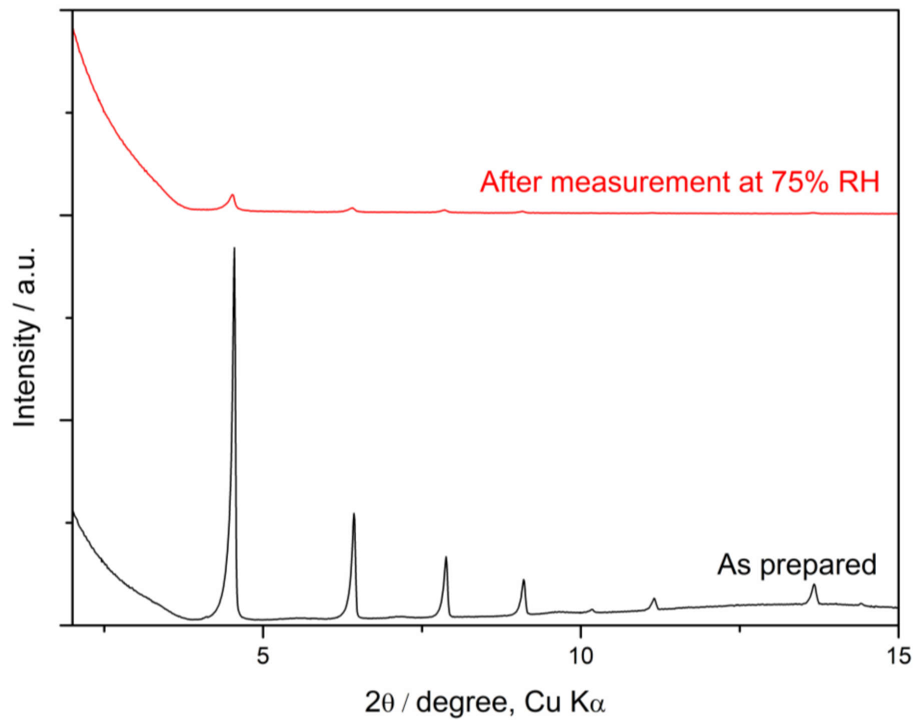

Figure S76: Powder XRD patterns of PCN-224+DPPA before (below) and after the measurement of proton conductivity (top) at 75% relative humidity with the 6 h incubation before the pellet preparation. Diffractograms are shifted vertically to avoid overlaps.

Table S5: Comparison of the proton conductivity measured on PCN-222+DPPA and PCN-224+DPPA after defined time of exposure to 75 % relative humidity with 6 h vs. 48 h incubation of the sample before the preparation of pellets.

**PCN-222+DPPA**			**PCN-224+DPPA**		
Time / h	6 h incubation	48 h incubation	Time / h	6 h incubation	48 h incubation
6	2.2E-12		6	8.4E-13	-
7	5.6E-12		7	2.8E-12	-
8	6E-12		8	5.1E-12	-
9	6.6E-12		25	3.0E-12	-
26	1.4E-11		26	3.1E-12	-
30	1.8E-11		29	3.7E-11	-
48	-	2.1E-11	48	5.6E-11	1.6E-8
50	-	6.4E-11	50	-	3.9E-9
54	3E-11		54	6.2E-11	-
72	-	1.5E-10	72	-	1.4E-9
196	8.6E-11		196	7.0E-11	-
220	9.6E-11		220	7.1E-11	-

Table S6: Comparison of the proton conductivity of different Zr(IV)-MOFs measured under conditions similar to ours.

Material	Conditions of measurement	Proton conductivity / S·cm^{-1}	REF.
PCN-222	298 K, 75% RH	4.2E-08	-
	298 K, 92% RH	9.4E-07	
Im@PCN-222	298 K, 75% RH	1.1E-07	-
	298 K, 92% RH	6.7E-06	
PCN-224	298 K, 75% RH	2.3E-09	-
	298 K, 92% RH	1.7E-08	
Im@PCN-224	298 K, 75% RH	3.9E-09	-
	298 K, 92% RH	1.7E-07	
UiO-66	303 K, 85% RH	3.2E-08	11
UiO-66-$(CO_2H)_2$	303 K, 85% RH	1.3E-04	
UiO-66-(SO_3H)	303 K, 85% RH	5.0E-05	
	303 K, 97% RH	3.4E-03	
MOF-808	298 K, 67% RH	3.9E-05	12
	298 K, 84% RH	2.0E-04	
	315 K, 99% RH	7.6E-03	
Im@MOF-808	288 K, 99% RH	8.9E-03	13
	313 K, 99% RH	2.0E-02	

Literature

1 L. Valenzano, B. Civalleri, S. Chavan, S. Bordiga, M. H. Nilsen, S. Jakobsen, K. P. Lillerud, C. Lamberti, Disclosing the Complex Structure of UiO-66 Metal Organic Framework: A Synergic Combination of Experiment and Theory, *Chem. Mater.* **2011**, *23*, 1700.

2 G. Socrates*, Infrared and Raman characteristic group frequencies. Tables and charts*, John Wiley and Sons, Chichester, Third Edition, *2001*.

3 X. Chen, Y. Lyu, Z. Wang, X. Qiao, B. C. Gates, D. Yang, Tuning $Zr_{12}O_{22}$ Node Defects as Catalytic Sites in the Metal-Organic Framework Hcp UiO-66, *ACS Catal*. **2020**, *10*, 2906.

4 M. Babucci, A. S. Hoffman, S. R. Bare, B. C. Gates, Characterization of a Metal-Organic Framework Zr_6O_8 Node-Supported Atomically Dispersed Iridium Catalyst for Ethylene Hydrogenation by X-Ray Absorption Near-Edge Structure and Infrared Spectroscopies, *J. Phys. Chem. C* **2021**, *125*, 16995.

5 R. Wei, C. A. Gaggioli, G. Li, T. Islamoglu, Z. Zhang, P. Yu, O. K. Farha, C. J. Cramer, L. Gagliardi, D. Yang, B. C. Gates, Tuning the Properties of Zr_6O_8 Nodes in the Metal Organic Framework UiO-66 by Selection of Node-Bound Ligands and Linkers, *Chem. Mater*. **2019**, *31*, 1655.

6 K. Chakarova, I. Strauss, M. Mihaylov, N. Drenchev, K. Hadjiivanov, Evolution of Acid and Basic Sites in UiO-66 and UiO-66-NH_2 Metal-Organic Frameworks: FTIR Study by Probe Molecules, *Microporous Mesoporous Mater.* **2019**, *281*, 110.

7 A. D. Wiersum, E. Soubeyrand-Lenoir, Q. Yang, B. Moulin, V. Guillerm, M. B. Yahia, S. Bourrelly, A. Vimont, S. Miller, C. Vagner, M. Daturi, G. Clet, C. Serre, G. Maurin, P. L. Llewellyn, An Evaluation of UiO-66 for Gas-Based Applications, Chem. Asian J. **2011**, *6*, 3270.

8 M. Aydin, DFT and Raman Spectroscopy of Porphyrin Derivatives: Tetraphenylporphine (TPP), *Vibr. Spectrosc.* **2013**, *68*, 141.

9 M. Aydin, Comparative Study of the Structural and Vibroelectronic Properties of Porphyrin and Its Derivatives, *Molecules* **2014**, *19*, 20988.

10 G. C. Shearer, S. Chavan, J. Ethiraj, J. G. Vitillo, S. Svelle, U. Olsbye, C. Lamberti, S. Bordiga, K. P. Lillerud, Tuned to Perfection: Ironing out the Defects in Metal-Organic Framework UiO-66, *Chem. Mater.* **2014**, *26*, 4068.

11 F. Yang, H. Huang, X. Wang, F. Li, Y. Gong, C. Zhong, J. R. Li, Proton Conductivities in Functionalized UiO-66: Tuned Properties, Thermogravimetry Mass, and Molecular Simulation Analyses, *Cryst. Growth Des.* **2015**, *15*, 5827.

12 H. Luo, M. Wang, S. Liu, C. Xue, Z. Tian, Y. Zou, X. Ren, Proton Conductance of a Superior Water-Stable Metal-Organic Framework and Its Composite Membrane with Poly(vinylidene fluoride) *Inorg. Chem.* **2017**, *56*, 4169.

13 H.-B. Luo, Q. Ren, P. Wang, J. Zhang, L. Wang, X.-M. Ren, High Proton Conductivity Achieved by Encapsulation of Imidazole Molecules into Proton-Conducting MOF-808, *ACS Appl. Mater. Interfaces* **2019**, *11*, 9164.
